# Stable Hemiaminals: 2-Aminopyrimidine Derivatives

**DOI:** 10.3390/molecules200814365

**Published:** 2015-08-06

**Authors:** Anna Kwiecień, Zbigniew Ciunik

**Affiliations:** Faculty of Chemistry, University of Wrocław, F. Joliot-Curie 14, 50-383 Wrocław, Poland; E-Mail: ciunik@wchuwr.pl

**Keywords:** stable hemiaminal, stable intermediate, crystal structure, 2-aminopyrimidine, nitrobenzaldehyde

## Abstract

Stable hemiaminals can be obtained in the one-pot reaction between 2-aminopyrimidine and nitrobenzaldehyde derivatives. Ten new hemiaminals have been obtained, six of them in crystal state. The molecular stability of these intermediates results from the presence of both electron-withdrawing nitro groups as substituents on the phenyl ring and pyrimidine ring, so no further stabilisation by intramolecular interaction is required. Hemiaminal molecules possess a tetrahedral carbon atom constituting a stereogenic centre. As the result of crystallisation in centrosymmetric space groups both enantiomers are present in the crystal structure.

## 1. Introduction

**Scheme 1:**
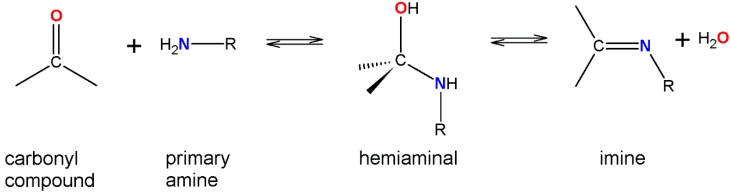
Reaction scheme between carbonyl compounds and primary amines.

Hemiaminals are intermediate products in the process of forming imines from carbonyl compounds (aldehydes or ketones) and primary amines (Scheme 1). In a similar way hemiaminals are also formed from secondary amines.

When obtained from aldehydes and primary amines, hemiaminals are highly unstable [1] and thus can only be observed under special conditions. Isolation from the external environment is one of the factors stabilising hemiaminal moieties. Such seclusion can be achieved by means of conducting reactions inside a macromolecular synthetic receptor [2,3,4] or within metal-organic frameworks [5,6]. A hemiaminal was detected as a short-lived intermediate in the solvent-free *mechanochemical reaction* between *o*-vanillin and *p*-toluidine [7]. Kinetically trapped hemiaminals were also observed in polymeric materials such as hemiaminal dynamic covalent networks [8,9], formed by water-promoted stepwise condensations [10]. Other sophisticated methods enabling analysis of hemiaminals include polarography [11], measurements in FTIR liquid cells [12], spectroscopy at low temperatures [13] and *in situ* low-temperature co-crystallisation of non-solid components [14]. Hemiaminals can be also stabilised by coordinating to metal ions as *O*- or *N*-donor ligands [15,16].

The first hemiaminal stable in a crystalline state was obtained from 4-cyclohexyl-3-thiosemicarbazide and di-2-pyridyl ketone [17]. The molecular structure of the obtained hemiaminal is stabilised by the resulting intramolecular hydrogen bonding scheme. Intramolecular hydrogen bonding could be also responsible for the increased stability of a iodohemiaminal intermediate [18].

In our previous papers [19,20] we have reported that the proper choice of reactants (namely benzaldehyde derivatives with electron-withdrawing substituents and 1,2,4-triazole rings) concerning their electronic properties can also lead to stable hemiaminals.

**Figure 1:**
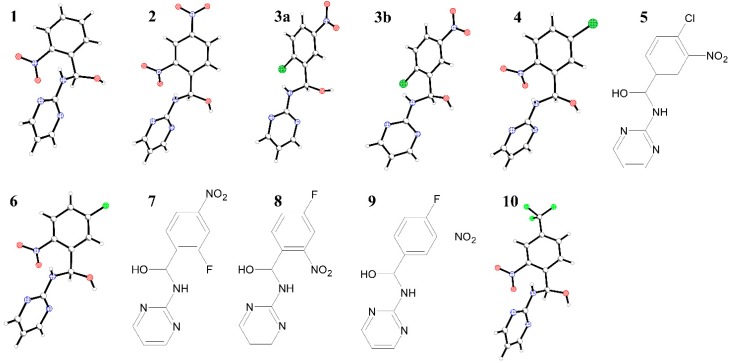
Molecular structures and structural formulas of the obtained hemiaminals.

The aim of this study was to synthesise and spectroscopically and structurally characterise a new class of stable hemiaminal derivatives of 2-aminopyrimidine. Ten new compounds were obtained, six of them in crystalline form suitable for X-ray crystallography. All the compounds were obtained in a simple one-pot reaction from acetonitrile solutions of 2-aminopyrimidine and the appropriate benzaldehyde (2-nitrobenzaldehyde (**1**), 2,4-dinitrobenzaldehyde (**2**), 2-chloro-5-nitrobenzaldehyde (two polymorphic crystals, **3a** and **3b**), 5-chloro-2-nitrobenzaldehyde (**4**), 4-chloro-3-nitrobenzaldehyde (**5**), 5-fluoro-2-nitrobenzaldehyde (**6**), 2-fluoro-4-nitrobenzaldehyde (**7**), 4-fluoro-2-nitrobenzaldehyde (**8**), 4-fluoro-3-nitrobenzaldehyde (**9**), 2-nitro-4-(trifluoromethyl)benzaldehyde (**10**) (Figure 1). All the obtained new organic compounds were characterised by elemental analysis, NMR spectroscopy (^1^H, ^13^C, HMQC, HMBC), IR spectroscopy, ESI-MS and for crystalline state compounds, single crystal X-ray measurements.

## 2. Results and Discussion

### General Remarks

Upon studying the reaction between variously substituted benzaldehydes and aromatic amines we have determined the influence of the electronic properties of the substrates and the reaction conditions on the possibility of obtaining stable hemiaminals or Schiff bases. When the reaction between benzaldehyde derivatives with electron-withdrawing groups (such as nitro- or cyano-) and heterocyclic amines (namely 4-amino-1,2,4-triazole) is conducted under neutral conditions in an aprotic solvent (usually acetonitrile) stable hemiaminals are isolated [19,20]. The corresponding imines are obtained as the only products for electron-donating substituents on the benzaldehyde ring and slightly acidic ethanol solutions [21,22,23,24]. Moreover the change of heterocyclic 4-amino-1,2,4-triazole to an aromatic amine like aniline also leads to formation of the appropriate Schiff base [25].

To further investigate the role of the amine moiety on the nature of the obtained products, reactions between 2-aminopyrimidine and benzaldehydes containing electron-withdrawing nitro groups were performed under neutral conditions. The reaction mixture was typically stirred in acetonitrile at room temperature (or slightly higher, up to 50 °C) for 2 h. The products (compounds **1**, **2**, **3a**, **3b**, **4**, **6** and **10** Figure 1) were obtained as single crystals directly from the mother liquor upon slow solvent evaporation. The remaining compounds **5**, **7**–**9** were deposited from the solution in a non-crystalline state and thus were only characterised by spectroscopic methods.

**Figure 2:**
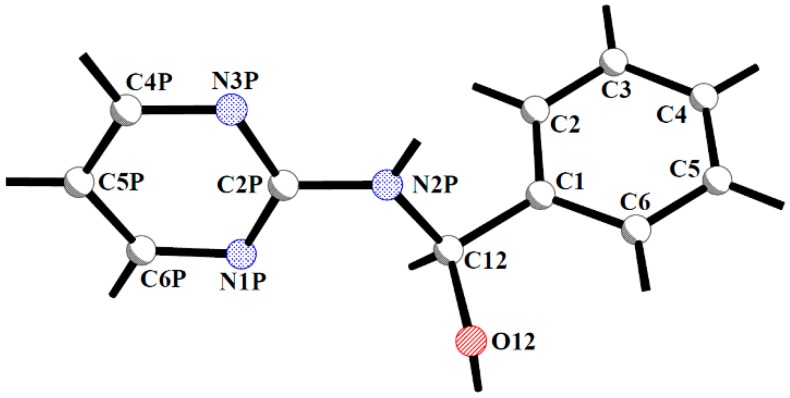
General numbering scheme for hemiaminals with pyrimidine ring.

The molecular structures of hemiaminals are formed by two aromatic rings connected with each other by a C1-C12-N2P-C2P chain of atoms (Figure 2). Furthermore the conformation of the molecule is determined by the value of the torsion angle of this linker. For hemiaminals containing a 1,2,4-triazole ring two conformations are possible in a crystal structure [19,20]. In the stretched molecules the torsion angle on the atomic linkage is close to 180° resulting in an antiperiplanar (*ap*) conformation and the planes of both aromatic rings are almost parallel. For twisted molecules this linkage is in synclinal (*sc*) conformation and the aromatic planes are nearly perpendicular. In the case of hemiaminals with a pyrimidine aromatic ring (except molecule 2 in compound **1**) the linkage is in a ± antiperiplanar conformation, but the values are close to those of anticlinal conformation, which is observed in the second molecule in **1** (Table 1). In contrast to hemiaminals with the triazole ring, the aromatic rings in 2-aminopyrimidine derivatives are screwed, with the values of dihedral angles between phenyl and pyrimidine planes of 67.4(1)°–88.5(1)°. Thus the conformation in this group of hemiaminals can be termed *screwed-stretched*.

In the molecular structures of the presented compounds the characteristic hemiaminal functionality of a tetrahedral carbon (C12) atom connected with hydroxyl group, nitrogen atom (N2P), carbon (C1) atom from the phenyl ring and a hydrogen atom is observed. All the geometrical parameters around the C12 carbon atom are consistent with its *sp^3^* hybridisation (the values of C12-O12 and C12-N2P bond lengths befitting single bonds and a C1-C12-C2P valence angle close to the ideal tetrahedral value, Table 1). All the obtained hemiaminal crystals are centrosymmetric, thus both enantiomers are always present within the lattice.

**Table 1:** Selected geometrical parameters for crystalline hemiaminals (**1**, **2**, **3a**, **3b**, **4**, **6**, **10**).

Compound	Geometrical Parameter								
	Bond Lengths [Å]				Valence Angles [°]		Torsion Angles [°]		Phenyl-Pyrimidine Dihedral Angle [°]
	C1-C12	C12-N2P	N2P-C2P	C12-O12	C1-C12-N2P	C12-N2P-C2P	C2P-N2P-C12-C1	C2P-N2P-C12-O12	
**1**								-	
molecule 1	1.537(2)	1.448(2)	1.358(2)	1.424(2)	108.7(2)	124.3(2)	−155.4(2)	83.1(2)	71.7(1 )
molecule 2	1.527(2)	1.430(2)	1.356(2)	1.412(2)	108.4(2)	123.8(2)	145.8(2)	−91.5(2)	68.9(1)
**2**	1.525(2)	1.444(2)	1.369(2)	1.409(2)	106.8(1)	121.8(1)	157.2(1)	−82.8(1)	67.4(1)
**3a**									
molecule 1	1.534(3)	1.442(2)	1.362(2)	1.413(2)	108.9(2)	121.5(2)	173.1(2)	−66.3(2)	88.5(1)
molecule 2	1.523(3)	1.444(2)	1.362(2)	1.414(2)	108.4(1)	122.7(2)	158.6(2)	−81.7(2)	85.2(1)
**3b**	1.521(3)	1.440(3)	1.347(3)	1.406(3)	109.4(2)	125.0(1)	152.9(2)	−87.7(3)	68.2(1)
**4**	1.523(2)	1.445(2)	1.368(2)	1.414(2)	108.5(1)	121.9(1)	150.7(1)	−88.4(1)	74.3(1)
**6**	1.524(3)	1.440(3)	1.375(3)	1.426(3)	108.5(2)	122.6(2)	154.6(2)	−85.0(3)	69.5(1)
**10**	1.520(1)	1.448(1)	1.370(1)	1.409(1)	107.9(1)	123.2(2)	151.7(1)	−87.7(1)	73.2(1)

In the crystal structures of hemiaminals with a triazole ring [19,20] the nitrogen atom connected to the tetrahedral carbon atom also possesses a stable *sp^3^* hybridisation. In the currently presented class of hemiaminals the geometrical parameters are not in agreement with such hybridisation. The values of the N2P-C2P bond lengths are closer to the value of a formal C(*sp^2^*)-N aromatic bond (1.34 Å) than to a C(*sp^2^*)-N single bond (1.40 Å) [26]. The values of C12-N2P-C3P valence angles, close to 120°, also suggest that the nitrogen N2P atom is in a *sp^2^* rather than in a *sp^3^* hybridisation. Such an effect can be attributed to the conjugation between the aromatic ring and a nitrogen atom conncted with it.

**Figure 3:**
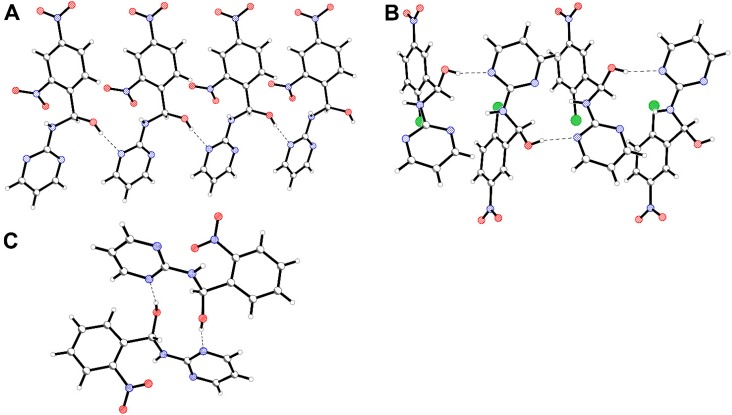
Hydrogen O-H∙∙∙N1_pyrimidine_ type bonding schemes in the crystal structures of hemiaminals, (**A**) an infinite chain of the same enantiomers, (**B**) an infinite chains of the opposite enantiomers, (**C**) dimers.

A wide range of intermolecular interactions such as O-H∙∙∙N, N-H∙∙∙N/O hydrogen bonds, C-H∙∙∙O/N weaker interactions and π-π stacking are observed in the crystal structures of presented hemiaminals. It seems that one of the above interactions (that is the O-H∙∙∙N1_pyrimidine_ type hydrogen bond) is present in the majority of the crystal structures of these hemiaminals. This type of hydrogen bonds links (A) molecules with the same configuration into infinite *-R-R-R-* chains (and due to centrosymmetric space groups *-S-S-S-* chains); (B) molecules with the opposite configuration into the infinite *-R-S-R-* chains or (C) dimers (Figure 3).

## 3. Experimental Section

### 3.1. General Information

All the syntheses were performed form commercially available compounds (Aldrich, Poznań, Poland) and solvents (POCh, Aldrich) without further purification. NMR spectra (^1^H: 600 MHz, ^13^C: 150.9 MHz) were measured on in DMSO-d_6_ solutions at room temperature (unless otherwise indicated) on an Avance III 600 MHz spectrometer (Bruker, Poznań, Poland). IR spectra were recorded in KBr pellets on Bruker 66/s FTIR and Bruker Vertex 70 FTIR spectrometers. Mass spectra were measured on Bruker Apex Ultra ESI-MS spectrometer. Elemental analyses were carried out on an Elemental analyser CHNS Vario EL III, Elementar Analysensystem GmbH (Hanau, Germany)

### 3.2. Crystallography

Single crystal X-Ray diffraction data (compounds **1**, **3a**, **4**, **6** and **10**) were collected on a Kuma KM4CCD four-circle diffractometer (Wrocław, Poland) equipped with Mo Kα radiation and a CCD camera (Sapphire) and an Xcalibur four-circle diffractometer (Wrocław, Poland) with Mo Kα radiation CCD camera (Ruby) for compounds **2** and **3b**. Measurements for all the compounds were carried out at 100 K using an Oxford Cryosystem adapter [27]. Programmes used for data collection and data reduction: CrysAlis CCD, Oxford Diffraction Ltd. (Oxford, UK); CrysAlis RED, Oxford Diffraction Ltd.; and CrysAlisPro, Agilent Technologies (Warsaw, Poland) [28]. Structures were solved by direct methods with the SHELXS [29] program and then refined by a full-matrix least squares method with the SHELXL97 [29] program with anisotropic thermal parameters for nonhydrogen atoms. Molecular graphics were prepared with the XP program [30]. Data for publication were prepared with the programs SHELXL97 [29] and PLATON [31]. CCDC 1409748-1409754 contain the supplementary crystallographic data for this paper. These data can be obtained free of charge from the CCDC, 12 Union Road, Cambridge CB2 1EZ, UK; Fax: +44 1223 336033; E-mail: deposit@ccdc.cam.ac.uk [32].

### 3.3. Synthetic Procedures

#### General Procedure for the Synthesis of Hemiaminals

Equimolar amounts of 2-aminopyrimidine and a suitable nitrobenzaldehyde derivative were dissolved in acetonitrile and stirred on a magnetic stirrer for two hours at room temperature (or slightly heated, up to 50 °C). Crystals obtained upon slow evaporation of the solvent were afterwards filtered off, washed with a small amount of acetonitrile and then diethyl ether and air-dried.

*(2-Nitrophenyl)(pyrimidin-2-ylamino)methanol* (**1**): Upon the reaction mixture was left standing for a few days at room temperature, colourless crystal blocks of (2-nitrophenyl)(pyrimidin-2-ylamino)methanol were deposited (58 mg, 93%), mp 99 °C. Calculated: C 53.66, H 4.09, N 22.75%; found: C 53.78, H 4.15, N 23.00%. MS: (*m/z*) 269.1 [M + Na]^+^. ^1^H-NMR δ: 8.35 (d, ^3^*J*_H6P/H6P,H5P_ = 4.8, 2H, H4P, H6P), 7.90–7.92 (m, 1H, H6), 7.82–7.85 (m, 2H, H3, H20), 7.69–7.72 (m, 1H, H5), 7.53–7.56 (m, 1H, H4), 7.03–7.06 (m, 1H, H12), 6.71 (t, ^3^*J*_H5P,H4P/H46_ = 4.8 Hz, 1H, H5P), 6.45 (d, ^3^*J*_H21,H12_ = 5.4 Hz, 1H, H21). ^13^C-NMR δ: 161.1 (C2P), 158.0 (C4P, C6P), 148.5 (C2), 136.2 (C1), 132.5 (C5), 128.8 (C6), 128.2 (C6), 123.7 (C3), 111.6 (C5P), 71.5 (C12). IR (KBr, cm^−1^): 3239 vs, 3114 vs, 3086 vs, 2714 m, 1968 vw, 1600 s, 1581 vs, 1528 vs, 1458 vs, 1410 vs, 1357 vs, 1255 vs, 1222 m, 1179 s, 1143 m, 1120 vs, 1103 s, 1090 s, 1080 vs, 1042 s, 1028 vs, 998 m, 986 m, 965 m, 920 m, 882 w, 857 m, 834 w, 802 vs, 789 vs, 743 s, 718 s, 687 m, 649 s, 606 s, 570 m, 534 s, 434 m, 403 m, 389 m. Crystal data: C_11_H_10_N_4_O_3_, *M* = 246.23, crystal system: monoclinic, space group: *P*2_1_/*c*, *a* = 16.321(5) Å, *b* = 9.583(4) Å, *c* = 14.482(6) Å, *β* = 103.57(3)°, *V* = 2201.8(15) Å^3^, *Z* = 8, crystal size: 0.39 × 0.21 × 0.19 mm, *ρ*_c_ = 1.486 g·cm^−^^3^, *μ* = 0.112 mm^−^^1^, *θ*_max_ = 30.00°, reflections: 21,083, independent: 6013, *R*_int_ = 0.0416, *R*_1_ = 0.0528, *wR*_2_ = 0.1382, GoF = 0.995.

*(2,4-Dinitrophenyl)(pyrimidin-2-ylamino)methanol* (**2**): After the reaction mixture was left standing for a few days at room temperature, yellow crystal blocks of compound **2** were deposited (51 mg, 88%), mp 108 °C. Calculated: C 45.37, H 3.11, N 24.05%; found: C 45.39, H 2.86, N 24.14%. MS: (*m/z*) 292.1 [M + H]^+^. ^1^H-NMR δ: 8.65 (d, ^4^*J*_H3,H5_ = 2.4 Hz, 1H, H3), 8.55 (dd, ^3^*J*_H5,H6_ = 9.0 Hz, ^4^*J*_H5,H3_ = 2.4 Hz, 1H, H5), 8.36 (d, ^3^*J*_H6P/H6P,H5P_ = 4.8, 2H, H4P, H6P), 8.18 (d, ^3^*J*_H6,H5_ = 9.0 Hz, 1H, H6), 8.05 (d, ^3^*J*_H20,H12_ = 9.0 Hz, 1H, H20), 7.07 (dd, ^3^*J*_H12,H20_ = 9.0 Hz, ^3^*J*_H12,H21_ = 3.6 Hz, 1H, H12), 6.81 (d, ^3^*J*_H21,H12_ = 3.6 Hz, 1H, H21), 6.75 (t, ^3^*J*_H5P,H4P/H46_ = 4.8 Hz, 1H, H5P). ^13^C-NMR δ: 161.0 (C2P), 158.0 (C4P, C6P), 148.3 (C3), 146.9 (C4), 142.8 (C1), 130.1 (C6), 126.8 (C5), 119.3 (C2), 112.0 (C5P), 71.3 (C12). IR (KBr, cm^−1^): 3343 s, 3100 m, 3026 m, 1611 m, 1589 s, 1574 vs, 1531 vs, 1511 vs, 1457 vs, 1421 m, 1343 s, 1298 m, 1246 s, 1197 w, 1116 m, 1090 w, 1073 m, 1049 s, 1032 s, 998 w, 953 vw, 926 vw, 848 m, 821 w, 807 m, 759 w, 736 w, 669 w, 645 w, 628 w, 585 m, 515 m. Crystal data: C_11_H_9_N_5_O_5_, *M* = 291.23, crystal system: triclinic, space group: *P*$\bar{1}$, *a* = 5.509(3) Å, *b* = 10.866(4) Å, *c* = 11.475(4) Å, *α* = 69.55(3)°, *β* = 89.43(3)°, *γ* = 80.60(3)°, *V* = 634.1(5) Å^3^, *Z* = 2, crystal size: 0.65 × 0.38 × 0.16 mm, *ρ*_c_ = 1.525 g·cm^−^^3^, *μ* = 0.124 mm^−^^1^, *θ*_max_ = 34.56°, reflections: 44,906, independent: 5244, *R*_int_ = 0.0233, *R*_1_ = 0.0476, *wR*_2_ = 0.1417, GoF = 1.040.

*(2-Chloro-5-nitrophenyl)(pyrimidin-2-ylamino)methanol* (**3a** and **3b**): After the reaction mixture was left standing for a few days at room temperature, two kinds of polymorphic crystals of (2-chloro-5-nitrophenyl)(pyrimidin-2-ylamino)methanol were deposited as colourless crystal needles (**3a**) and blocks (**3b**) (54 mg, 92%), mp 93–94 °C. Calculated: C 47.07, H 3.23, N 19.96%; found: C 46.92, H 3.33, N 20.18%. MS: (*m/z*) 303.0 [M + Na]^+^. ^1^H-NMR δ: 8.63 (d, ^4^*J*_H6,H4_ = 2.4 Hz, 1H, H6), 8.38 (d, ^3^*J*_H4P/H6P,H5P_ = 4.8 Hz, 2H, H4P, H6P), 8.18 (dd, ^3^*J*_H4,H3_ = 8.4 Hz, ^4^*J*_H4,H6_ = 2.4 Hz, 1H, H4), 7.95 (d, ^3^*J*_H20,H12_ = 9.0 Hz, 1H, H20), 7.73 (d, ^3^*J*_H3,H4_ = 8.4 Hz, 1H, H3), 6.83 (dd, ^3^*J*_H12,H20_ = 9.0 Hz, ^3^*J*_H12,H21_ = 4.2 Hz, 1H, H12), 6.72 (t, ^3^*J*_H5P,H4P/H6P_ = 4.8 Hz, 1H. H5P), 6.58 (d, ^3^*J*_H21,H12_ = 4.2 Hz, 1H, H21). ^13^C-NMR δ: 161.1 (C2P), 158.1 (C4P, C6P), 146.3 (C5), 142.0 (C1), 138.6 (C2), 130.6 (C3), 123.9 (C4), 123.2 (C6), 111.6 (C5P), 72.1 (C12). IR (KBr, cm^−1^): 3228 s, 3109 s, 3022 m, 2361 vw, 1919 vw, 1845 vw, 1792 vw, 1700 vw, 1590 vs, 1526 vs, 1464 vs, 1422 s, 1347 vs, 1310 s, 1253 s, 1240 s, 1189 m, 1138 m, 1110 s, 1102 s, 1068 s, 1034 vs, 1000 w, 979 vw, 964 w, 948 w, 925 w, 897 w, 838 m, 832 m, 822 m, 802 s, 788 m, 742 s, 668 w, 646 m, 619 s, 565 w, 529 w, 485 m. Crystal data (**3a**): C_11_H_9_ClN_4_O_3_, *M* = 280.67, crystal system: triclinic, space group: *P*$\bar{1}$, *a* = 9.591(4) Å, *b* = 10.906(4) Å, *c* = 13.140(5) Å, *α* = 73.54(3)°, *β* = 88.56(3)°, *γ* = 66.71(3)°, *V* = 1204.8(8) Å^3^, *Z* = 4, crystal size: 0.38 × 0.13 × 0.07 mm, *ρ*_c_ = 1.547 g℘cm^−^^3^, *μ* = 0.327 mm^−^^1^, *θ*_max_ = 29.99°, reflections: 11,880, independent: 5848, *R*_int_ = 0.0448, *R*_1_ = 0.0433, *wR*_2_ = 0.0639, GoF = 0.990; (**3b**): C_11_H_9_ClN_4_O_3_, *M* = 280.67, crystal system: monoclinic, space group: *P*2_1_/c, *a* = 14.841(4) Å, *b* = 9.391(3) Å, *c* = 9.029(3) Å, *β* = 101.91(3)°, *V* = 1231.3(7) Å^3^, *Z* = 4, crystal size: 0.25 × 0.10 × 0.06 mm, *ρ*_c_ = 1.514 g·cm^−^^3^, *μ* = 0.320 mm^−1^, *θ*_max_ = 28.77°, reflections: 5336, independent: 2805, *R*_int_ = 0.0224, *R*_1_ = 0.0539, *wR*_2_ = 0.1264, GoF = 0.999.

*(5-Chloro-2-nitrophenyl)(pyrimidin-2-ylamino)methanol* (**4**): After the reaction mixture was left standing for a few days at room temperature, colourless crystal blocks of compound **4** were deposited (53 mg, 85%), mp 114–115 °C. Calculated: C 47.07, H 3.23, N 19.96%; found: C 46.87, H 3.18, N 20.15%. MS: (*m/z*) 303.0 [M + Na]^+^. ^1^H-NMR δ: 8.35 (d, ^3^*J*_H4P/H6P,H5P_ = 4.8 Hz, 2H, H4P, H6P), 7.92–7.95 (m, 3H, H3,H6,H20), 7.64 (dd, ^3^*J*_H4,H3_ = 8.4 Hz, ^4^*J*_H4,H6_ = 2.4 Hz, 1H, H4), 7.02–7.05 (m, 1H, H12), 6.73 (t, ^3^*J*_H5P,H4P/H6P_ = 4.8 Hz, 1H. H5P), 6.61 (d, ^3^*J*_H21,H12_ = 4.8 Hz, 1H, H21). ^13^C-NMR δ: 161.1 (C2P), 158.0 (C4P, C6P), 146.8 (C2), 138.8 (C1), 137.3 (C5), 128.7 (C4), 128.2 and 126.1 (C3, C6), 111.9 (C5P), 71.0 (C12). IR (KBr, cm^−1^): 3319 s, 3101 m, 3061 m, 2959 m, 2833 m, 2703 m, 1653 w, 1589 vs, 1573 vs, 1520 vs, 1463 vs, 1423 s, 1398 m, 1367 vs, 1299 m, 1252 s, 1225 w, 1195 m, 1146 w, 1114 m, 1104 m, 1084 s, 1070 s, 1032 s, 1000 m, 953 vw, 935 w, 912 vw, 888 w, 845 m, 828 m, 803 m, 758 m, 693 w, 648 m, 601 w, 568 m, 518 w, 440 w. Crystal data: C_11_H_9_ClN_4_O_3_, *M* = 280.67, crystal system: monoclinic, space group: *P*2_1_/*n*, *a* = 11.353(4) Å, *b* = 5.441(2) Å, *c* = 19.095(6) Å, *β* = 90.29(3)°, *V* = 1179.5(7) Å^3^, *Z* = 4, crystal size: 0.36 × 0.26 × 0.24 mm, *ρ*_c_ = 1.581 g·cm^−^^3^, *μ* = 0.334 mm^−^^1^, *θ*_max_ = 36.91°, reflections: 20,358, independent: 5665, *R*_int_ = 0.0358, *R*_1_ = 0.0465, *wR*_2_ = 0.1201, GoF = 0.950.

*(4-Chloro-3-nitrophenyl)(pyrimidin-2-ylamino)methanol* (**5**): After the reaction mixture was left standing for a few days at room temperature, compound **5** was deposited in non-crystalline state (54 mg, 87%), mp 98–99 °C. Calculated: C 47.07, H 3.23, N 19.96%; found: C 46.79, H 3.34, N 20.23%. MS: (*m/z*) 303.0 [M + Na]^+^. ^1^H-NMR δ: 8.37 (d, ^3^*J*_H4P/H6P,H5P_ = 4.8 Hz, 2H, H4P,H6P), 8.13 (d, ^4^*J*_H2,H6_ = 1.8 Hz, 1H, H2), 7.94 (d, ^3^*J*_H20,H12_ = 9.0 Hz, 1H, H20), 7.77 (m, 2H, H5, H6), 6.72 (t, ^3^*J*_H5P,H4P/H6P_ = 4.8 Hz, 1H. H5P), 6.62 (dd, ^3^*J*_H12,H20_ = 9.0 Hz, ^3^*J*_H12,H21_ = 4.8 Hz, 1H, H12), 6.49 (d, ^3^*J*_H21,H12_ = 4.7 Hz, 1H, H21). ^13^C-NMR δ: 161.2 (C2P), 158.0 (C4P, C6P), 147.2 (C4), 144.0 (C1), 131.9 (C6), 131.3 (C5), 123.7 (C3), 123.4 (C2), 111.6 (C5P), 73.9 (C12). IR (KBr, cm^−1^): 3343 s, 3100 m, 3026 m, 1611 m, 1589 s, 1574 vs, 1531 vs, 1511 vs, 1457 vs, 1421 m, 1343 s, 1298 m, 1246 s, 1197 w, 1116 m, 1090 w, 1073 m, 1049 s, 1032 s, 998 w, 953 vw, 926 vw, 848 m, 821 w, 807 m, 759 w, 736 w, 669 w, 645 w, 628 w, 585 m, 515 m.

*(5-Fluoro-2-nitrophenyl)(pyrimidin-2-ylamino)methanol* (**6**): After the reaction mixture was left standing for a few days at room temperature, colourless crystal needles of compound **6** were deposited (60 mg, 93%), mp 116–117°C. Calculated: C 50.00, H 3.43, N 21.20%; found: C 50.15, H 3.53, N 21.34%. MS: (*m/z*) 265.1 [M + H]^+^. ^1^H-NMR (298 K) δ: 8.36 (d, ^3^*J*_H4P/H6P,H5P_ = 4.8, 2H, H4P, H6P), 7.99–8.02 (m, 1H, H3), 7.91 (d, ^3^*J*_H20,H12_ = 9.0 Hz, 1H, H_2_0), 7.68–7.70 (m, 1H, H6), 7.39–7.42 (m, 1H, H4), 7.05–7.08 (dd, ^3^*J*_H12,H20_ = 9.0 Hz, ^3^*J*_H12,H21_ = 5.4 Hz, 1H, H12), 6.73 (t, ^3^*J*_H5P,H4P/H6P_ = 4.8 Hz, 1H, H5P), 6.59 (d, ^3^*J*_H21,H12_ = 5.4 Hz, 1H, H21). ^13^C-NMR (298 K) δ: 163.6 (d, ^1^*J*_C5,F_ = 250.3 Hz, C5), 161.1 (C2P), 158.0 (C4P, C6P), 144.5 (d, ^4^*J*_C2,F_ = 3.0 Hz, C2), 140.5 (d, ^3^*J*_C1,F_ = 7.5 Hz, C1), 127.2 (d, ^3^*J*_C3,F_ = 9.0 Hz, C3), 115.1–115.6 (m, C4, C6), 111.8 (C5P), 71.1 (C12). IR (KBr, cm^−1^): 3322 m, 3243 w, 3064 m, 2834 w, 2697 w, 2364vw, 1885 vw, 1695 vw, 1622 m, 1591 vs, 1577 vs, 1521 vs, 1465 s, 1416 s, 1368 vs, 1307 w, 1295 w, 1265 s, 1252 s, 1227 m, 1187 vw, 1151 m, 1131 w, 1115 w, 1086 m, 1071 s, 1033 m, 1001 w, 965 w, 944 vw, 916 vw, 895 w, 847 w, 827 w, 811 m, 781 w, 750 vw, 713 vw, 697 w, 649 w, 630 m, 613 w, 584 w, 572 w, 544 vw, 512 vw, 496 vw, 444 w, 426 vw, 410 vw. Crystal data: C_11_H_9_FN_4_O_4_, *M* = 264.22, crystal system: monoclinic, space group: *P*2_1_/*n*, *a* = 11.182(3) Å, *b* = 5.503(2) Å, *c* = 18.942(5) Å, *β* = 90.50(3)°, *V* = 1165.5(6) Å^3^, *Z* = 4, crystal size: 0.58 × 0.29 × 0.19 mm, *ρ*_c_ = 1.506 g·cm^−^^3^, *μ* = 0.123 mm^−1^, *θ*_max_ = 29.99°, reflections: 8271, independent: 2855, *R*_int_ = 0.0890, *R*_1_ = 0.0568, *wR*_2_ = 0.0994, GoF = 0.998.

*(2-Fluoro-4-nitrophenyl)(pyrimidin-2-ylamino)methanol* (**7**): After the reaction mixture was left standing for a few days at room temperature, colourless crystal blocks of compound **7** were deposited (57 mg, 92%), mp 105 °C. Calculated: C 50.00, H 3.43, N 21.20%; found: C 49.97, H 3.47, N 21.35%. MS: (*m/z*) 265.1 [M + H]^+^. ^1^H-NMR δ: 8.36 (d, ^3^*J*_H4P/H6P,H5P_ = 4.8, 2H, H4P, H6P), 8.12–8.14 (m, 1H, H5), 8.05–8.07 (m, 1H, H3), 7.99–8.01 (m, 2H, H6, H20), 6.86–6.88 (m, 1H, H12), 6.72 (t, ^3^*J*_H5P,H4P/H6P_ = 4.8 Hz, 1H, H5P), 6.52 (d, ^3^*J*_H21,H12_ = 4.8 Hz, 1H, H21). ^13^C-NMR δ: 160.9 (C2P), 158.6 (d, ^1^*J*_C2,F_ = 250.5 Hz, C2), 158.1 (C4P, C6P), 147.7 (d, ^3^*J*_C4,F_ = 9.0 Hz, C4), 137.2 (d, ^2^*J*_C1,F_ = 13.6 Hz, C1), 129.2 (d, ^3^*J*_C6,F_ = 4.5 Hz, C6), 119.3 (d, ^4^*J*_C5,F_ = 3.0 Hz, C5), 111.6 (C5P), 110.9 (d, ^2^*J*_C3,F_ = 27.2 Hz, C3), 69.6 (C12). IR (KBr, cm^−1^): 3387 m, 3265 w, 3116 w, 3069 w, 3045 w, 2909 vw, 2360 vw, 1947 vw, 1588 vs, 1573 vs, 1522 vs, 1490 m, 1452 vs, 1432 m, 1416 s, 1383 w, 1356 vs, 1335 s, 1250 m, 1231 m, 1196 w, 1178 m, 1113 m, 1090 w, 1070 m, 1042 s, 992 vw, 976 vw, 943 w, 884 m, 846 vw, 819 m, 809 s, 804 s, 743 s, 722 vw, 677 vw, 633 w, 585 vw, 538 vw, 511 w, 493 vw, 464 vw, 408 vw.

*(4-Fluoro-2-nitrophenyl)(pyrimidin-2-ylamino)methanol* (**8**): After the reaction mixture was left standing for a few days at room temperature compound **8** was deposited in non-crystalline state (62 mg, 89%), mp 90 °C. Calculated: C 50.00, H 3.43, N 21.20%; found: C 49.81, H 3.20, N 21.30%. MS: (*m/z*) 265.1 [M + H]^+^. ^1^H-NMR (298 K) δ: 8.35 (d, ^3^*J*_H4P/H6P,H5P_ = 4.8, 2H, H4P, H6P), 7.93–7.95 (m, 1H, H6), 7.88 (d, ^3^*J*_H20,H12_ = 9.0 Hz, 1H, H20), 7.82–7.84 (m, 1H, H3), 7.59–7.62 (m, 1H, H5), 6.97–7.00 (dd, ^3^*J*_H12,H20_ = 9.0 Hz, ^3^*J*_H12,H21_ = 5.4 Hz, 1H, H12), 6.72 (t, ^3^*J*_H5P,H4P/H6P_ = 4.8 Hz, 1H, H5P), 6.52 (d, ^3^*J*_H21,H12_ = 5.4 Hz, 1H, H21). ^13^C-NMR (298 K) δ: 160.7 (d, ^1^*J*_C4,F_ = 247.3 Hz, C4), 161.1 (C2P), 158.0 (C4P, C6P), 148.8 (d, ^3^*J*_C2,F_ = 9.0 Hz, C2), 132.6 (d, ^4^*J*_C1,F_ = 4.5 Hz, C1), 130.4 (d, ^3^*J*_C6,F_ = 7.5 Hz, C6), 119.4 (d, ^2^*J*_C5,F_ = 21.1 Hz, C5), 111.7 (C5P), 111.4 (d, ^2^*J*_C3,F_ = 27.1 Hz, C3), 71.2 (C12). IR (KBr, cm^−1^): 3314 s, 3087 s, 2849 m, 3693 w, 2361 vw, 1971 vw, 1936 vw, 1615 s, 1578 vs, 1529 vs, 1491 vs, 1465 vs, 1425 vs, 1369 vs, 1298 m, 1277 m, 1260 s, 1253 s, 1228 vs, 1185 w, 1130 w, 1114 s, 1090 s, 1066 vs, 1037 vs, 998 m, 970 w, 942 s, 913 vw, 877 m, 845 vs, 823 s, 811 vs, 800 s, 746 m, 675 m, 648 w, 628 w, 584 vs, 551 m, 530 m, 454 vw, 428 w.

*(4-Fluoro-3-nitrophenyl)(pyrimidin-2-ylamino)methanol* (**9**): After the reaction mixture was left standing for a few days at room temperature compound **9** was deposited in non-crystalline state (55 mg, 90%), mp 116–117 °C. Calculated: C 50.00, H 3.43, N 21.20%; found: C 49.84, H 3.53, N 21.08%. MS: (*m/z*) 265.1 [M + H]^+^. ^1^H-NMR (298 K) δ: 8.37 (d, ^3^*J*_H4P/H6P,H5P_ = 4.8, 2H, H4P, H6P), 8.24 (dd, ^4^*J*_H2,F_ = 7.5 Hz, ^4^*J*_H2,H6_ = 2.1 Hz, 1H, H2), 7.94 (d, ^3^*J*_H20,H12_ = 9.0 Hz, 1H, H20), 7.87–7.90 (m, 1H, H6), 7.55–7.59 (m, 1H, H5), 6.72 (t, ^3^*J*_H5P,H4P/H6P_ = 4.8 Hz, 1H, H5P), 6.63 (dd, ^3^*J*_H12,H20_ = 9.0 Hz, ^3^*J*_H12,H21_ = 4.8 Hz, 1H, H12), 6.47 (d, ^3^*J*_H21,H12_ = 4.8 Hz, 1H, H21). ^13^C-NMR (298 K) δ: 161.2 (C2P), 158.0 (C4P, C6P), 153.9 (d, ^1^*J*_C4,F_ = 259.4 Hz, C4), 140.4 (d, ^4^*J*_C1,F_ = 4.5 Hz, C1), 136.3 (d, ^2^*J*_C3,F_ = 6.0 Hz, C3), 134.3 (d, ^3^*J*_C6,F_ = 9.0 Hz, C6), 123.7 (d, ^3^*J*_C2,F_ = 3.0 Hz, C2), 118.1 (d, ^2^*J*_C5,F_ = 19.6 Hz, C5), 111.6 (C5P), 73.9 (C12). IR (KBr, cm^−1^): 3329 vs, 3079 s, 3041 s, 2830 m, 2686 m, 2349 vw, 1965 vw, 1621 s, 1590 vs, 1572 vs, 1537 vs, 1519 vs, 1461 vs, 1424 s, 1349 vs, 1309 m, 1286 m, 1269 m, 1252 vs, 1238 s, 1199 w, 1181 w, 1150 w, 1117 s, 1092 s, 1086 s, 1071 s, 1027 vs, 997 m, 954 w, 926 w, 900 vw, 856 m, 824 m, 801 m, 761 w, 710 w, 673 m, 635 w, 597 m, 575 s, 530 w, 508 w, 473 vw, 393 vw.

*[2-Nitro-4-(trifluoromethyl)phenyl](pyrimidin-2-ylamino)methanol* (**10**): After the reaction mixture was left standing for a few days at room temperature, pale yellow crystal needles of compound **10**, C_12_H_9_F_3_N_4_O_3_, were deposited (45 mg, 87%), mp 105 °C. Calculated: C 45.87, H 2.89, N 17.83%; found: C 46.07, H 2.90, N 18.03%. MS: (*m/z*) 315.1 [M + H]^+^. ^1^H-NMR (298 K) δ: 8.36 (d, ^3^*J*_H4P/H6P,H5P_ = 4.8, 2H, H4, H6), 8.26 (s, 1H, H3), 8.11–8.15 (m, 2H, H5, H6), 7.98 (d, ^3^*J*_H20,H12_ = 9.0 Hz, 1H, H20), 7.06 (dd, ^3^*J*_H12,H20_ = 9.0 Hz, ^3^*J*_H12,H21_ = 4.8 Hz, 1H, H12), 6.74 (t, ^3^*J*_H5P,H4P/H6P_ = 4.8 Hz, 1H, H5P), 6.70 (d, ^3^*J*_H21,H12_ = 4.8 Hz, 1H, H21). ^13^C-NMR (298 K) δ: 161.1 (C2P), 158.0 (C4P, C6P), 148.6 (C2), 140.5 (C1), 129.7 (C6), 129.2 (q, ^2^*J*_C4,F_ = 33.2 Hz, C4), 129.0 (q, ^3^*J*_C5,F_ = 4.5 Hz, C5), 123.1 (q, ^1^*J*_C7,F_ = 272.9 Hz, C7), 121.0 (q, ^3^*J*_C3,F_ = 3.0 Hz, C3), 111.9 (C5P), 71.2 (C12). IR (KBr, cm^−^^1^): 3413 s, 3106 m, 3057 m, 1632 w, 1591 vs, 1578 vs, 1536v s, 1504 s, 1461 s, 1410 m, 1367 m, 1325 vs, 1308 m, 1286 w, 1244 m, 1201 w, 1182 s, 1151 m, 1127 s, 1091 m, 1077 s, 1066 m, 1043 s, 998 vw, 932 vw, 915 vw, 894 vw, 852 m, 830 w, 807 w, 784 w, 731 vw, 702 w, 675 vw, 649 w, 629 vw, 578 w, 517 w, 495 w, 458 w, 439 vw, 415 vw. Crystal data: C_12_H_9_F_3_N_4_O_3_, *M* = 314.23, crystal system: monoclinic, space group: *C*2/*c*, *a* = 38.572(9) Å, *b* = 5.345(3) Å, *c* = 13.358(4) Å, *β* = 110.14(3)°, *V* = 2585.6(18) Å^3^, *Z* = 8, crystal size: 0.70 × 0.20 × 0.12 mm, *ρ*_c_ = 1.614 g·cm^−^^3^, *μ* = 0.147 mm^−1^, *θ*_max_ = 36.97°, reflections: 23,733, independent: 5929, *R*_int_ = 0.0305, *R*_1_ = 0.0464, *wR*_2_ = 0.1262, GoF = 0.999.

## 4. Conclusions

We have discovered a new class of stable hemiaminal derivatives of 2-aminopyrimidine and nitrobenzaldehydes. Their stability can be attributed to the presence of both electron-withdrawing nitro groups on the phenyl ring and a pyrimidine ring. These compounds do not need extra stabilisation.

## Supplementary Files

Supplementary File 1
