# Supplementary material for: Stable Hemiaminals: 2-Aminopyrimidine Derivatives
Source: Molecules. 2015 Aug 6;20(8):14365–76. doi: 10.3390/molecules200814365 (PMC6332198; doi:10.3390/molecules200814365)
Supplement: Supplementary File 1 [file molecules-20-14365-s001.pdf]

Supplementary Materials

A Table of Contents

1 General Remarks S2
2 X-Ray Crystallography S3
3 Synthetic Procedures and Characterisation of New Compounds S3
3.1 (2-Nitrophenyl)(pyrimidin-2-ylamino)methanol (1) S3
3.1.1 Synthesis S3
3.1.2 Elemental Analysis S3
3.1.3 Mass Spectrometry S4
3.1.4 NMR Spectroscopy S4
3.1.5 IR Spectroscopy S7
3.1.6 Crystallography S7
3.2 (2,4-Dinitrophenyl)(pyrimidin-2-ylamino)methanol (2) S16
3.2.1 Synthesis S16
3.2.2 Elemental Analysis S16
3.2.3 Mass Spectrometry S17
3.2.4 NMR Spectroscopy S17
3.2.5 IR Spectroscopy S20
3.2.6 Crystallography S20
3.3 (2-Chloro-5-nitrophenyl)(pyrimidin-2-ylamino)methanol (3a and 3b) S26
3.3.1 Synthesis S26
3.3.2 Elemental Analysis S26
3.3.3 Mass Spectrometry S26
3.3.4 NMR Spectroscopy S27
3.3.5 IR Spectroscopy S29
3.3.6 Crystallography S30
3.4 (5-Chloro-2-nitrophenyl)(pyrimidin-2-ylamino)methanol (4) S44
3.4.1 Synthesis S44
3.4.2 Elemental Analysis S45
3.4.3 Mass Spectrometry S45
3.4.4 NMR Spectroscopy S45
3.4.5 IR Spectroscopy S47
3.4.6 Crystallography S48
3.5 (4-Chloro-3-nitrophenyl)(pyrimidin-2-ylamino)methanol (5) S54
3.5.1 Synthesis S54
3.5.2 Elemental Analysis S54
3.5.3 Mass Spectrometry S54
3.5.4 NMR Spectroscopy S55
3.5.5 IR Spectroscopy S57
3.6 (5-Fluoro-2-nitrophenyl)(pyrimidin-2-ylamino)methanol (6) S57
3.6.1 Synthesis S57
3.6.2 Elemental Analysis S58

3.6.3 Mass Spectrometry ... S58
3.6.4 NMR Spectroscopy ... S58
3.6.5 IR Spectroscopy ... S60
3.6.6 Crystallography ... S61
3.7 (2-Fluoro-4-nitrophenyl)(pyrimidin-2-ylamino)methanol (7) ... S67
3.7.1 Synthesis ... S67
3.7.2 Elemental Analysis ... S67
3.7.3 Mass Spectrometry ... S67
3.7.4 NMR Spectroscopy ... S68
3.7.5 IR Spectroscopy ... S70
3.8 (4-Fluoro-2-nitrophenyl)(pyrimidin-2-ylamino)methanol (8) ... S71
3.8.1 Synthesis ... S71
3.8.2 Elemental Analysis ... S71
3.8.3 Mass Spectrometry ... S71
3.8.4 NMR Spectroscopy ... S72
3.8.5 IR Spectroscopy ... S74
3.9 (4-Fluoro-3-nitrophenyl)(pyrimidin-2-ylamino)methanol (9) ... S75
3.9.1 Synthesis ... S75
3.9.2 Elemental Analysis ... S75
3.9.3 Mass Spectrometry ... S75
3.9.4 NMR Spectroscopy ... S76
3.9.5 IR Spectroscopy ... S78
3.10 [2-Nitro-4-(trifluoromethyl)phenyl](pyrimidin-2-ylamino)methanol (10) ... S79
3.10.1 Synthesis ... S79
3.10.2 Elemental Analysis ... S79
3.10.3 Mass Spectrometry ... S79
3.10.4 NMR Spectroscopy ... S80
3.10.5 IR Spectroscopy ... S82
3.10.6 Crystallography ... S83
4 References ... S89

1. General Remarks

All the syntheses were performed form commercially available compounds (Aldrich) and solvents (POCh, Aldrich) without further purification. NMR spectra were measured on Bruker Avance III 600 MHz spectrometers. IR spectra were recorded in KBr pellets on Bruker 66/s FTIR and Bruker Vertex 70 FTIR spectrometers. Mass spectra were measured on Bruker Apex Ultra ESI-MS spectrometer. Elemental analysis were carried out on Elemental analyser CHNS Vario EL III, Elementar Analysensystem GmbH.

2. X-ray Crystallography

Single crystal X-ray diffraction data were collected at Kuma KM4CCD four-circle diffractometer with Mo Kα radiation and CCD camera (Sapphire), compounds compounds (**1**, **3a**, **4**, **6** and **10**) and Xcalibur four-circle diffractometer with Mo Kα radiation CCD camera (Ruby), compounds **2** and **3b**. Measurements for all the compounds were carried out at 100 K using an Oxford Cryosystem adapter [1]. Programmes used for data collection and data reduction: CrysAlis CCD, Oxford Diffraction Ltd.; CrysAlis RED, Oxford Diffraction Ltd.; and CrysAlisPro, Agilent Technologies [2]. Structures were solves by direct methods with SHELXS [3] program and then refined by a full-matrix least squares method with SHELXL97 [3] program with anisotropic thermal parameters for nonhydrogen atoms. Molecular graphics were prepared with the XP program [4]. Data for publication were prepared with the programs SHELXL97 [3], CIFTAB [3] and PLATON [5]. CCDC 1409748-1409754 contain the supplementary crystallographic data for this paper. These data can be obtained free of charge via http://www.ccdc.cam.ac.uk/conts/retrieving.html (or from the CCDC, 12 Union Road, Cambridge CB2 1EZ, UK; Fax: +44 1223 336033; E-mail: deposit@ccdc.cam.ac.uk).

3. Synthetic Procedures and Characterisation of New Compounds

3.1. (2-Nitrophenyl)(pyrimidin-2-ylamino)methanol (1)

3.1.1. Synthesis

Acetonitrilic solution (3 mL) of 2-nitrobenzaldehyde (39 mg) was added to an acetonitrilic solution (3 mL) of 2-aminopyrimidine (25 mg). The reaction mixture after complete dissolution was stirred for 2 h at 50 °C. The title compound crystallised directly from the mother liquor. Upon standing 2 days at the room temperature, the solution deposited colourless crystal blocks. The crystals were filtered off, washed with a small amount of acetonitrile and diethyl ether then dried in the air to afford (2-nitrophenyl)(pyrimidin-2-ylamino)methanol—(58 mg, 93%), mp 99 °C.

3.1.2. Elemental Analysis

	% C	% H	% N
Calculated	53.66	4.09	23.00
Found	53.78	4.15	22.77

3.1.3. Mass Spectrometry

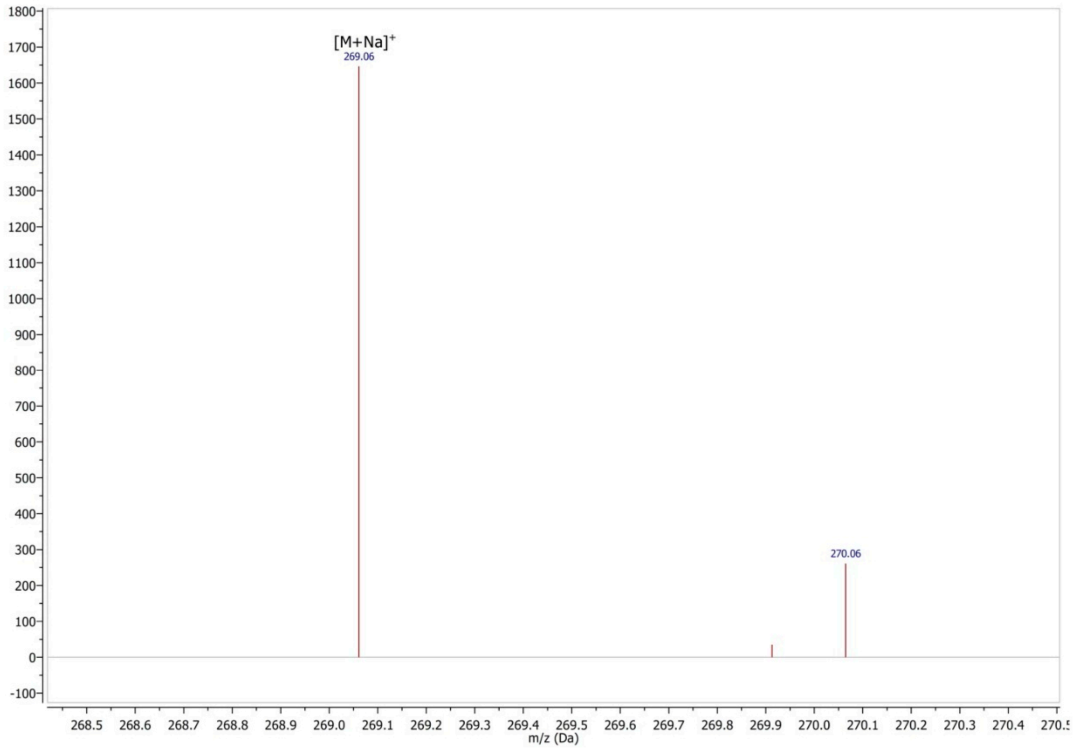

3.1.4. NMR Spectroscopy

^{1}H-NMR

^{1}H-NMR (600 MHz, DMSO, RT) δ: 8.35 (d, $^3J_{H6P/H6P,H5P}$ = 4.8, 2H, H4P,H6P), 7.90–7.92 (m, 1H, H6), 7.82–7.85 (m, 2H, H3,H20), 7.69–7.72 (m, 1H, H5), 7.53–7.56 (m, 1H, H4), 7.03–7.06 (m, 1H, H12), 6.71 (t, $^3J_{H5P,H4P/H46}$ = 4.8 Hz, 1H, H5P), 6.45 (d, $^3J_{H21,H12}$ = 5.4 Hz, 1H, H21).

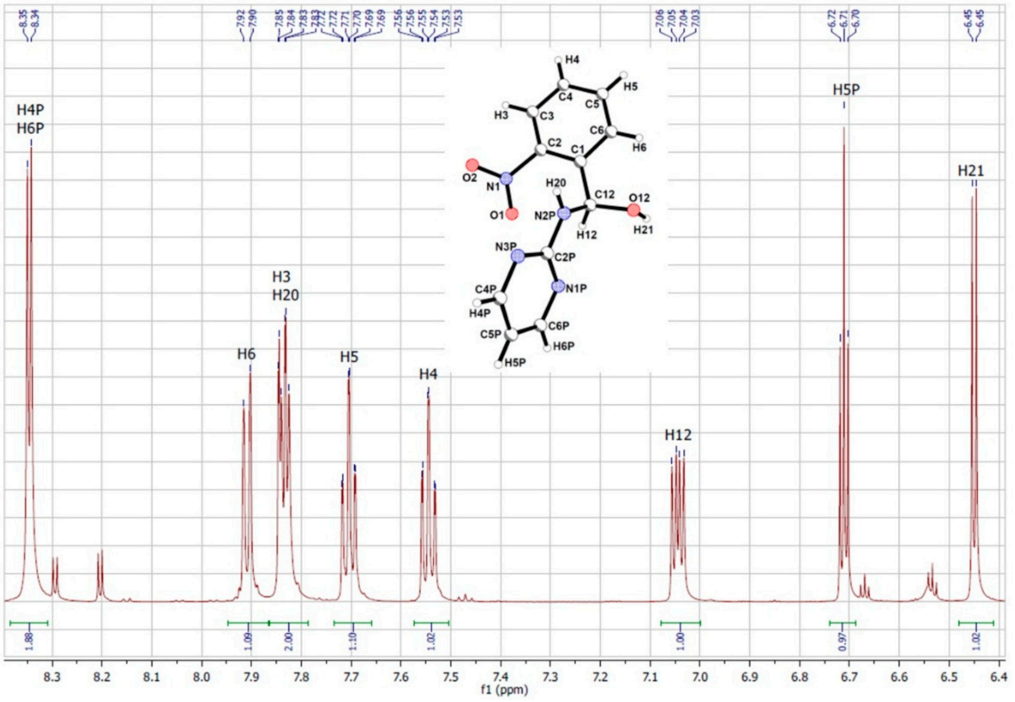

^{13}C-NMR

^{13}C-NMR (150.9 MHz, DMSO, RT) δ: 161.1 (C2P), 158.0 (C4P, C6P), 148.5 (C2), 136.2 (C1), 132.5 (C5), 128.8 (C6), 128.2 (C6), 123.7 (C3), 111.6 (C5P), 71.5 (C12).

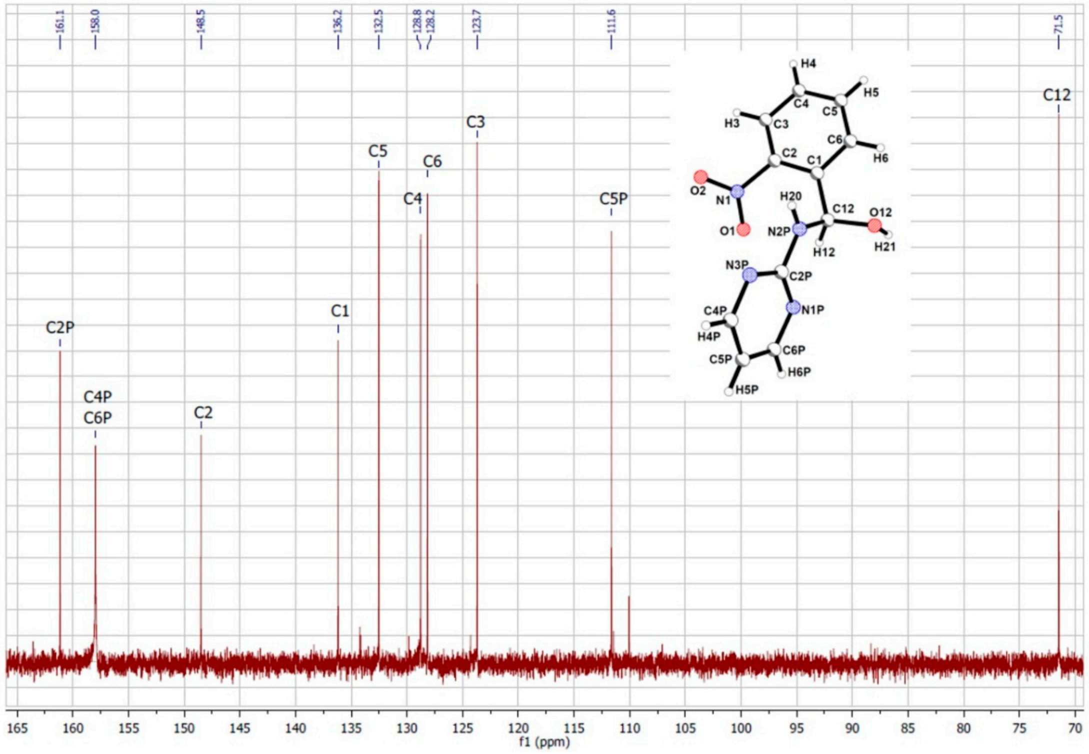

COSY

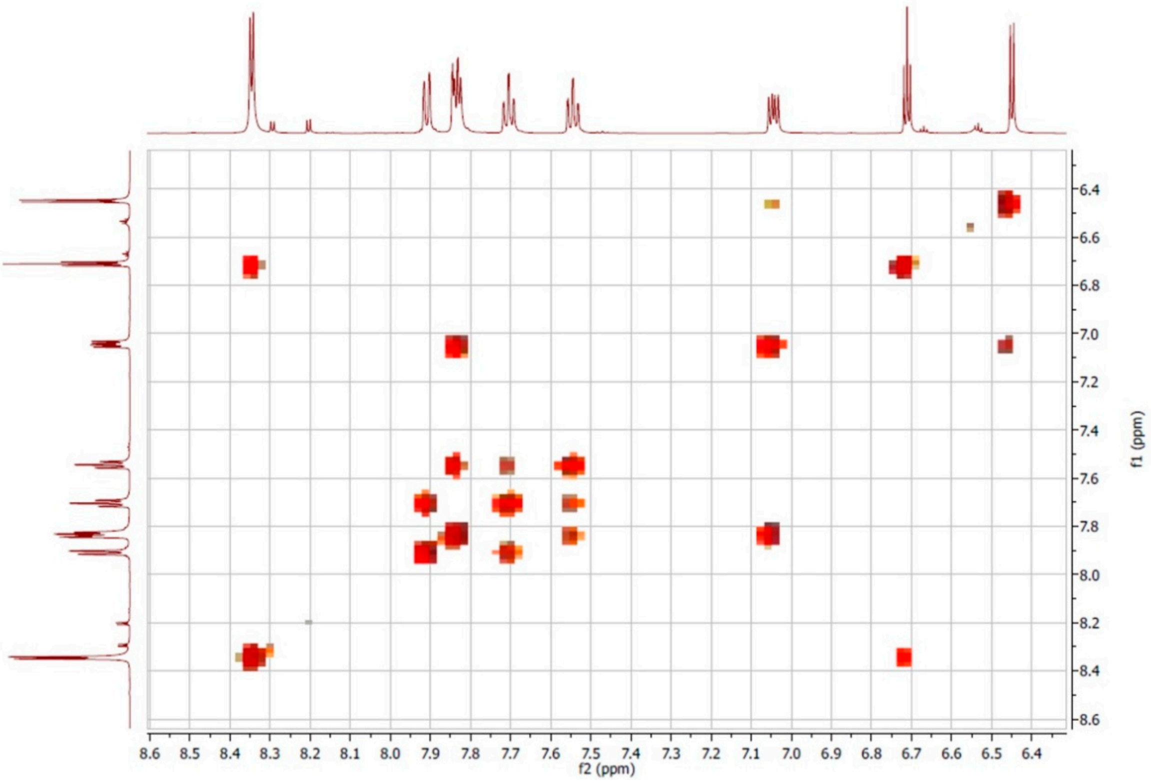

HMQC

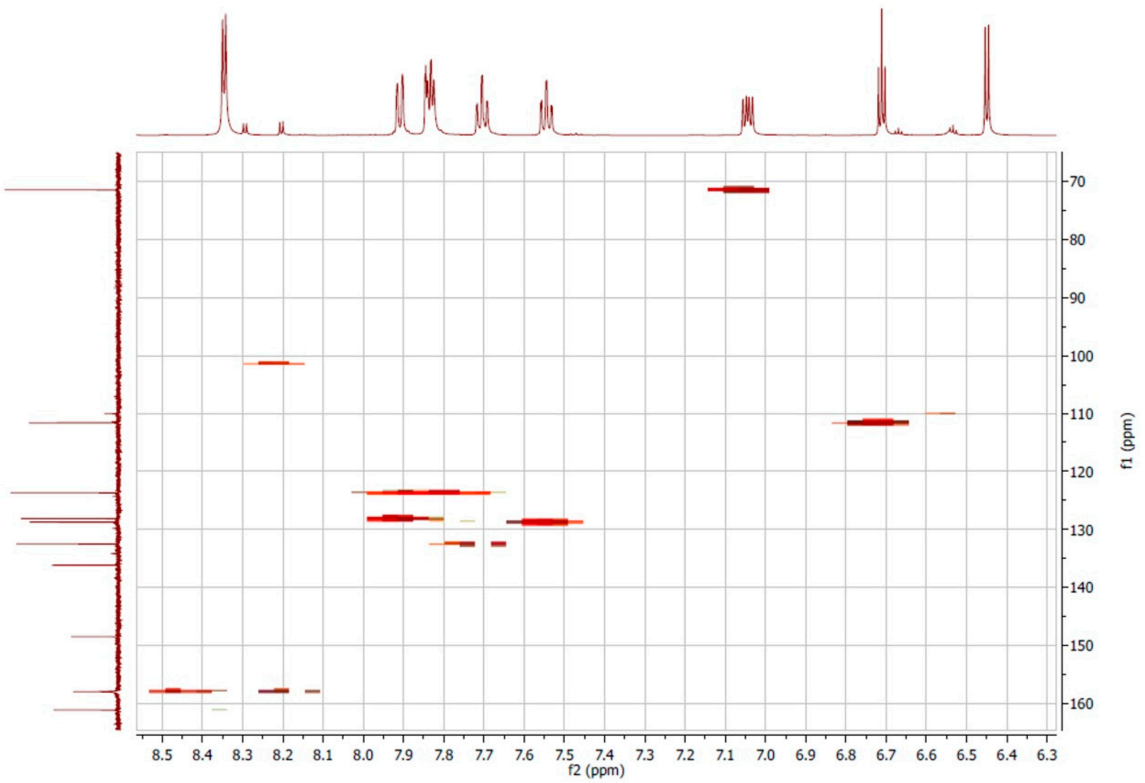

HMBC

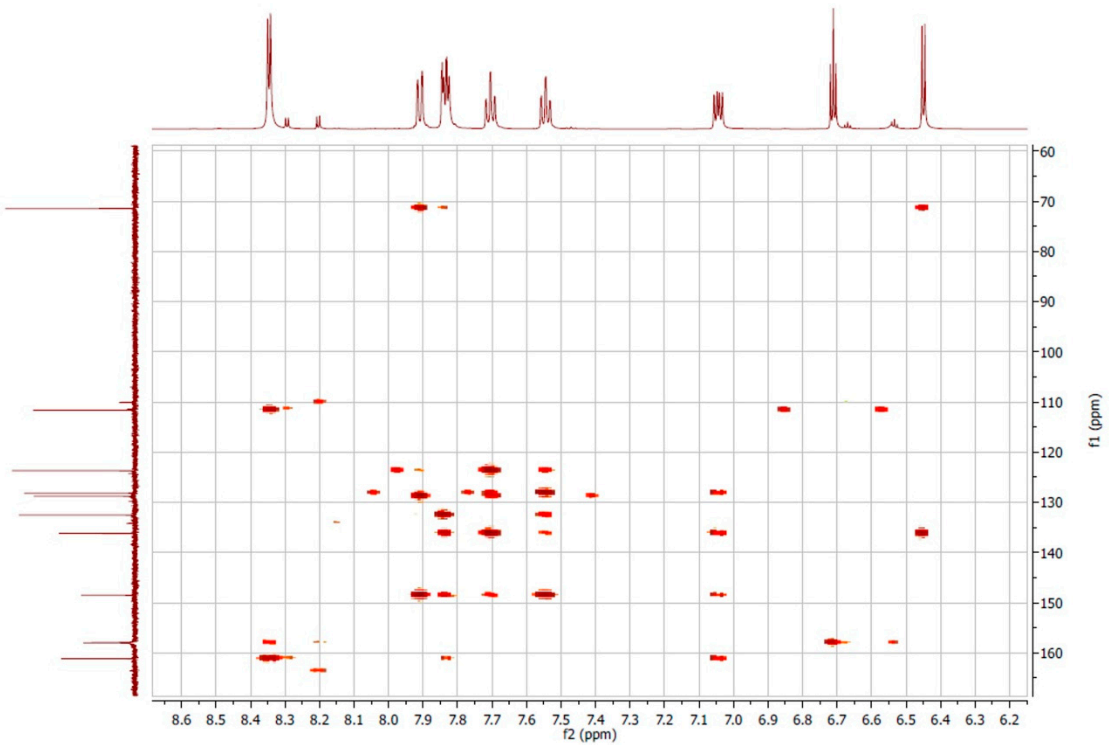

3.1.5. IR Spectroscopy

IR (KBr, cm^{-1}): 3239 vs, 3114 vs, 3086 vs, 2714 m, 1968 vw, 1600 vs, 1581 vs, 1528 vs, 1458 vs, 1410 vs, 1357 vs, 1255 vs, 1222 m, 1179 s, 1143 m, 1120 vs, 1103 s, 1090 s, 1080 vs, 1042 s, 1028 vs, 998 m, 986 m, 965 m, 920 m, 882 w, 857 m, 834 w, 802 vs, 789 vs, 743 s, 718 s, 687 m, 649 s, 606 s, 570 m, 534 s, 434 m, 403 m, 389 m.

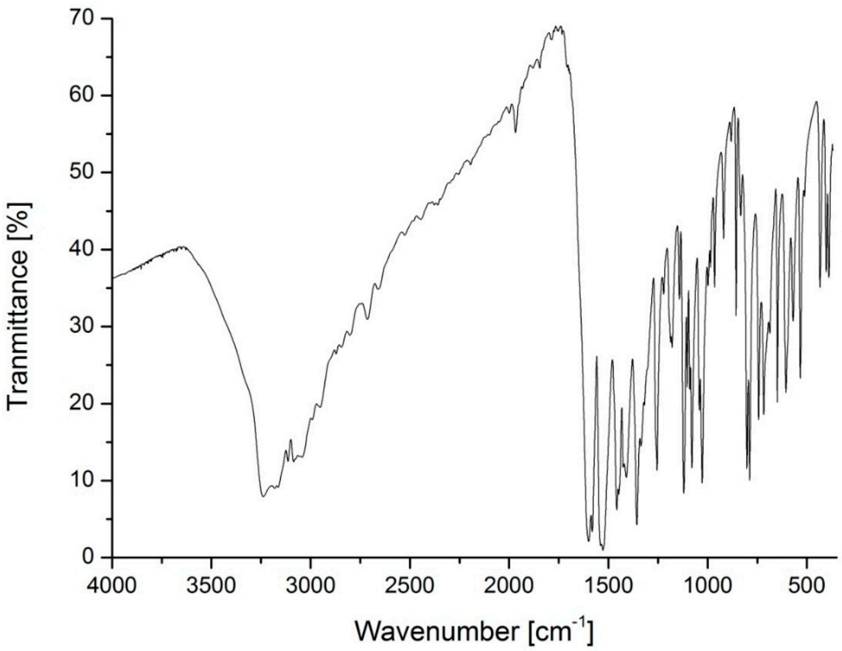

3.1.6. Crystallography

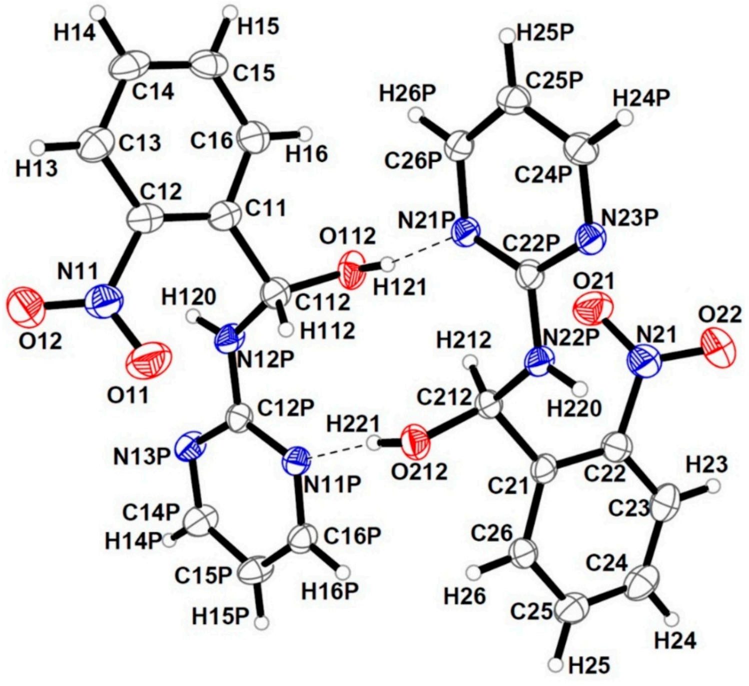

Figure S1. Molecular structure and labelling for (2-nitrophenyl)(pyrimidin-2-ylamino)methanol (1). Displacement ellipsoids are shown at the 50% probability level.

Table S3.1.6.1. Crystal data and structure refinement for 1.

Identification code	1	
Empirical formula	C11 H10 N4 O3	
Formula weight	246.23	
Temperature	100(2) K	
Wavelength	0.71073 Å	
Crystal system	Monoclinic	
Space group	P 1 21/c 1	
Unit cell dimensions	a = 16.321(5) Å	α = 90°
	b = 9.583(4) Å	β= 103.57(3)°
	c = 14.482(6) Å	γ = 90°
Volume	2201.8(15) $Å^3$	
Z	8	
Density (calculated)	1.486 Mg/m^3	
Absorption coefficient	0.112 mm^{-1}	
F(000)	1024	
Crystal size	0.39 × 0.21 × 0.19 mm^3	
Theta range for data collection	2.88 to 30.00°	
Index ranges	$-22 \leq h \leq 21$, $-11 \leq k \leq 12$, $-20 \leq l \leq 17$	
Reflections collected	21,083	
Independent reflections	6013 [R(int) = 0.0416]	
Completeness to theta = 27.00°	99.9%	
Absorption correction	Semi-empirical from equivalents	
Max. and min. transmission	1.00000 and 0.95853	
Refinement method	Full-matrix least-squares on F^2	
Data/restraints/parameters	6013/0/337	
Goodness-of-fit on F^2	0.995	
Final R indices [I>2sigma(I)]	R1 = 0.0528, wR2 = 0.1278	
R indices (all data)	R1 = 0.0937, wR2 = 0.1382	
Largest diff. peak and hole	0.384 and −0.214 e.$Å^{-3}$	

Table S3.1.6.2. Atomic coordinates (× 10^4) and equivalent isotropic displacement parameters ($Å^2$ × 10^3) for 1. U(eq) is defined as one third of the trace of the orthogonalized U^{ij} tensor.

	**x**	**y**	**z**	**U(eq)**
C(11)	3692(1)	5882(2)	1710(1)	26(1)
C(12)	4434(1)	6509(2)	2186(1)	29(1)
N(11)	4576(1)	6871(2)	3200(1)	34(1)
O(11)	4362(1)	6018(2)	3736(1)	48(1)
O(12)	4923(1)	7985(2)	3463(1)	43(1)
C(13)	5071(1)	6913(2)	1742(1)	33(1)
C(14)	4952(1)	6665(2)	777(1)	33(1)
C(15)	4213(1)	6062(2)	283(1)	31(1)
C(16)	3596(1)	5677(2)	742(1)	30(1)
C(112)	2978(1)	5469(2)	2185(1)	26(1)
O(112)	2322(1)	4759(1)	1534(1)	31(1)

Table S3.1.6.2. *Cont.*

	x	y	z	U(eq)
N(12P)	2646(1)	6721(2)	2523(1)	26(1)
N(11P)	2114(1)	5546(2)	3665(1)	24(1)
C(12P)	2245(1)	6750(2)	3244(1)	22(1)
N(13P)	2013(1)	8030(2)	3484(1)	27(1)
C(14P)	1601(1)	8067(2)	4177(1)	32(1)
C(15P)	1424(1)	6895(2)	4648(1)	30(1)
C(16P)	1705(1)	5654(2)	4364(1)	26(1)
C(21)	1196(1)	1560(2)	3201(1)	22(1)
C(22)	544(1)	833(2)	2603(1)	26(1)
N(21)	590(1)	471(2)	1627(1)	35(1)
O(21)	847(1)	1354(2)	1153(1)	42(1)
O(22)	350(1)	−679(2)	1335(1)	57(1)
C(23)	−173(1)	400(2)	2877(1)	32(1)
C(24)	−268(1)	749(2)	3769(1)	34(1)
C(25)	366(1)	1456(2)	4386(1)	31(1)
C(26)	1093(1)	1842(2)	4112(1)	26(1)
C(212)	1994(1)	1999(2)	2898(1)	22(1)
O(212)	2572(1)	2692(1)	3634(1)	28(1)
N(22P)	2380(1)	777(2)	2623(1)	24(1)
N(21P)	2900(1)	1962(1)	1484(1)	22(1)
C(22P)	2801(1)	754(2)	1920(1)	21(1)
N(23P)	3074(1)	−508(2)	1709(1)	24(1)
C(24P)	3489(1)	−529(2)	1013(1)	26(1)
C(25P)	3635(1)	646(2)	532(1)	25(1)
C(26P)	3320(1)	1870(2)	790(1)	24(1)

Table 3.1.6.3. Bond lengths [Å] and angles [°] for 1.

C(11)-C(12)	1.382(3)
C(11)-C(16)	1.387(2)
C(11)-C(112)	1.537(2)
C(12)-C(13)	1.400(3)
C(12)-N(11)	1.474(2)
N(11)-O(12)	1.227(2)
N(11)-O(11)	1.232(2)
C(13)-C(14)	1.386(3)
C(13)-H(13)	0.9500
C(14)-C(15)	1.378(3)
C(14)-H(14)	0.9500
C(15)-C(16)	1.381(3)
C(15)-H(15)	0.9500
C(16)-H(16)	0.9500
C(112)-O(112)	1.424(2)

Table S3.1.6.3. *Cont.*

C(112)-N(12P)	1.448(2)
C(112)-H(112)	1.0000
O(112)-H(121)	0.93(2)
N(12P)-C(12P)	1.358(2)
N(12P)-H(120)	0.86(2)
N(11P)-C(16P)	1.342(2)
N(11P)-C(12P)	1.345(2)
C(12P)-N(13P)	1.352(2)
N(13P)-C(14P)	1.333(2)
C(14P)-C(15P)	1.379(3)
C(14P)-H(14P)	0.9500
C(15P)-C(16P)	1.371(3)
C(15P)-H(15P)	0.9500
C(16P)-H(16P)	0.9500
C(21)-C(22)	1.392(2)
C(21)-C(26)	1.394(2)
C(21)-C(212)	1.527(2)
C(22)-C(23)	1.384(3)
C(22)-N(21)	1.474(2)
N(21)-O(22)	1.213(2)
N(21)-O(21)	1.224(2)
C(23)-C(24)	1.377(3)
C(23)-H(23)	0.9500
C(24)-C(25)	1.375(3)
C(24)-H(24)	0.9500
C(25)-C(26)	1.388(3)
C(25)-H(25)	0.9500
C(26)-H(26)	0.9500
C(212)-O(212)	1.412(2)
C(212)-N(22P)	1.430(2)
C(212)-H(212)	1.0000
O(212)-H(221)	0.92(2)
N(22P)-C(22P)	1.356(2)
N(22P)-H(220)	0.80(2)
N(21P)-C(22P)	1.346(2)
N(21P)-C(26P)	1.347(2)
C(22P)-N(23P)	1.348(2)
N(23P)-C(24P)	1.340(2)
C(24P)-C(25P)	1.374(2)
C(24P)-H(24P)	0.9500

Table S3.1.6.3. *Cont.*

C(25P)-C(26P)	1.367(2)
C(25P)-H(25P)	0.9500
C(26P)-H(26P)	0.9500
C(12)-C(11)-C(16)	116.31(17)
C(12)-C(11)-C(112)	123.75(16)
C(16)-C(11)-C(112)	119.89(17)
C(11)-C(12)-C(13)	123.35(17)
C(11)-C(12)-N(11)	120.94(16)
C(13)-C(12)-N(11)	115.62(17)
O(12)-N(11)-O(11)	124.06(17)
O(12)-N(11)-C(12)	117.85(16)
O(11)-N(11)-C(12)	118.05(17)
C(14)-C(13)-C(12)	118.22(19)
C(14)-C(13)-H(13)	120.9
C(12)-C(13)-H(13)	120.9
C(15)-C(14)-C(13)	119.60(17)
C(15)-C(14)-H(14)	120.2
C(13)-C(14)-H(14)	120.2
C(14)-C(15)-C(16)	120.69(17)
C(14)-C(15)-H(15)	119.7
C(16)-C(15)-H(15)	119.7
C(15)-C(16)-C(11)	121.82(19)
C(15)-C(16)-H(16)	119.1
C(11)-C(16)-H(16)	119.1
O(112)-C(112)-N(12P)	109.63(14)
O(112)-C(112)-C(11)	111.01(14)
N(12P)-C(112)-C(11)	108.73(15)
O(112)-C(112)-H(112)	109.1
N(12P)-C(112)-H(112)	109.1
C(11)-C(112)-H(112)	109.1
C(112)-O(112)-H(121)	102.7(14)
C(12P)-N(12P)-C(112)	124.31(15)
C(12P)-N(12P)-H(120)	114.3(14)
C(112)-N(12P)-H(120)	121.4(14)
C(16P)-N(11P)-C(12P)	115.81(15)
N(11P)-C(12P)-N(13P)	125.34(15)
N(11P)-C(12P)-N(12P)	119.14(15)
N(13P)-C(12P)-N(12P)	115.53(15)
C(14P)-N(13P)-C(12P)	115.98(15)
N(13P)-C(14P)-C(15P)	123.49(17)

Table S3.1.6.3. *Cont.*

N(13P)-C(14P)-H(14P)	118.3
C(15P)-C(14P)-H(14P)	118.3
C(16P)-C(15P)-C(14P)	115.77(16)
C(16P)-C(15P)-H(15P)	122.1
C(14P)-C(15P)-H(15P)	122.1
N(11P)-C(16P)-C(15P)	123.59(16)
N(11P)-C(16P)-H(16P)	118.2
C(15P)-C(16P)-H(16P)	118.2
C(22)-C(21)-C(26)	116.20(16)
C(22)-C(21)-C(212)	122.58(15)
C(26)-C(21)-C(212)	121.21(16)
C(23)-C(22)-C(21)	123.08(17)
C(23)-C(22)-N(21)	116.21(17)
C(21)-C(22)-N(21)	120.71(16)
O(22)-N(21)-O(21)	123.88(17)
O(22)-N(21)-C(22)	117.63(16)
O(21)-N(21)-C(22)	118.46(17)
C(24)-C(23)-C(22)	119.02(18)
C(24)-C(23)-H(23)	120.5
C(22)-C(23)-H(23)	120.5
C(25)-C(24)-C(23)	119.71(18)
C(25)-C(24)-H(24)	120.1
C(23)-C(24)-H(24)	120.1
C(24)-C(25)-C(26)	120.58(17)
C(24)-C(25)-H(25)	119.7
C(26)-C(25)-H(25)	119.7
C(25)-C(26)-C(21)	121.32(17)
C(25)-C(26)-H(26)	119.3
C(21)-C(26)-H(26)	119.3
O(212)-C(212)-N(22P)	109.60(14)
O(212)-C(212)-C(21)	112.10(13)
N(22P)-C(212)-C(21)	108.38(14)
O(212)-C(212)-H(212)	108.9
N(22P)-C(212)-H(212)	108.9
C(21)-C(212)-H(212)	108.9
C(212)-O(212)-H(221)	108.2(14)
C(22P)-N(22P)-C(212)	123.83(14)
C(22P)-N(22P)-H(220)	120.4(15)
C(212)-N(22P)-H(220)	115.4(15)
C(22P)-N(21P)-C(26P)	115.55(14)

Table S3.1.6.3. *Cont.*

N(21P)-C(22P)-N(23P)	125.70(14)
N(21P)-C(22P)-N(22P)	118.35(14)
N(23P)-C(22P)-N(22P)	115.94(15)
C(24P)-N(23P)-C(22P)	115.82(14)
N(23P)-C(24P)-C(25P)	123.24(16)
N(23P)-C(24P)-H(24P)	118.4
C(25P)-C(24P)-H(24P)	118.4
C(26P)-C(25P)-C(24P)	116.28(16)
C(26P)-C(25P)-H(25P)	121.9
C(24P)-C(25P)-H(25P)	121.9
N(21P)-C(26P)-C(25P)	123.39(16)
N(21P)-C(26P)-H(26P)	118.3
C(25P)-C(26P)-H(26P)	118.3

Symmetry transformations used to generate equivalent atoms.

Table 3.1.6.4. Anisotropic displacement parameters ($Å^2$x 10^3)for 1. The anisotropic displacement factor exponent takes the form: $-2\pi^2[h^2a^{*2}U^{11} + ... + 2 h k a^* b^* U^{12}]$.

	U^{11}	U^{22}	U^{33}	U^{23}	U^{13}	U^{12}
C(11)	31(1)	18(1)	31(1)	3(1)	14(1)	6(1)
C(12)	32(1)	29(1)	27(1)	4(1)	13(1)	5(1)
N(11)	30(1)	42(1)	31(1)	1(1)	9(1)	0(1)
O(11)	44(1)	66(1)	33(1)	10(1)	10(1)	−10(1)
O(12)	43(1)	44(1)	41(1)	−10(1)	10(1)	−5(1)
C(13)	30(1)	31(1)	40(1)	3(1)	13(1)	4(1)
C(14)	38(1)	30(1)	40(1)	5(1)	24(1)	7(1)
C(15)	45(1)	24(1)	31(1)	2(1)	19(1)	8(1)
C(16)	39(1)	20(1)	34(1)	0(1)	15(1)	4(1)
C(112)	30(1)	19(1)	32(1)	0(1)	12(1)	2(1)
O(112)	30(1)	20(1)	45(1)	−2(1)	10(1)	−1(1)
N(12P)	35(1)	14(1)	33(1)	3(1)	16(1)	1(1)
N(11P)	29(1)	19(1)	24(1)	1(1)	8(1)	0(1)
C(12P)	23(1)	19(1)	26(1)	1(1)	6(1)	1(1)
N(13P)	30(1)	21(1)	36(1)	2(1)	17(1)	3(1)
C(14P)	37(1)	23(1)	40(1)	3(1)	19(1)	8(1)
C(15P)	33(1)	29(1)	32(1)	6(1)	17(1)	7(1)
C(16P)	29(1)	22(1)	26(1)	5(1)	7(1)	0(1)
C(21)	26(1)	15(1)	27(1)	1(1)	10(1)	1(1)
C(22)	29(1)	24(1)	26(1)	1(1)	7(1)	2(1)
N(21)	35(1)	40(1)	29(1)	−3(1)	6(1)	−7(1)
O(21)	48(1)	50(1)	28(1)	5(1)	9(1)	−6(1)
O(22)	78(1)	51(1)	47(1)	−22(1)	22(1)	−27(1)
C(23)	25(1)	29(1)	41(1)	5(1)	4(1)	−3(1)
C(24)	28(1)	32(1)	46(1)	6(1)	18(1)	3(1)

Table S3.1.6.4. *Cont.*

	U^{11}	U^{22}	U^{33}	U^{23}	U^{13}	U^{12}
C(25)	38(1)	25(1)	37(1)	2(1)	21(1)	6(1)
C(26)	33(1)	19(1)	29(1)	−2(1)	12(1)	0(1)
C(212)	27(1)	17(1)	24(1)	−2(1)	10(1)	0(1)
O(212)	30(1)	22(1)	33(1)	−5(1)	8(1)	−3(1)
N(22P)	33(1)	14(1)	30(1)	2(1)	16(1)	2(1)
N(21P)	24(1)	17(1)	27(1)	−1(1)	10(1)	1(1)
C(22P)	22(1)	18(1)	24(1)	−1(1)	7(1)	0(1)
N(23P)	27(1)	17(1)	29(1)	0(1)	12(1)	2(1)
C(24P)	27(1)	23(1)	31(1)	−2(1)	12(1)	4(1)
C(25P)	26(1)	25(1)	26(1)	−1(1)	11(1)	2(1)
C(26P)	26(1)	21(1)	25(1)	1(1)	8(1)	−2(1)

Table S3.1.6.5. Hydrogen coordinates (× 10^4) and isotropic displacement parameters ($Å^2$ × 10^3) for 1.

	x	y	z	U(eq)
H(13)	5572	7345	2093	39
H(14)	5378	6911	458	40
H(15)	4126	5909	−382	38
H(16)	3092	5261	386	36
H(112)	3210	4844	2738	31
H(121)	2505(14)	3840(30)	1588(15)	47
H(120)	2710(13)	7520(20)	2289(14)	31
H(14P)	1420	8947	4356	38
H(15P)	1127	6944	5138	36
H(16P)	1604	4826	4681	31
H(23)	−593	−130	2457	39
H(24)	−768	502	3957	41
H(25)	304	1683	5004	38
H(26)	1530	2308	4553	31
H(212)	1834	2638	2339	26
H(221)	2448(14)	3630(30)	3592(15)	42
H(220)	2290(13)	70(20)	2875(14)	29
H(24P)	3693	−1398	845	31
H(25P)	3937	610	46	30
H(26P)	3401	2698	462	28

Table S3.1.6.6. Torsion angles [°] for 1.

C(16)-C(11)-C(12)-C(13)	−0.8(3)
C(112)-C(11)-C(12)-C(13)	−178.35(18)
C(16)-C(11)-C(12)-N(11)	175.49(17)
C(112)-C(11)-C(12)-N(11)	−2.1(3)
C(11)-C(12)-N(11)-O(12)	−139.88(18)
C(13)-C(12)-N(11)-O(12)	36.7(2)
C(11)-C(12)-N(11)-O(11)	42.2(3)

Table S3. *Cont.*

C(13)-C(12)-N(11)-O(11)	−141.19(18)
C(11)-C(12)-C(13)-C(14)	−0.2(3)
N(11)-C(12)-C(13)-C(14)	−176.71(17)
C(12)-C(13)-C(14)-C(15)	1.3(3)
C(13)-C(14)-C(15)-C(16)	−1.2(3)
C(14)-C(15)-C(16)-C(11)	0.1(3)
C(12)-C(11)-C(16)-C(15)	0.9(3)
C(112)-C(11)-C(16)-C(15)	178.50(17)
C(12)-C(11)-C(112)-O(112)	−175.98(16)
C(16)-C(11)-C(112)-O(112)	6.6(2)
C(12)-C(11)-C(112)-N(12P)	63.3(2)
C(16)-C(11)-C(112)-N(12P)	−114.12(18)
O(112)-C(112)-N(12P)-C(12P)	83.1(2)
C(11)-C(112)-N(12P)-C(12P)	−155.41(16)
C(16P)-N(11P)-C(12P)-N(13P)	1.2(3)
C(16P)-N(11P)-C(12P)-N(12P)	−179.26(15)
C(112)-N(12P)-C(12P)-N(11P)	−2.4(3)
C(112)-N(12P)-C(12P)-N(13P)	177.17(16)
N(11P)-C(12P)-N(13P)-C(14P)	−1.9(3)
N(12P)-C(12P)-N(13P)-C(14P)	178.51(16)
C(12P)-N(13P)-C(14P)-C(15P)	1.1(3)
N(13P)-C(14P)-C(15P)-C(16P)	0.4(3)
C(12P)-N(11P)-C(16P)-C(15P)	0.5(3)
C(14P)-C(15P)-C(16P)-N(11P)	−1.2(3)
C(26)-C(21)-C(22)-C(23)	0.0(3)
C(212)-C(21)-C(22)-C(23)	178.39(17)
C(26)-C(21)-C(22)-N(21)	−179.56(16)
C(212)-C(21)-C(22)-N(21)	−1.2(3)
C(23)-C(22)-N(21)-O(22)	−40.7(3)
C(21)-C(22)-N(21)-O(22)	138.87(19)
C(23)-C(22)-N(21)-O(21)	137.44(19)
C(21)-C(22)-N(21)-O(21)	−43.0(3)
C(21)-C(22)-C(23)-C(24)	2.7(3)
N(21)-C(22)-C(23)-C(24)	−177.71(17)
C(22)-C(23)-C(24)-C(25)	−3.2(3)
C(23)-C(24)-C(25)-C(26)	1.1(3)
C(24)-C(25)-C(26)-C(21)	1.7(3)
C(22)-C(21)-C(26)-C(25)	−2.2(3)
C(212)-C(21)-C(26)-C(25)	179.36(16)
C(22)-C(21)-C(212)-O(212)	−178.49(16)
C(26)-C(21)-C(212)-O(212)	−0.2(2)
C(22)-C(21)-C(212)-N(22P)	−57.4(2)
C(26)-C(21)-C(212)-N(22P)	120.93(17)
O(212)-C(212)-N(22P)-C(22P)	−91.53(18)
C(21)-C(212)-N(22P)-C(22P)	145.84(16)
C(26P)-N(21P)-C(22P)-N(23P)	−1.0(2)

Table S3. *Cont.*

C(26P)-N(21P)-C(22P)-N(22P)	−179.68(15)
C(212)-N(22P)-C(22P)-N(21P)	3.4(2)
C(212)-N(22P)-C(22P)-N(23P)	−175.44(15)
N(21P)-C(22P)-N(23P)-C(24P)	1.2(2)
N(22P)-C(22P)-N(23P)-C(24P)	179.90(15)
C(22P)-N(23P)-C(24P)-C(25P)	−0.2(2)
N(23P)-C(24P)-C(25P)-C(26P)	−0.9(3)
C(22P)-N(21P)-C(26P)-C(25P)	−0.2(2)
C(24P)-C(25P)-C(26P)-N(21P)	1.1(3)

Symmetry transformations used to generate equivalent atoms.

Table S3.1.6.7. Hydrogen bonds for 1 [Å and °].

D-H...A	d(D-H)	d(H...A)	d(D...A)	<(DHA)
O(112)-H(121)...N(21P)	0.93(2)	1.93(3)	2.848(2)	171(2)
O(212)-H(221)...N(11P)	0.92(2)	1.93(2)	2.838(2)	171(2)
N(12P)-H(120)...N(23P)#1	0.86(2)	2.20(2)	3.052(2)	171.3(19)
N(22P)-H(220)...N(13P)#2	0.80(2)	2.23(2)	3.032(2)	176(2)
C(23)-H(23)...N(11P)#3	0.95	2.71	3.424(3)	132.8
C(15)-H(15)...O(12)#4	0.95	2.58	3.249(3)	128.0
C(15P)-H(15P)...O(22)#5	0.95	2.67	3.523(3)	150.1
C(212)-H(212)...O(112)	1.00	2.56	3.417(2)	143.6
C(24)-H(24)...O(112)#3	0.95	2.57	3.412(3)	148.1
C(25)-H(25)...O(21)#5	0.95	2.53	3.260(3)	133.8
C(25P)-H(25P)...O(11)#6	0.95	2.67	3.487(2)	144.5

Symmetry transformations used to generate equivalent atoms: #1 x,y+1,z #2 x,y-1,z #3 -x,y-1/2,-z+1/2 #4 x,-y+3/2,z-1/2 #5 x,-y+1/2,z+1/2 #6 x,-y+1/2,z-1/2 .

3.2. (2,4-Dinitrophenyl)(pyrimidin-2-ylamino)methanol (2)

3.2.1. Synthesis

Acetonitrilic solution (3 mL) of 2,4-dinitrobenzaldehyde (40 mg) was added to an acetonitrilic solution (3 mL) of 2-aminopyrimidine (19 mg). The reaction mixture after complete dissolution was stirred for 2 hours at 50 ˚C. The title compound crystallised directly from the mother liquor. Upon standing 4 days at the room temperature, the solution deposited yellow crystal blocks. The crystals were filtered off, washed with a small amount of acetonitrile and diethyl ether then dried in the air to afford (2,4-dinitrophenyl)(pyrimidin-2-ylamino)methanol—(51 mg, 88%), mp 108 °C.

3.2.2. Elemental Analysis

	% C	% H	% N
Calculated	45.37	3.11	24.05
Found	45.39	2.86	24.14

3.2.3. Mass Spectrometry

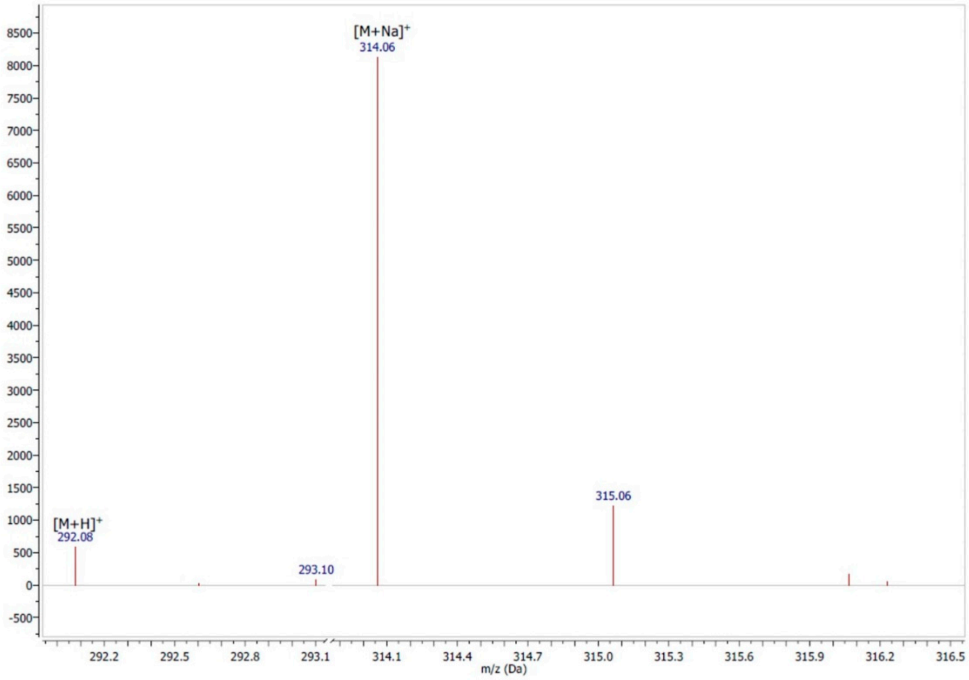

3.2.4. NMR Spectroscopy

^{1}H-NMR

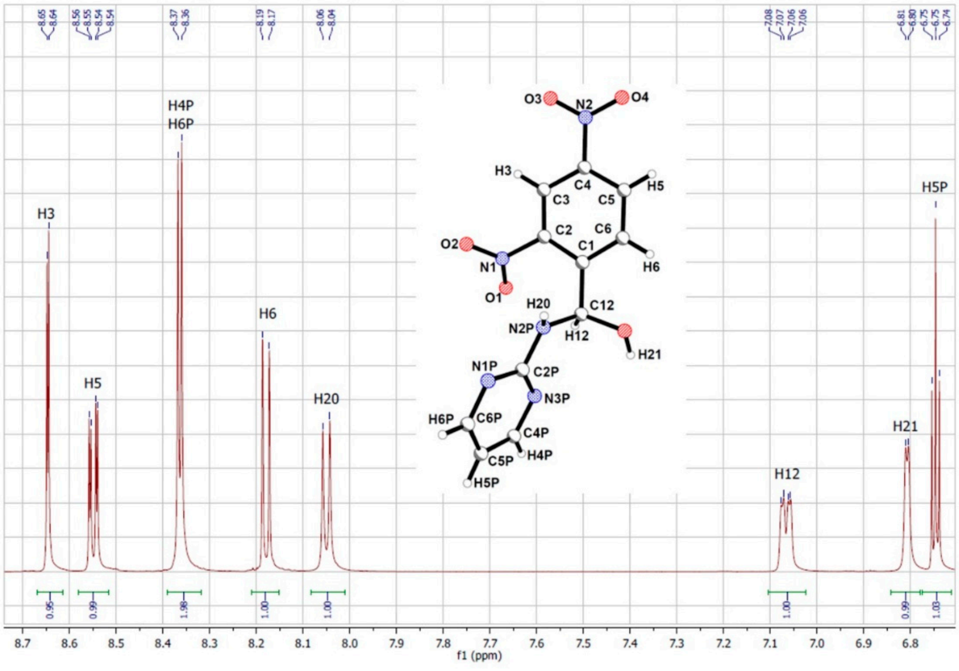

^{1}H-NMR (600 MHz, DMSO, RT) δ: 8.65 (d, $^{4}J_{H3,H5}$ = 2.4 Hz, 1H, H3), 8.55 (dd, $^{3}J_{H5,H6}$ = 9.0 Hz, $^{4}J_{H5,H3}$ = 2.4 Hz, 1H, H5), 8.36 (d, $^{3}J_{H6P/H6P,H5P}$ = 4.8, 2H, H4P,H6P), 8.18 (d, $^{3}J_{H6,H5}$ = 9.0 Hz, 1H, H6), 8.05 (d, $^{3}J_{H20,H12}$ = 9.0 Hz, 1H, H20), 7.07 (dd, $^{3}J_{H12,H20}$ = 9.0 Hz, $^{3}J_{H12,H21}$ = 3.6 Hz, 1H, H12), 6.81 (d, $^{3}J_{H21,H12}$ = 3.6 Hz, 1H, H21), 6.75 (t, $^{3}J_{H5P,H4P/H46}$ = 4.8 Hz, 1H, H5P).

^{13}C-NMR

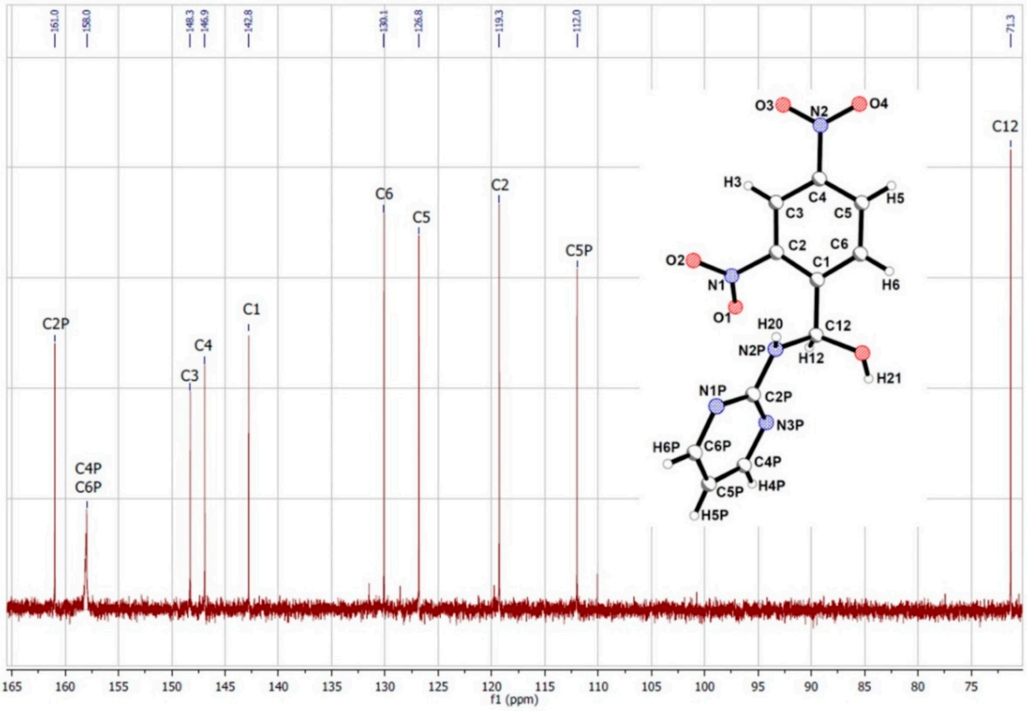

^{13}C-NMR (150.9 MHz, DMSO, RT) δ: 161.0 (C2P), 158.0 (C4P, C6P), 148.3 (C3), 146.9 (C4), 142.8 (C1), 130.1 (C6), 126.8 (C5), 119.3 (C2), 112.0 (C5P), 71.3 (C12).

COSY

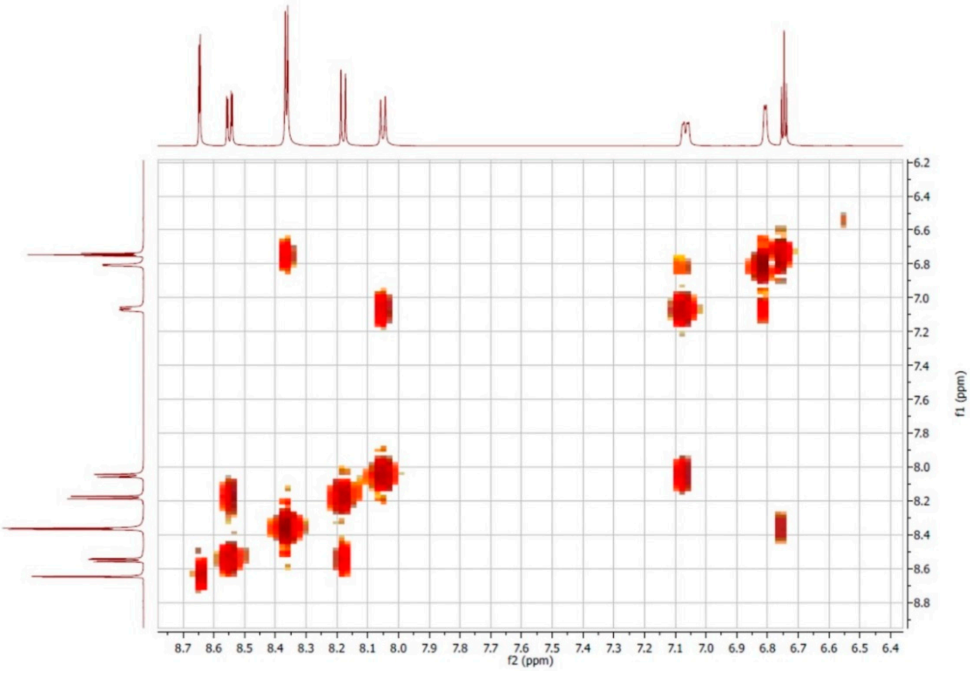

HMQC

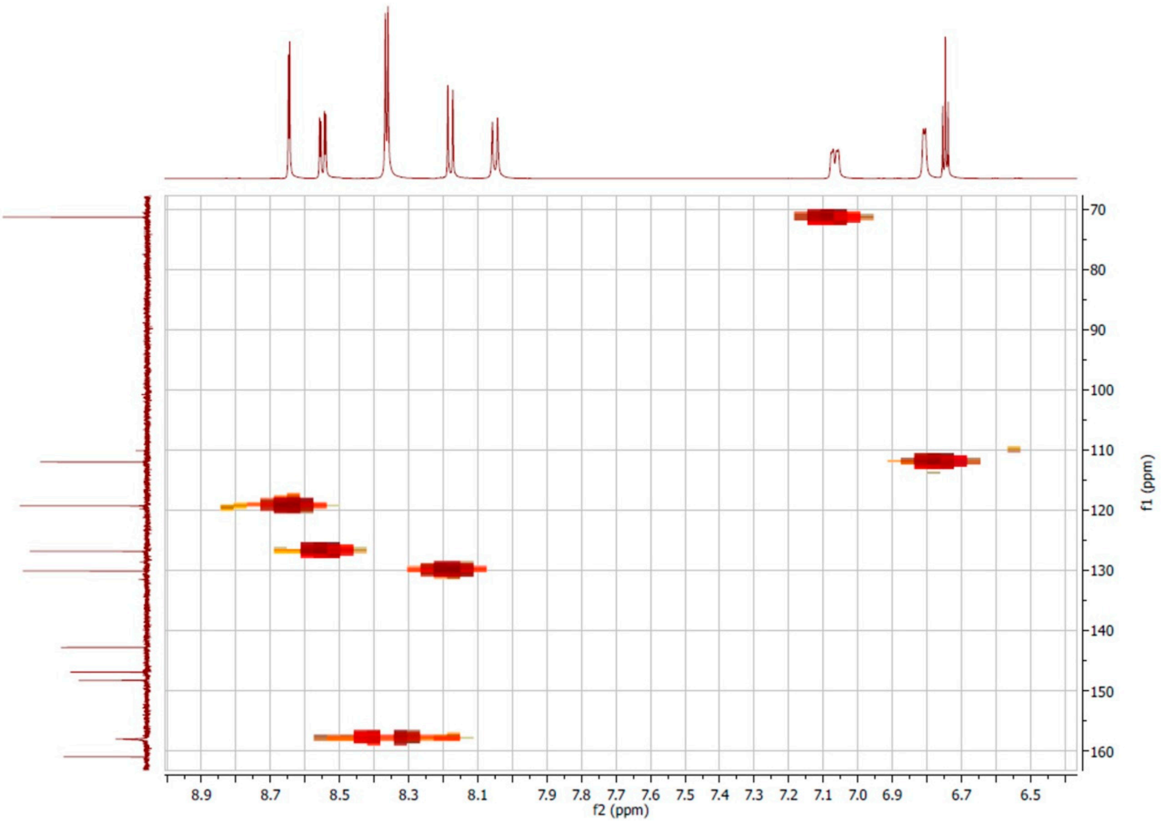

HMBC

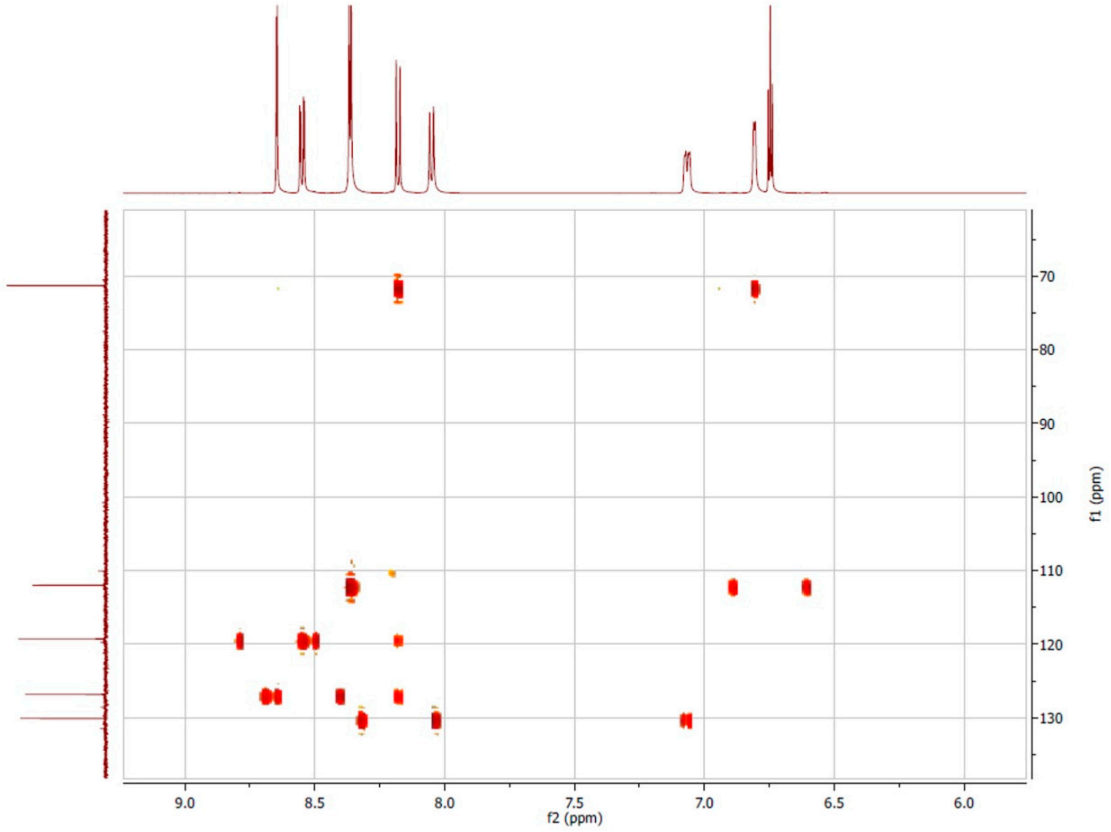

3.2.5. IR Spectroscopy

IR (KBr, cm^{-1}): 3343 s, 3100 m, 3026 m, 1611 m, 1589 s, 1574 vs, 1531 vs, 1511 vs, 1457 vs, 1421 m, 1343 s, 1298 m, 1246 s, 1197 w, 1116 m, 1090 w, 1073 m, 1049 s, 1032 s, 998 w, 953 vw, 926 vw, 848 m, 821 w, 807 m, 759 w, 736 w, 669 w, 645 w, 628 w, 585 m, 515 m.

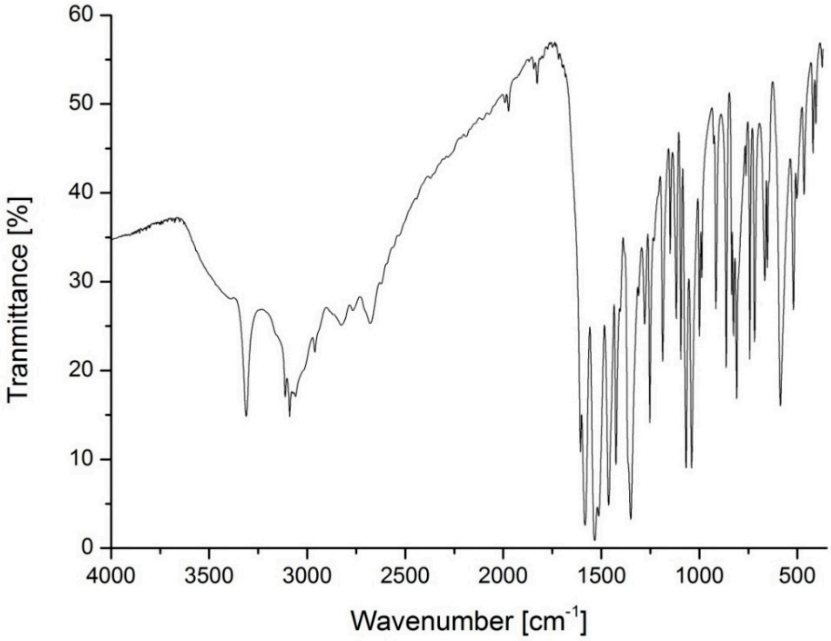

3.2.6. Crystallography

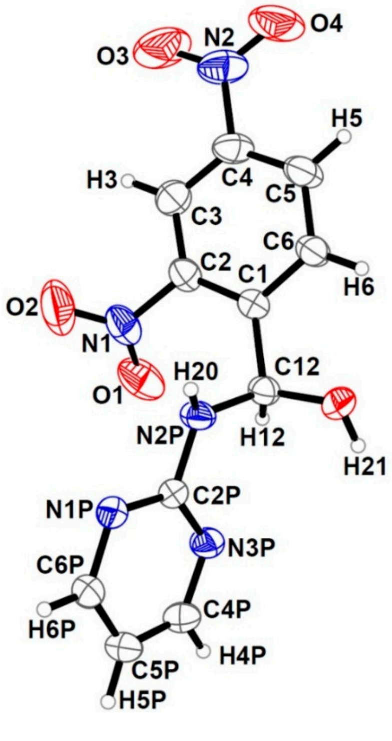

Figure S2. Molecular structure and labelling for (2,4-dinitrophenyl)(pyrimidin-2-ylamino)methanol (2). Displacement ellipsoids are shown at the 50% probability level.

Table S3.2.6.1. Crystal data and structure refinement for 2.

Identification Code	2	
Empirical formula	C11 H9 N5 O5	
Formula weight	291.23	
Temperature	296(2) K	
Wavelength	0.71073 Å	
Crystal system	Triclinic	
Space group	P -1	
Unit cell dimensions	a = 5.509(3) Å	α = 69.55(3)°
	b = 10.866(4) Å	β= 89.43(3)°
	c = 11.475(4) Å	γ = 80.60(3)°
Volume	634.1(5) $Å^3$	
Z	2	
Density (calculated)	1.525 Mg/m^3	
Absorption coefficient	0.124 mm^{-1}	
F(000)	300	
Crystal size	0.65 × 0.38 × 0.16 mm^3	
Theta range for data collection	3.23 to 34.56°	
Index ranges	−8 ≤ h ≤ 8, −17≤k≤17, −18 ≤ l ≤ 18	
Reflections collected	44906	
Independent reflections	5244 [R(int) = 0.0233]	
Completeness to theta = 27.00°	99.9%	
Absorption correction	Semi-empirical from equivalents	
Max. and min. transmission	1.00000 and 0.96594	
Refinement method	Full-matrix least-squares on F^2	
Data / restraints / parameters	5244/0/196	
Goodness-of-fit on F^2	1.040	
Final R indices [I>2sigma(I)]	R1 = 0.0476, wR2 = 0.1315	
R indices (all data)	R1 = 0.0596, wR2 = 0.1417	
Largest diff. peak and hole	0.306 and −0.297 e.$Å^{-3}$	

Table S3.2.6.2. Atomic coordinates (× 10^4) and equivalent isotropic displacement parameters ($Å^2$ × 10^3) for 2. U(eq) is defined as one third of the trace of the orthogonalized U^{ij} tensor.

	x	y	z	U(eq)
C(1)	10,743(2)	4726(1)	7437(1)	30(1)
C(2)	9029(2)	4648(1)	8352(1)	35(1)
C(3)	8936(2)	3500(1)	9365(1)	42(1)
C(4)	10,701(2)	2403(1)	9469(1)	42(1)
C(5)	12,462(2)	2417(1)	8613(1)	45(1)
C(6)	12,458(2)	3583(1)	7597(1)	39(1)
N(1)	7236(2)	5826(1)	8315(1)	47(1)
O(1)	8021(2)	6865(1)	8117(1)	64(1)
O(2)	5105(2)	5684(1)	8523(1)	78(1)
N(2)	10,692(3)	1173(1)	10,553(1)	57(1)
O(3)	9079(3)	1156(1)	11,266(1)	98(1)

Table S3.2.6.2. *Cont.*

	x	y	z	U(eq)
O(4)	12,308(3)	232(1)	10,675(1)	77(1)
C(12)	10,695(2)	5969(1)	6278(1)	31(1)
O(12)	12,465(1)	5693(1)	5469(1)	39(1)
N(2P)	8216(2)	6328(1)	5731(1)	33(1)
N(1P)	5048(1)	7763(1)	4423(1)	33(1)
C(2P)	7294(2)	7599(1)	4961(1)	28(1)
N(3P)	8628(2)	8559(1)	4819(1)	35(1)
C(4P)	7643(2)	9769(1)	4037(1)	40(1)
C(5P)	5416(2)	10,037(1)	3394(1)	41(1)
C(6P)	4151(2)	8987(1)	3632(1)	37(1)

Table S3.2.6.3. Bond lengths [Å] and angles [°] for 2.

C(1)-C(6)	1.3894(14)
C(1)-C(2)	1.3946(14)
C(1)-C(12)	1.5253(14)
C(2)-C(3)	1.3847(16)
C(2)-N(1)	1.4722(14)
C(3)-C(4)	1.3800(17)
C(3)-H(3)	0.9300
C(4)-C(5)	1.3711(17)
C(4)-N(2)	1.4741(16)
C(5)-C(6)	1.3898(16)
C(5)-H(5)	0.9300
C(6)-H(6)	0.9300
N(1)-O(2)	1.2197(17)
N(1)-O(1)	1.2212(16)
N(2)-O(3)	1.2000(19)
N(2)-O(4)	1.2098(19)
C(12)-O(12)	1.4090(13)
C(12)-N(2P)	1.4436(14)
C(12)-H(12)	0.9800
O(12)-H(21)	0.845(18)
N(2P)-C(2P)	1.3685(13)
N(2P)-H(20)	0.887(14)
N(1P)-C(6P)	1.3368(14)
N(1P)-C(2P)	1.3455(13)
C(2P)-N(3P)	1.3358(12)
N(3P)-C(4P)	1.3346(14)
C(4P)-C(5P)	1.3731(18)
C(4P)-H(4P)	0.9300

Table S3.2.6.3. *Cont.*

C(5P)-C(6P)	1.3782(16)
C(5P)-H(5P)	0.9300
C(6P)-H(6P)	0.9300
C(6)-C(1)-C(2)	116.64(9)
C(6)-C(1)-C(12)	120.69(9)
C(2)-C(1)-C(12)	122.61(8)
C(3)-C(2)-C(1)	123.47(9)
C(3)-C(2)-N(1)	115.53(10)
C(1)-C(2)-N(1)	120.96(9)
C(4)-C(3)-C(2)	116.82(10)
C(4)-C(3)-H(3)	121.6
C(2)-C(3)-H(3)	121.6
C(5)-C(4)-C(3)	122.70(10)
C(5)-C(4)-N(2)	119.04(11)
C(3)-C(4)-N(2)	118.26(11)
C(4)-C(5)-C(6)	118.60(10)
C(4)-C(5)-H(5)	120.7
C(6)-C(5)-H(5)	120.7
C(1)-C(6)-C(5)	121.75(10)
C(1)-C(6)-H(6)	119.1
C(5)-C(6)-H(6)	119.1
O(2)-N(1)-O(1)	124.97(12)
O(2)-N(1)-C(2)	117.81(12)
O(1)-N(1)-C(2)	117.17(11)
O(3)-N(2)-O(4)	123.39(13)
O(3)-N(2)-C(4)	118.32(13)
O(4)-N(2)-C(4)	118.29(13)
O(12)-C(12)-N(2P)	112.85(8)
O(12)-C(12)-C(1)	109.25(8)
N(2P)-C(12)-C(1)	106.79(8)
O(12)-C(12)-H(12)	109.3
N(2P)-C(12)-H(12)	109.3
C(1)-C(12)-H(12)	109.3
C(12)-O(12)-H(21)	109.5(12)
C(2P)-N(2P)-C(12)	121.75(8)
C(2P)-N(2P)-H(20)	113.4(9)
C(12)-N(2P)-H(20)	116.1(9)
C(6P)-N(1P)-C(2P)	116.24(9)
N(3P)-C(2P)-N(1P)	125.79(8)
N(3P)-C(2P)-N(2P)	118.90(8)
N(1P)-C(2P)-N(2P)	115.31(8)
C(4P)-N(3P)-C(2P)	115.79(9)
N(3P)-C(4P)-C(5P)	123.33(10)
N(3P)-C(4P)-H(4P)	118.3

Table S3.2.6.3. *Cont.*

C(5P)-C(4P)-H(4P)	118.3
C(4P)-C(5P)-C(6P)	116.34(9)
C(4P)-C(5P)-H(5P)	121.8
C(6P)-C(5P)-H(5P)	121.8
N(1P)-C(6P)-C(5P)	122.41(10)
N(1P)-C(6P)-H(6P)	118.8
C(5P)-C(6P)-H(6P)	118.8

Symmetry transformations used to generate equivalent atoms.

Table S3.2.6.4. Anisotropic displacement parameters ($Å^2 \times 10^3$)for 2. The anisotropic displacement factor exponent takes the form: $-2\pi^2[h^2a^{*2}U^{11} + ... + 2 h k a^* b^* U^{12}]$.

	U^{11}	U^{22}	U^{33}	U^{23}	U^{13}	U^{12}
C(1)	31(1)	27(1)	31(1)	−9(1)	−1(1)	−2(1)
C(2)	35(1)	35(1)	34(1)	−13(1)	0(1)	1(1)
C(3)	46(1)	45(1)	31(1)	−11(1)	7(1)	−6(1)
C(4)	58(1)	33(1)	29(1)	−5(1)	1(1)	−6(1)
C(5)	57(1)	30(1)	37(1)	−6(1)	2(1)	7(1)
C(6)	43(1)	31(1)	36(1)	−7(1)	6(1)	4(1)
N(1)	45(1)	50(1)	41(1)	−20(1)	−2(1)	11(1)
O(1)	77(1)	44(1)	70(1)	−29(1)	−5(1)	12(1)
O(2)	44(1)	93(1)	93(1)	−37(1)	12(1)	11(1)
N(2)	85(1)	44(1)	35(1)	−3(1)	3(1)	−14(1)
O(3)	139(1)	71(1)	65(1)	1(1)	50(1)	−22(1)
O(4)	100(1)	46(1)	58(1)	10(1)	−9(1)	5(1)
C(12)	29(1)	25(1)	36(1)	−8(1)	−1(1)	−5(1)
O(12)	37(1)	31(1)	47(1)	−9(1)	12(1)	−11(1)
N(2P)	32(1)	24(1)	38(1)	−4(1)	−5(1)	−7(1)
N(1P)	26(1)	32(1)	33(1)	−5(1)	2(1)	−5(1)
C(2P)	27(1)	26(1)	28(1)	−7(1)	4(1)	−5(1)
N(3P)	34(1)	27(1)	41(1)	−7(1)	1(1)	−8(1)
C(4P)	44(1)	27(1)	44(1)	−6(1)	6(1)	−9(1)
C(5P)	44(1)	30(1)	38(1)	0(1)	4(1)	0(1)
C(6P)	30(1)	40(1)	33(1)	−4(1)	1(1)	0(1)

Table S3.2.6.5. Hydrogen coordinates (x 10^4) and isotropic displacement parameters ($Å^2 \times 10^3$) for 2.

	x	y	z	U(eq)
H(3)	7742	3469	9948	50
H(5)	13,633	1663	8711	54
H(6)	13,636	3598	7008	47
H(12)	11,089	6695	6523	37
H(21)	13,130(30)	6370(18)	5124(16)	59
H(20)	7650(30)	5707(14)	5530(12)	39
H(4P)	8508	10,466	3921	48
H(5P)	4795	10,880	2828	50
H(6P)	2618	9135	3229	45

Table S3.2.6.6. Torsion angles [°] for 2.

C(6)-C(1)-C(2)-C(3)	−1.63(15)
C(12)-C(1)-C(2)-C(3)	175.40(9)
C(6)-C(1)-C(2)-N(1)	175.95(10)
C(12)-C(1)-C(2)-N(1)	−7.03(14)
C(1)-C(2)-C(3)-C(4)	1.90(17)
N(1)-C(2)-C(3)-C(4)	−175.79(10)
C(2)-C(3)-C(4)-C(5)	−0.86(18)
C(2)-C(3)-C(4)-N(2)	178.63(10)
C(3)-C(4)-C(5)-C(6)	−0.36(19)
N(2)-C(4)-C(5)-C(6)	−179.85(11)
C(2)-C(1)-C(6)-C(5)	0.29(16)
C(12)-C(1)-C(6)-C(5)	−176.79(10)
C(4)-C(5)-C(6)-C(1)	0.65(19)
C(3)-C(2)-N(1)-O(2)	−46.36(15)
C(1)-C(2)-N(1)-O(2)	135.89(12)
C(3)-C(2)-N(1)-O(1)	131.28(12)
C(1)-C(2)-N(1)-O(1)	−46.48(14)
C(5)-C(4)-N(2)-O(3)	−176.89(15)
C(3)-C(4)-N(2)-O(3)	3.6(2)
C(5)-C(4)-N(2)-O(4)	2.95(19)
C(3)-C(4)-N(2)-O(4)	−176.56(13)
C(6)-C(1)-C(12)-O(12)	3.58(12)
C(2)-C(1)-C(12)-O(12)	−173.33(8)
C(6)-C(1)-C(12)-N(2P)	125.91(10)
C(2)-C(1)-C(12)-N(2P)	−50.99(12)
O(12)-C(12)-N(2P)-C(2P)	−82.78(11)
C(1)-C(12)-N(2P)-C(2P)	157.17(8)
C(6P)-N(1P)-C(2P)-N(3P)	3.51(14)
C(6P)-N(1P)-C(2P)-N(2P)	−177.66(8)
C(12)-N(2P)-C(2P)-N(3P)	−7.20(13)
C(12)-N(2P)-C(2P)-N(1P)	173.88(8)
N(1P)-C(2P)-N(3P)-C(4P)	−2.50(14)
N(2P)-C(2P)-N(3P)-C(4P)	178.72(9)
C(2P)-N(3P)-C(4P)-C(5P)	−0.74(16)
N(3P)-C(4P)-C(5P)-C(6P)	2.58(17)
C(2P)-N(1P)-C(6P)-C(5P)	−1.34(15)
C(4P)-C(5P)-C(6P)-N(1P)	−1.47(16)

Symmetry transformations used to generate equivalent atoms.

Table S3.2.6.7. Hydrogen bonds for 2 [Å and °].

D-H...A	d(D-H)	d(H...A)	d(D...A)	<(DHA)
O(12)-H(21)...N(1P)#1	0.845(18)	1.926(18)	2.7658(16)	172.5(17)
N(2P)-H(20)...O(12)#2	0.887(14)	2.209(14)	3.0410(15)	156.0(12)
C(6)-H(6)...N(1P)#2	0.93	2.59	3.3232(17)	135.7
C(5P)-H(5P)...O(1)#3	0.93	2.55	3.439(2)	159.3

Symmetry transformations used to generate equivalent atoms: #1 x+1,y,z #2 -x+2,-y+1,-z+1 #3 -x+1,-y+2,-z+1 .

3.3. (2-Chloro-5-nitrophenyl)(pyrimidin-2-ylamino)methanol (3a and 3b)

3.3.1. Synthesis

Acetonitrilic solution (3 mL) of 2-chloro-5-nitrobenzaldehyde (39 mg) was added to an acetonitrilic solution (3 mL) of 2-aminopyrimidine (20 mg). The reaction mixture after complete dissolution was stirred for 2 h at 50 °C. The title compound crystallised directly from the mother liquor. Upon standing 3 days at the room temperature, the solution deposited colourless crystal needles and blocks. The crystals were filtered off, washed with a small amount of acetonitrile and diethyl ether then dried in the air to afford two polymorphic forms of (2-chloro-5-nitrophenyl)(pyrimidin-2-ylamino)methanol—(54 mg, 92%), mp 93–94 °C.

3.3.2. Elemental Analysis

	% C	% H	% N
Calculated	47.07	3.23	19.96
Found	46.92	3.33	20.18

3.3.3. Mass Spectrometry

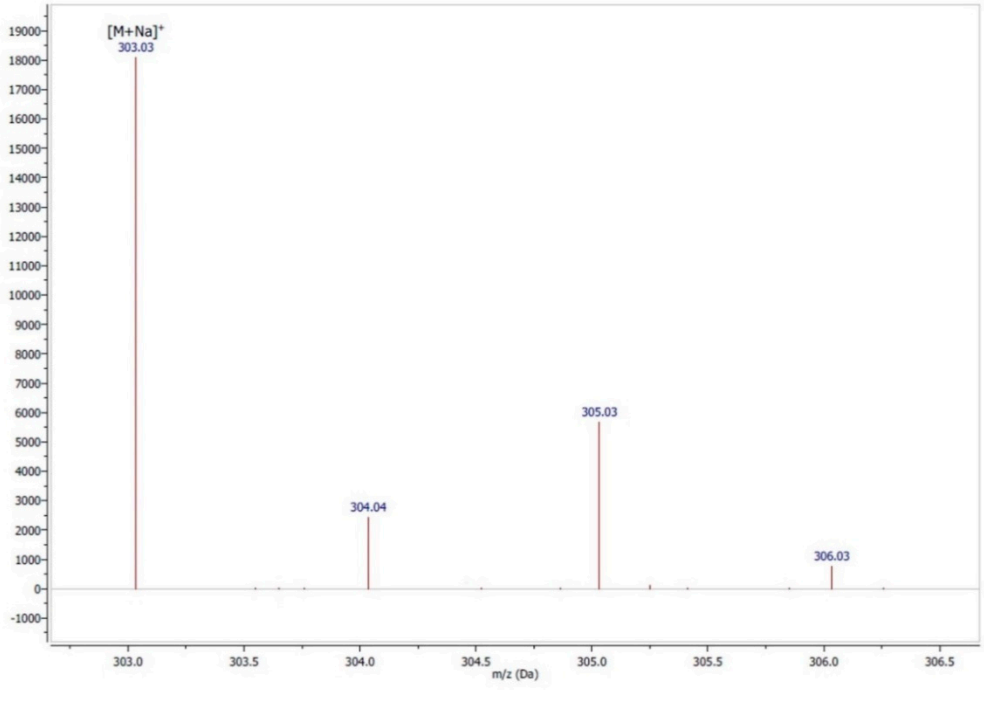

3.3.4. NMR Spectroscopy

^{1}H-NMR

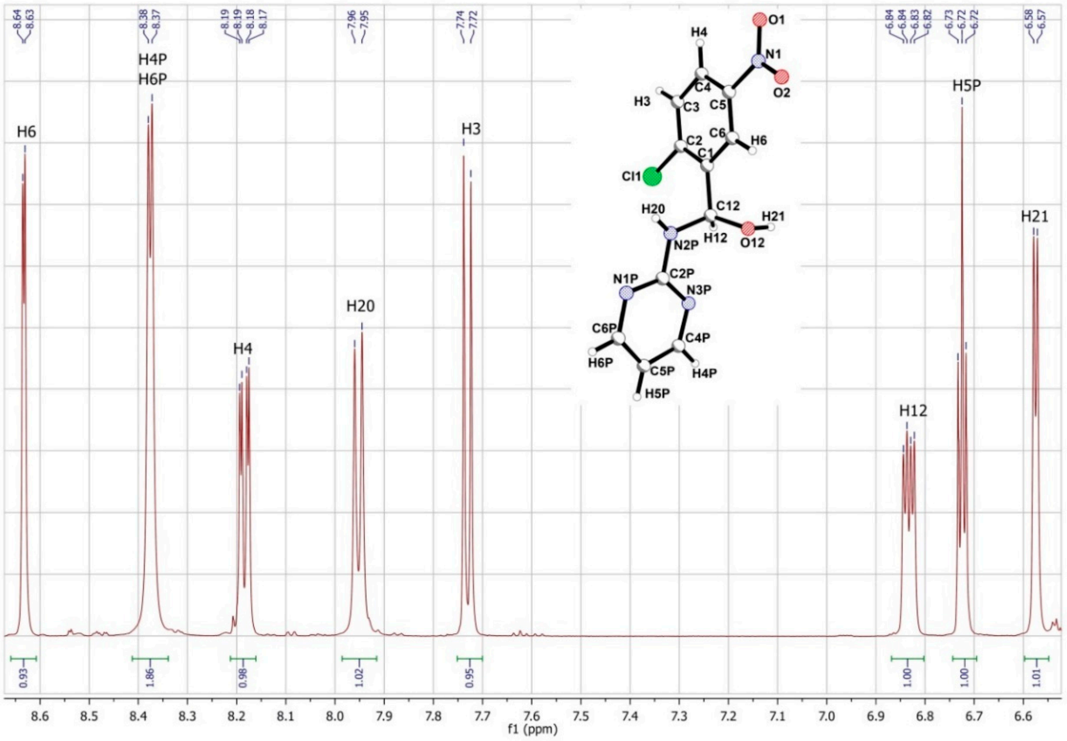

^{1}H-NMR (600 MHz, DMSO, RT) δ: 8.63 (d, $^{4}J_{H6,H4}$ = 2.4 Hz, 1H, H6), 8.38 (d, $^{3}J_{H4P/H6P,H5P}$ = 4.8 Hz, 2H, H4P,H6P), 8.18 (dd, $^{3}J_{H4,H3}$ = 8.4 Hz, $^{4}J_{H4,H6}$ = 2.4 Hz, 1H,H4), 7.95 (d, $^{3}J_{H20,H12}$ = 9.0 Hz, 1H, H20), 7.73 (d, $^{3}J_{H3,H4}$ = 8.4 Hz, 1H, H3), 6.83 (dd, $^{3}J_{H12,H20}$ = 9.0 Hz, $^{3}J_{H12,H21}$ = 4.2 Hz, 1H, H12), 6.72 (t, $^{3}J_{H5P,H4P/H6P}$ = 4.8 Hz, 1H. H5P), 6.58 (d, $^{3}J_{H21,H12}$ = 4.2 Hz, 1H, H21).

^{13}C-NMR

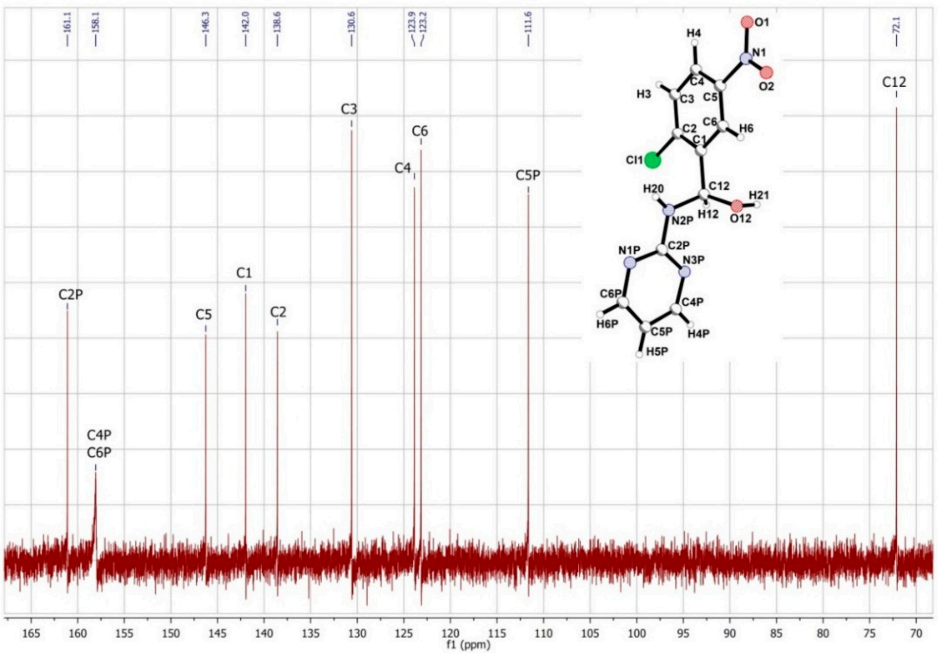

^{13}C-NMR (150.9 MHz, DMSO, RT) δ: 161.1 (C2P), 158.1 (C4P, C6P), 146.3 (C5), 142.0 (C1), 138.6 (C2), 130.6 (C3), 123.9 (C4), 123.2 (C6), 111.6 (C5P), 72.1 (C12).

COSY

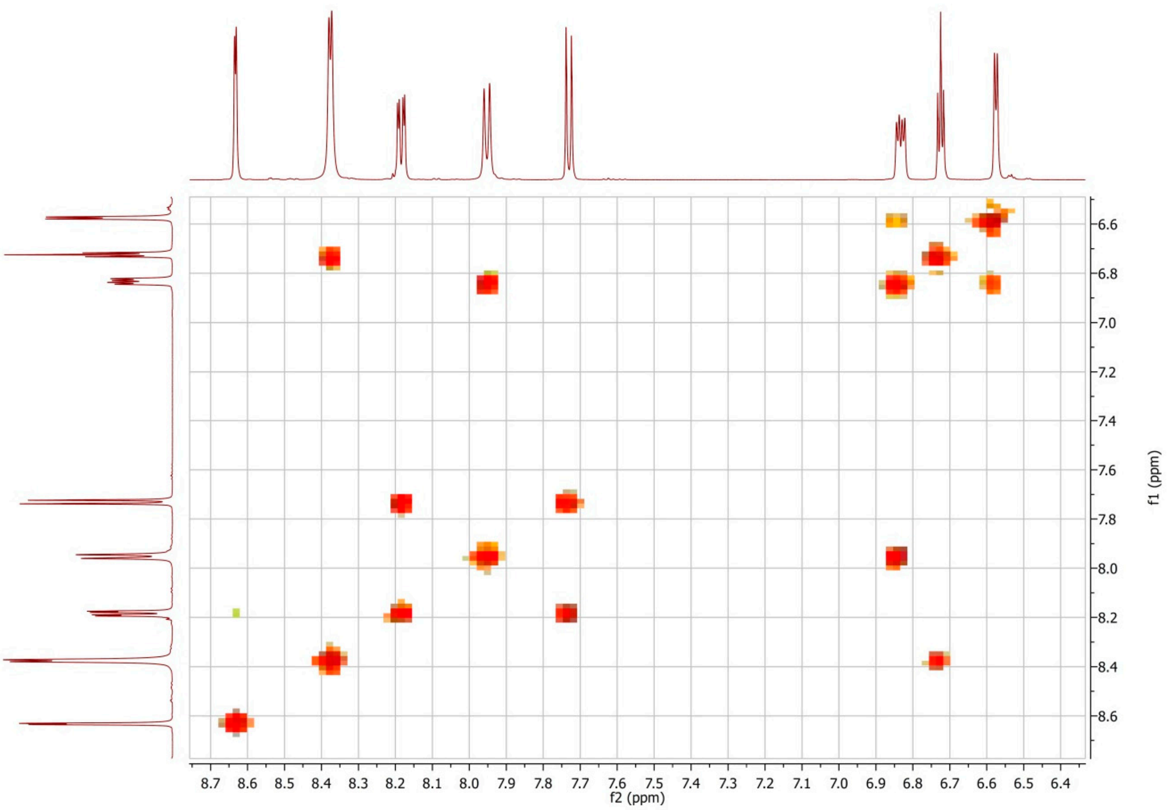

HMQC

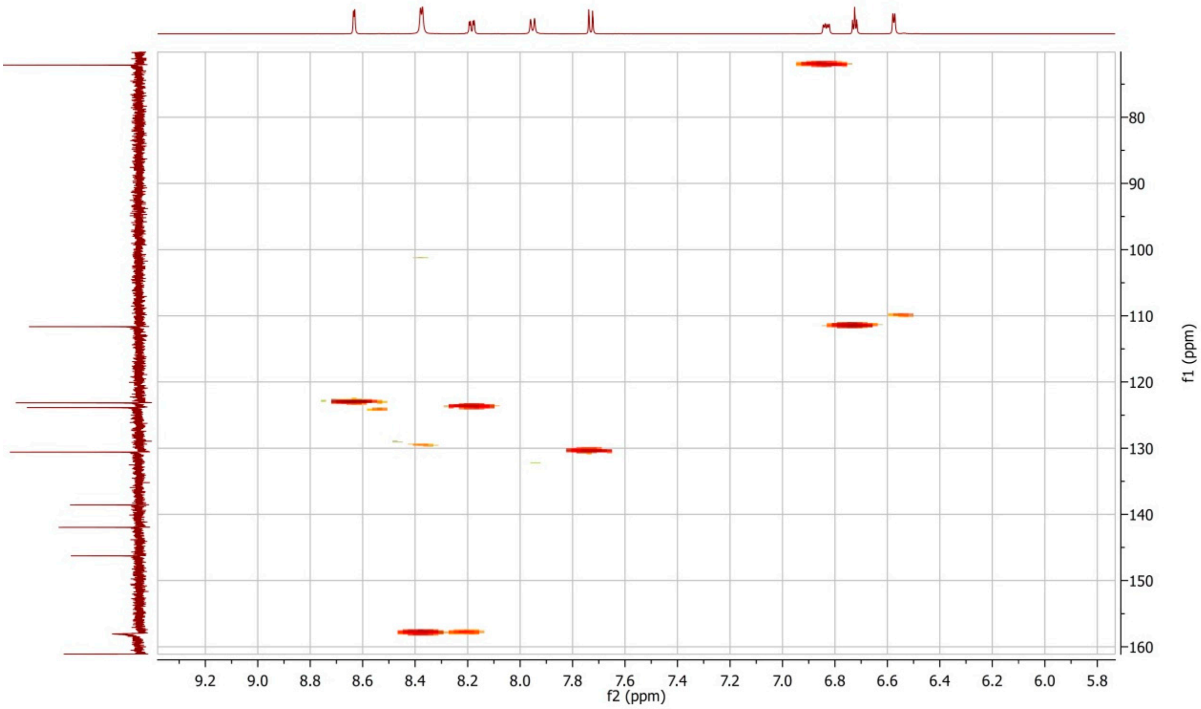

HMBC

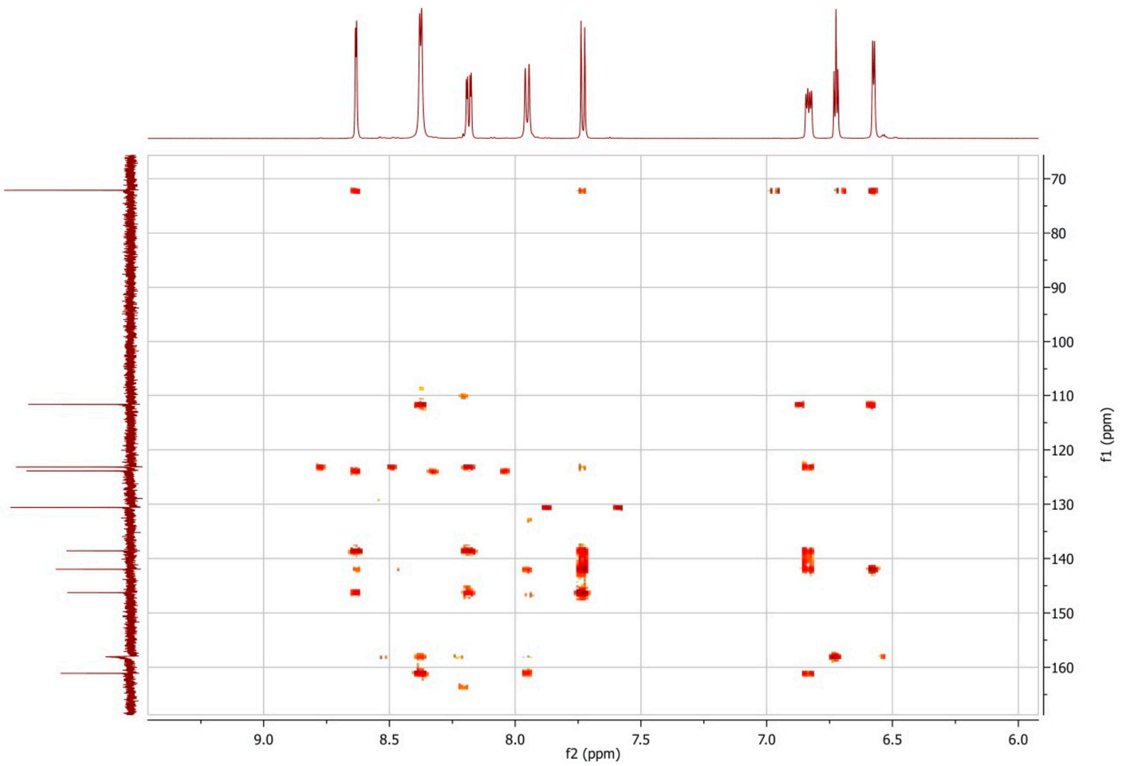

3.3.5. IR Spectroscopy

IR (KBr, cm^{-1}): 3228 s, 3109 s, 3022 m, 2361 vw, 1919 vw, 1845 vw, 1792 vw, 1700 vw, 1590 vs, 1526 vs, 1464 vs, 1422 s, 1347 vs, 1310 s, 1253 s, 1240 s, 1189 m, 1138 m, 1110 s, 1102 s, 1068 s, 1034 vs, 1000 w, 979 vw, 964 w, 948 w, 925 w, 897 w, 838 m, 832 m, 822 m, 802 s, 788 m, 742 s, 668 w, 646 m, 619 s, 565 w, 529 w, 485 m.

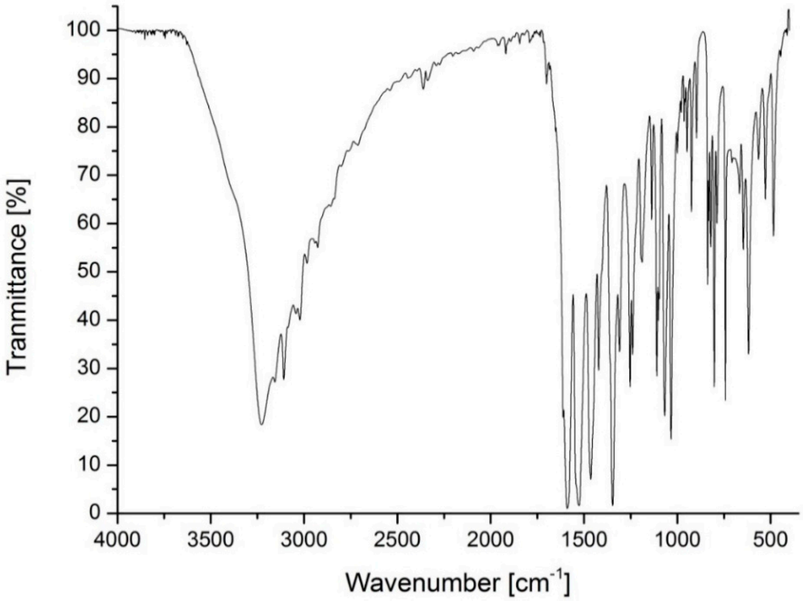

3.3.6. Crystallography

(2-chloro-5-nitrophenyl)(pyrimidin-2-ylamino)methanol (3a)

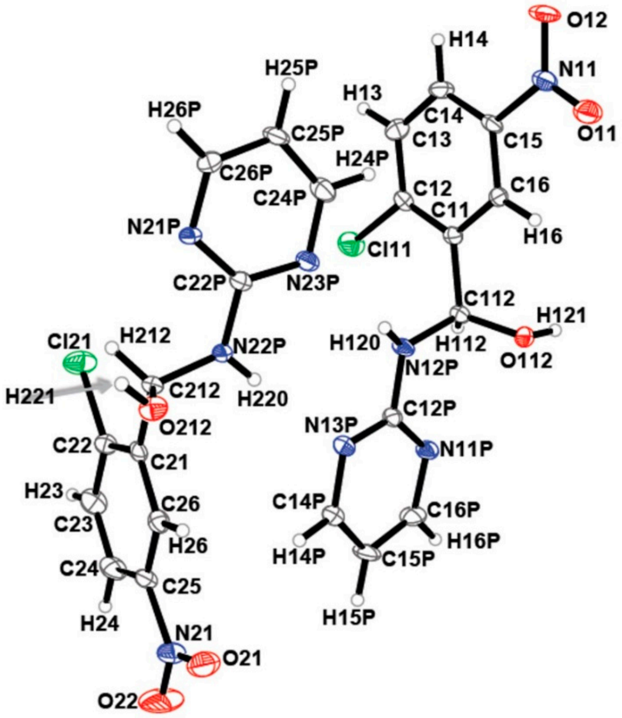

Figure S3. Molecular structure and labelling for (2-chloro-5-nitrophenyl)(pyrimidin-2-ylamino)methanol (3a). Displacement ellipsoids are shown at the 50% probability level.

Table S3.3.6.1.1. Crystal data and structure refinement for 3a.

Identification Code	3a	
Empirical formula	C11 H9 Cl N4 O3	
Formula weight	280.67	
Temperature	100(2) K	
Wavelength	0.71073 Å	
Crystal system	Triclinic	
Space group	P -1	
Unit cell dimensions	a = 9.591(4) Å	α = 73.54(3)°
	b = 10.906(4) Å	β= 88.56(3)°
	c = 13.140(5) Å	γ = 66.71(3)°
Volume	1204.8(8) Å^3	
Z	4	
Density (calculated)	1.547 Mg/m^3	
Absorption coefficient	0.327 mm^{-1}	
F(000)	576	
Crystal size	0.38 × 0.13 × 0.07 mm^3	
Theta range for data collection	2.96 to 29.99°	
Index ranges	$-12 \leq h \leq 12, -13 \leq k \leq 14, -17 \leq l \leq 18$	
Reflections collected	11880	

Table S3.3.6.1.1. *Cont.*

Identification Code	3a
Independent reflections	5848 [R(int) = 0.0448]
Completeness to theta = 27.00°	99.2%
Absorption correction	Semi-empirical from equivalents
Max. and min. transmission	1.00000 and 0.93579
Refinement method	Full-matrix least-squares on F^2
Data/restraints/parameters	5848/0/355
Goodness-of-fit on F^2	0.990
Final R indices [I > 2sigma(I)]	R1 = 0.0433, wR2 = 0.0591
R indices (all data)	R1 = 0.1021, wR2 = 0.0639
Largest diff. peak and hole	0.357 and −0.285 e.Å^{-3}

Table S3.3.6.1.2. Atomic coordinates ($\times 10^4$) and equivalent isotropic displacement parameters (Å^2 × 10^3) for 3a. U(eq) is defined as one third of the trace of the orthogonalized U^{ij} tensor.

	x	y	z	U(eq)
Cl(11)	5917(1)	4711(1)	6187(1)	30(1)
C(11)	7894(2)	1990(2)	6859(1)	14(1)
C(12)	6823(2)	3116(2)	7136(1)	17(1)
C(13)	6441(2)	2998(2)	8185(2)	24(1)
C(14)	7164(2)	1737(2)	8974(2)	22(1)
C(15)	8228(2)	641(2)	8687(1)	16(1)
C(16)	8599(2)	736(2)	7652(1)	14(1)
N(11)	9033(2)	−696(2)	9516(1)	20(1)
O(11)	9845(2)	−1720(2)	9246(1)	26(1)
O(12)	8848(2)	−724(2)	10457(1)	26(1)
C(112)	8307(2)	2111(2)	5710(1)	14(1)
O(112)	9650(2)	951(1)	5682(1)	16(1)
N(12P)	7073(2)	2146(2)	5082(1)	16(1)
N(11P)	8063(2)	2897(2)	3515(1)	18(1)
C(12P)	7062(2)	2395(2)	4005(1)	16(1)
N(13P)	6004(2)	2145(2)	3535(1)	17(1)
C(14P)	5988(2)	2425(2)	2474(2)	21(1)
C(15P)	6939(2)	2958(2)	1890(2)	27(1)
C(16P)	7966(2)	3172(2)	2458(2)	26(1)
Cl(21)	165(1)	5401(1)	3640(1)	30(1)
C(21)	1652(2)	3199(2)	2877(1)	14(1)
C(22)	961(2)	4655(2)	2649(2)	19(1)
C(23)	864(2)	5542(2)	1627(2)	24(1)
C(24)	1480(2)	4979(2)	821(2)	24(1)
C(25)	2177(2)	3547(2)	1052(2)	19(1)
C(26)	2255(2)	2657(2)	2057(1)	17(1)
N(21)	2897(2)	2926(2)	210(1)	26(1)
O(21)	3517(2)	1653(2)	424(1)	31(1)

Table S3.3.6.1.2. *Cont.*

	x	y	z	U(eq)
O(22)	2849(2)	3725(2)	−676(1)	48(1)
C(212)	1741(2)	2226(2)	3988(1)	14(1)
O(212)	1790(2)	951(2)	3892(1)	19(1)
N(22P)	3087(2)	2016(2)	4602(1)	17(1)
N(21P)	2082(2)	1417(2)	6202(1)	16(1)
C(22P)	3252(2)	1588(2)	5689(1)	15(1)
N(23P)	4596(2)	1402(2)	6152(1)	19(1)
C(24P)	4698(2)	1067(2)	7212(2)	22(1)
C(25P)	3553(2)	921(2)	7824(2)	21(1)
C(26P)	2264(2)	1094(2)	7269(2)	20(1)

Table S3.3.6.1.3. Bond lengths [Å] and angles [°] for 3a.

Cl(11)-C(12)	1.722(2)
C(11)-C(16)	1.382(3)
C(11)-C(12)	1.391(3)
C(11)-C(112)	1.534(3)
C(12)-C(13)	1.400(3)
C(13)-C(14)	1.386(3)
C(13)-H(13)	0.9500
C(14)-C(15)	1.372(3)
C(14)-H(14)	0.9500
C(15)-C(16)	1.383(3)
C(15)-N(11)	1.470(2)
C(16)-H(16)	0.9500
N(11)-O(11)	1.226(2)
N(11)-O(12)	1.2381(19)
C(112)-O(112)	1.413(2)
C(112)-N(12P)	1.442(2)
C(112)-H(112)	1.0000
O(112)-H(121)	0.872(19)
N(12P)-C(12P)	1.362(2)
N(12P)-H(120)	0.830(19)
N(11P)-C(16P)	1.333(2)
N(11P)-C(12P)	1.350(2)
C(12P)-N(13P)	1.353(2)
N(13P)-C(14P)	1.340(2)
C(14P)-C(15P)	1.375(3)
C(14P)-H(14P)	0.9500
C(15P)-C(16P)	1.377(3)
C(15P)-H(15P)	0.9500
C(16P)-H(16P)	0.9500
Cl(21)-C(22)	1.731(2)
C(21)-C(26)	1.380(3)

Table S3.3.6.1.3. *Cont.*

C(21)-C(22)	1.400(3)
C(21)-C(212)	1.523(3)
C(22)-C(23)	1.396(3)
C(23)-C(24)	1.378(3)
C(23)-H(23)	0.9500
C(24)-C(25)	1.376(3)
C(24)-H(24)	0.9500
C(25)-C(26)	1.383(3)
C(25)-N(21)	1.475(3)
C(26)-H(26)	0.9500
N(21)-O(21)	1.222(2)
N(21)-O(22)	1.230(2)
C(212)-O(212)	1.414(2)
C(212)-N(22P)	1.444(2)
C(212)-H(212)	1.0000
O(212)-H(221)	0.78(2)
N(22P)-C(22P)	1.362(2)
N(22P)-H(220)	0.799(18)
N(21P)-C(22P)	1.344(2)
N(21P)-C(26P)	1.344(2)
C(22P)-N(23P)	1.354(2)
N(23P)-C(24P)	1.331(2)
C(24P)-C(25P)	1.378(3)
C(24P)-H(24P)	0.9500
C(25P)-C(26P)	1.371(3)
C(25P)-H(25P)	0.9500
C(26P)-H(26P)	0.9500
C(16)-C(11)-C(12)	118.24(17)
C(16)-C(11)-C(112)	119.89(17)
C(12)-C(11)-C(112)	121.87(17)
C(11)-C(12)-C(13)	121.68(18)
C(11)-C(12)-Cl(11)	120.50(15)
C(13)-C(12)-Cl(11)	117.82(16)
C(14)-C(13)-C(12)	119.46(19)
C(14)-C(13)-H(13)	120.3
C(12)-C(13)-H(13)	120.3
C(15)-C(14)-C(13)	118.04(19)
C(15)-C(14)-H(14)	121.0
C(13)-C(14)-H(14)	121.0
C(14)-C(15)-C(16)	123.12(19)
C(14)-C(15)-N(11)	118.88(17)
C(16)-C(15)-N(11)	117.99(18)
C(11)-C(16)-C(15)	119.44(18)
C(11)-C(16)-H(16)	120.3
C(15)-C(16)-H(16)	120.3

Table S3.3.6.1.3. *Cont.*

O(11)-N(11)-O(12)	123.49(17)
O(11)-N(11)-C(15)	118.82(16)
O(12)-N(11)-C(15)	117.70(17)
O(112)-C(112)-N(12P)	108.95(17)
O(112)-C(112)-C(11)	110.47(15)
N(12P)-C(112)-C(11)	108.86(16)
O(112)-C(112)-H(112)	109.5
N(12P)-C(112)-H(112)	109.5
C(11)-C(112)-H(112)	109.5
C(112)-O(112)-H(121)	107.0(14)
C(12P)-N(12P)-C(112)	121.47(18)
C(12P)-N(12P)-H(120)	114.7(14)
C(112)-N(12P)-H(120)	120.1(13)
C(16P)-N(11P)-C(12P)	115.05(18)
N(11P)-C(12P)-N(13P)	126.76(17)
N(11P)-C(12P)-N(12P)	117.50(19)
N(13P)-C(12P)-N(12P)	115.71(19)
C(14P)-N(13P)-C(12P)	114.86(18)
N(13P)-C(14P)-C(15P)	123.4(2)
N(13P)-C(14P)-H(14P)	118.3
C(15P)-C(14P)-H(14P)	118.3
C(14P)-C(15P)-C(16P)	116.36(19)
C(14P)-C(15P)-H(15P)	121.8
C(16P)-C(15P)-H(15P)	121.8
N(11P)-C(16P)-C(15P)	123.6(2)
N(11P)-C(16P)-H(16P)	118.2
C(15P)-C(16P)-H(16P)	118.2
C(26)-C(21)-C(22)	117.86(18)
C(26)-C(21)-C(212)	120.31(19)
C(22)-C(21)-C(212)	121.83(18)
C(23)-C(22)-C(21)	121.6(2)
C(23)-C(22)-Cl(21)	118.24(17)
C(21)-C(22)-Cl(21)	120.12(16)
C(24)-C(23)-C(22)	119.5(2)
C(24)-C(23)-H(23)	120.2
C(22)-C(23)-H(23)	120.2
C(25)-C(24)-C(23)	118.65(19)
C(25)-C(24)-H(24)	120.7
C(23)-C(24)-H(24)	120.7
C(24)-C(25)-C(26)	122.4(2)
C(24)-C(25)-N(21)	119.41(18)
C(26)-C(25)-N(21)	118.2(2)
C(21)-C(26)-C(25)	120.0(2)
C(21)-C(26)-H(26)	120.0
C(25)-C(26)-H(26)	120.0

Table S3.3.6.1.3. *Cont.*

O(21)-N(21)-O(22)	123.27(19)
O(21)-N(21)-C(25)	118.93(17)
O(22)-N(21)-C(25)	117.8(2)
O(212)-C(212)-N(22P)	111.56(16)
O(212)-C(212)-C(21)	108.75(16)
N(22P)-C(212)-C(21)	108.41(16)
O(212)-C(212)-H(212)	109.4
N(22P)-C(212)-H(212)	109.4
C(21)-C(212)-H(212)	109.4
C(212)-O(212)-H(221)	106.9(17)
C(22P)-N(22P)-C(212)	122.70(17)
C(22P)-N(22P)-H(220)	116.9(14)
C(212)-N(22P)-H(220)	119.1(14)
C(22P)-N(21P)-C(26P)	115.65(18)
N(21P)-C(22P)-N(23P)	125.95(17)
N(21P)-C(22P)-N(22P)	118.17(19)
N(23P)-C(22P)-N(22P)	115.87(18)
C(24P)-N(23P)-C(22P)	114.88(18)
N(23P)-C(24P)-C(25P)	124.6(2)
N(23P)-C(24P)-H(24P)	117.7
C(25P)-C(24P)-H(24P)	117.7
C(26P)-C(25P)-C(24P)	115.33(18)
C(26P)-C(25P)-H(25P)	122.3
C(24P)-C(25P)-H(25P)	122.3
N(21P)-C(26P)-C(25P)	123.6(2)
N(21P)-C(26P)-H(26P)	118.2
C(25P)-C(26P)-H(26P)	118.2

Symmetry transformations used to generate equivalent atoms.

Table S3.3.6.1.4. Anisotropic displacement parameters (Å^2 × 10^3)for 3a. The anisotropic displacement factor exponent takes the form: $-2\pi^2$ [$h^2a^{*2}U^{11}$ + ... + 2 h k a* b* U^{12}].

	U^{11}	U^{22}	U^{33}	U^{23}	U^{13}	U^{12}
Cl(11)	34(1)	21(1)	22(1)	−3(1)	0(1)	1(1)
C(11)	13(1)	20(1)	12(1)	−6(1)	1(1)	−11(1)
C(12)	16(1)	19(1)	14(1)	−3(1)	−2(1)	−6(1)
C(13)	23(1)	28(1)	21(1)	−14(1)	5(1)	−6(1)
C(14)	23(1)	34(1)	13(1)	−10(1)	4(1)	−12(1)
C(15)	17(1)	22(1)	10(1)	−2(1)	−3(1)	−11(1)
C(16)	12(1)	19(1)	14(1)	−7(1)	1(1)	−8(1)
N(11)	21(1)	26(1)	14(1)	−3(1)	0(1)	−13(1)
O(11)	31(1)	23(1)	17(1)	−4(1)	3(1)	−7(1)
O(12)	34(1)	38(1)	9(1)	−4(1)	3(1)	−19(1)
C(112)	15(1)	16(1)	12(1)	−4(1)	0(1)	−7(1)
O(112)	13(1)	19(1)	16(1)	−6(1)	2(1)	−7(1)

Table S3.3.6.1.4. *Cont.*

	U^{11}	U^{22}	U^{33}	U^{23}	U^{13}	U^{12}
N(12P)	18(1)	22(1)	10(1)	−2(1)	1(1)	−13(1)
N(11P)	19(1)	24(1)	12(1)	−2(1)	3(1)	−12(1)
C(12P)	16(1)	13(1)	12(1)	−2(1)	−1(1)	−1(1)
N(13P)	18(1)	19(1)	15(1)	−6(1)	0(1)	−8(1)
C(14P)	18(1)	30(1)	15(1)	−9(1)	0(1)	−9(1)
C(15P)	28(2)	50(2)	9(1)	−7(1)	4(1)	−23(1)
C(16P)	25(1)	42(2)	14(1)	−6(1)	8(1)	−19(1)
Cl(21)	41(1)	24(1)	23(1)	−12(1)	7(1)	−10(1)
C(21)	12(1)	21(1)	13(1)	−4(1)	−1(1)	−10(1)
C(22)	18(1)	24(1)	18(1)	−11(1)	1(1)	−8(1)
C(23)	29(1)	18(1)	22(1)	−2(1)	−2(1)	−8(1)
C(24)	29(1)	25(1)	14(1)	2(1)	−1(1)	−14(1)
C(25)	19(1)	28(1)	13(1)	−7(1)	0(1)	−11(1)
C(26)	16(1)	17(1)	17(1)	−2(1)	0(1)	−7(1)
N(21)	28(1)	36(1)	14(1)	−6(1)	5(1)	−13(1)
O(21)	35(1)	32(1)	22(1)	−10(1)	7(1)	−9(1)
O(22)	73(1)	47(1)	15(1)	−1(1)	15(1)	−19(1)
C(212)	16(1)	17(1)	11(1)	−5(1)	1(1)	−6(1)
O(212)	24(1)	20(1)	19(1)	−6(1)	6(1)	−14(1)
N(22P)	13(1)	26(1)	11(1)	−5(1)	2(1)	−9(1)
N(21P)	16(1)	21(1)	10(1)	−4(1)	3(1)	−9(1)
C(22P)	18(1)	14(1)	12(1)	−3(1)	0(1)	−6(1
N(23P)	18(1)	26(1)	12(1)	−3(1)	0(1)	−11(1)
C(24P)	23(1)	26(1)	17(1)	−3(1)	−3(1)	−13(1)
C(25P)	24(1)	32(1)	9(1)	−2(1)	−1(1)	−15(1)
C(26P)	20(1)	24(1)	16(1)	−3(1)	6(1)	−11(1)

Table S3.3.6.1.5. Hydrogen coordinates (×10[4]) and isotropic displacement parameters (Å[2] × 10[3]) for 3a.

	x	y	z	U(eq)
H(13)	5692	3777	8354	29
H(14)	6929	1634	9692	27
H(16)	9333	−54	7487	17
H(112)	8466	2992	5406	17
H(121)	10,420(20)	1120(20)	5841(15)	23
H(120)	6530(20)	1740(20)	5366(15)	19
H(14P)	5283	2247	2106	25
H(15P)	6891	3167	1135	32
H(16P)	8641	3535	2075	31
H(23)	376	6528	1488	29
H(24)	1426	5566	121	28
H(26)	2724	1673	2183	20

Table S3.3.6.1.5. *Cont.*

	x	y	z	U(eq)
H(212)	816	2667	4339	17
H(221)	1110(20)	840(20)	4169(16)	29
H(220)	3830(20)	1990(20)	4309(15)	20
H(24P)	5624	919	7571	26
H(25P)	3651	714	8578	25
H(26P)	1455	978	7661	24

Table S3.3.6.1.6. Torsion angles [°] for 3a.

C(16)-C(11)-C(12)-C(13)	−0.8(3)
C(112)-C(11)-C(12)-C(13)	179.50(19)
C(16)-C(11)-C(12)-Cl(11)	179.43(15)
C(112)-C(11)-C(12)-Cl(11)	−0.3(3)
C(11)-C(12)-C(13)-C(14)	1.2(3)
Cl(11)-C(12)-C(13)-C(14)	−179.03(17)
C(12)-C(13)-C(14)-C(15)	-0.5(3)
C(13)-C(14)-C(15)-C(16)	−0.5(3)
C(13)-C(14)-C(15)-N(11)	178.59(18)
C(12)-C(11)-C(16)-C(15)	−0.2(3)
C(112)-C(11)-C(16)-C(15)	179.46(18)
C(14)-C(15)-C(16)-C(11)	0.9(3)
N(11)-C(15)-C(16)-C(11)	−178.20(18)
C(14)-C(15)-N(11)-O(11)	171.3(2)
C(16)-C(15)-N(11)-O(11)	−9.5(3)
C(14)-C(15)-N(11)-O(12)	−8.5(3)
C(16)-C(15)-N(11)-O(12)	170.66(18)
C(16)-C(11)-C(112)-O(112)	−13.4(3)
C(12)-C(11)-C(112)-O(112)	166.26(18)
C(16)-C(11)-C(112)-N(12P)	106.2(2)
C(12)-C(11)-C(112)-N(12P)	−74.1(2)
O(112)-C(112)-N(12P)-C(12P)	−66.3(2)
C(11)-C(112)-N(12P)-C(12P)	173.11(17)
C(16P)-N(11P)-C(12P)-N(13P)	0.4(3)
C(16P)-N(11P)-C(12P)-N(12P)	−177.57(18)
C(112)-N(12P)-C(12P)-N(11P)	−13.7(3)
C(112)-N(12P)-C(12P)-N(13P)	168.15(18)
N(11P)-C(12P)-N(13P)-C(14P)	0.6(3)
N(12P)-C(12P)-N(13P)-C(14P)	178.59(17)
C(12P)-N(13P)-C(14P)-C(15P)	−1.5(3)
N(13P)-C(14P)-C(15P)-C(16P)	1.4(3)
C(12P)-N(11P)-C(16P)-C(15P)	−0.5(3)
C(14P)-C(15P)-C(16P)-N(11P)	−0.3(3)
C(26)-C(21)-C(22)-C(23)	0.6(3)
C(212)-C(21)-C(22)-C(23)	−179.12(19)
C(26)-C(21)-C(22)-Cl(21)	179.33(16)

Table S3.3.6.1.6. *Cont.*

C(212)-C(21)-C(22)-Cl(21)	−0.4(3)
C(21)-C(22)-C(23)-C(24)	−1.0(3)
Cl(21)-C(22)-C(23)-C(24)	−179.68(17)
C(22)-C(23)-C(24)-C(25)	0.1(3)
C(23)-C(24)-C(25)-C(26)	1.2(3)
C(23)-C(24)-C(25)-N(21)	−177.6(2)
C(22)-C(21)-C(26)-C(25)	0.6(3)
C(212)-C(21)-C(26)-C(25)	−179.66(18)
C(24)-C(25)-C(26)-C(21)	−1.5(3)
N(21)-C(25)-C(26)-C(21)	177.28(19)
C(24)-C(25)-N(21)-O(21)	179.8(2)
C(26)-C(25)-N(21)-O(21)	0.9(3)
C(24)-C(25)-N(21)-O(22)	0.3(3)
C(26)-C(25)-N(21)-O(22)	−178.51(19)
C(26)-C(21)-C(212)-O(212)	−27.5(2)
C(22)-C(21)-C(212)-O(212)	152.29(18)
C(26)-C(21)-C(212)-N(22P)	94.0(2)
C(22)-C(21)-C(212)-N(22P)	−86.2(2)
O(212)-C(212)-N(22P)-C(22P)	−81.7(2)
C(21)-C(212)-N(22P)-C(22P)	158.58(18)
C(26P)-N(21P)-C(22P)-N(23P)	3.2(3)
C(26P)-N(21P)-C(22P)-N(22P)	−175.34(18)
C(212)-N(22P)-C(22P)-N(21P)	−3.0(3)
C(212)-N(22P)-C(22P)-N(23P)	178.29(18)
N(21P)-C(22P)-N(23P)-C(24P)	−2.5(3)
N(22P)-C(22P)-N(23P)-C(24P)	176.07(18)
C(22P)-N(23P)-C(24P)-C(25P)	−0.5(3)
N(23P)-C(24P)-C(25P)-C(26P)	2.4(3)
C(22P)-N(21P)-C(26P)-C(25P)	−1.0(3)
C(24P)-C(25P)-C(26P)-N(21P)	−1.6(3)

Symmetry transformations used to generate equivalent atoms.

Table S3.3.6.1.7. Hydrogen bonds for 3a [Å and °].

D-H...A	d(D-H)	d(H...A)	d(D...A)	<(DHA)
O(112)-H(121)...N(21P)#1	0.872(19)	1.85(2)	2.717(2)	178(2)
O(212)-H(221)...O(112)#2	0.78(2)	2.41(2)	3.083(2)	146(2)
O(212)-H(221)...O(112)#3	0.78(2)	2.29(2)	2.845(2)	129(2)
N(12P)-H(120)...N(23P)	0.830(19)	2.206(19)	2.999(3)	159.8(19)
N(22P)-H(220)...N(13P)	0.799(18)	2.338(19)	3.133(3)	172.9(19)
C(26P)-H(26P)...O(12)#4	0.95	2.43	3.118(3)	128.9

Symmetry transformations used to generate equivalent atoms: #1 x+1,y,z #2 x-1,y,z #3 -x+1,-y,-z+1 #4 -x+1,-y,-z+2 .

3.3.6.2. (2-chloro-5-nitrophenyl)(pyrimidin-2-ylamino)methanol (3b)

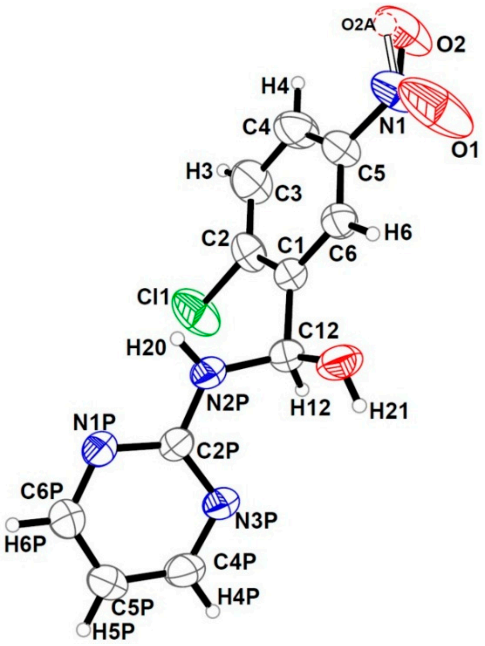

Figure 4. Molecular structure and labelling for (2-chloro-5-nitrophenyl)(pyrimidin-2-ylamino)methanol (3b). Displacement ellipsoids are shown at the 50% probability level.

Table S3.3.6.2.1. Crystal data and structure refinement for 3b.

Identification Code	3b	
Empirical formula	C11 H9 Cl N4 O3	
Formula weight	280.67	
Temperature	296(2) K	
Wavelength	0.71073 Å	
Crystal system	Monoclinic	
Space group	P 1 21/c 1	
Unit cell dimensions	a = 14.841(4) Å	α = 90°.
	b = 9.391(3) Å	β = 101.91(3)°
	c = 9.029(3) Å	γ = 90°
Volume	1231.3(7) $Å^3$	
Z	4	
Density (calculated)	1.514 Mg/m^3	
Absorption coefficient	0.320 mm^{-1}	
F(000)	576	
Crystal size	0.25 × 0.10 × 0.06 mm^3	
Theta range for data collection	2.81 to 28.77°	
Index ranges	$-18 \leq h \leq 13, -12 \leq k \leq 7, -11 \leq l \leq 9$	
Reflections collected	5336	
Independent reflections	2805 [R(int) = 0.0224]	
Completeness to theta = 27.00°	98.3%	
Absorption correction	Semi-empirical from equivalents	

Table S3.3.6.2.1. *Cont.*

Identification Code	3b
Max. and min. transmission	1.00000 and 0.97010
Refinement method	Full-matrix least-squares on F^2
Data/restraints/parameters	2805/0/197
Goodness-of-fit on F^2	0.999
Final R indices [I > 2sigma(I)]	R1 = 0.0539, wR2 = 0.1065
R indices (all data)	R1 = 0.0991, wR2 = 0.1264
Largest diff. peak and hole	0.275 and −0.336 e.Å^{-3}

Table S3.3.6.2.2. Atomic coordinates (×10^4) and equivalent isotropic displacement parameters (Å^2 × 10^3) for 3b. U(eq) is defined as one third of the trace of the orthogonalized U^{ij} tensor.

	x	y	z	U(eq)
Cl(1)	3813(1)	7931(1)	4971(1)	78(1)
C(1)	2920(2)	5458(2)	4071(2)	39(1)
C(2)	3636(2)	6119(3)	5069(3)	49(1)
C(3)	4236(2)	5357(3)	6166(3)	65(1)
C(4)	4159(2)	3910(3)	6257(3)	61(1)
C(5)	3474(2)	3254(3)	5236(3)	48(1)
C(6)	2860(2)	3994(3)	4162(2)	43(1)
N(1)	3408(2)	1694(3)	5253(3)	66(1)
O(1)	2761(19)	1170(20)	4430(30)	119(6)
O(2)	3976(17)	1002(19)	6110(30)	98(5)
O(1A)	2982(17)	1060(20)	4109(17)	79(5)
O(2A)	3855(16)	1120(20)	6420(20)	71(4)
C(12)	2223(2)	6276(3)	2908(2)	43(1)
O(12)	1601(1)	5290(2)	2087(2)	60(1)
N(2P)	1758(2)	7302(2)	3670(2)	46(1)
N(1P)	995(1)	9358(2)	3971(2)	45(1)
C(2P)	1386(2)	8530(3)	3059(2)	37(1)
N(3P)	1429(1)	8844(2)	1619(2)	45(1)
C(4P)	1089(2)	10,111(3)	1130(3)	52(1)
C(5P)	702(2)	11,052(3)	1973(3)	58(1)
C(6P)	660(2)	10,597(3)	3403(3)	54(1)

Table S3.3.6.2.3. Bond lengths [Å] and angles [°] for 3b.

Cl(1)-C(2)	1.727(3)
C(1)-C(6)	1.381(3)
C(1)-C(2)	1.390(3)
C(1)-C(12)	1.521(3)
C(2)-C(3)	1.387(4)
C(3)-C(4)	1.367(4)
C(3)-H(3)	0.9300

Table S3.3.6.2.3. *Cont.*

C(4)-C(5)	1.370(3)
C(4)-H(4)	0.9300
C(5)-C(6)	1.375(3)
C(5)-N(1)	1.469(3)
C(6)-H(6)	0.9300
N(1)-O(2)	1.207(17)
N(1)-O(1A)	1.244(15)
N(1)-O(1)	1.192(15)
N(1)-O(2A)	1.25(2)
C(12)-O(12)	1.406(3)
C(12)-N(2P)	1.440(3)
C(12)-H(12)	0.9800
O(12)-H(21)	0.80(3)
N(2P)-C(2P)	1.347(3)
N(2P)-H(20)	0.83(3)
N(1P)-C(6P)	1.326(3)
N(1P)-C(2P)	1.347(3)
C(2P)-N(3P)	1.347(3)
N(3P)-C(4P)	1.332(3)
C(4P)-C(5P)	1.367(4)
C(4P)-H(4P)	0.9300
C(5P)-C(6P)	1.374(4)
C(5P)-H(5P)	0.9300
C(6P)-H(6P)	0.9300
C(6)-C(1)-C(2)	117.2(2)
C(6)-C(1)-C(12)	120.0(2)
C(2)-C(1)-C(12)	122.8(2)
C(3)-C(2)-C(1)	121.7(2)
C(3)-C(2)-Cl(1)	117.6(2)
C(1)-C(2)-Cl(1)	120.75(19)
C(4)-C(3)-C(2)	120.5(2)
C(4)-C(3)-H(3)	119.8
C(2)-C(3)-H(3)	119.8
C(3)-C(4)-C(5)	117.8(2)
C(3)-C(4)-H(4)	121.1
C(5)-C(4)-H(4)	121.1
C(4)-C(5)-C(6)	122.6(2)
C(4)-C(5)-N(1)	118.9(2)
C(6)-C(5)-N(1)	118.4(2)
C(5)-C(6)-C(1)	120.2(2)
C(5)-C(6)-H(6)	119.9
C(1)-C(6)-H(6)	119.9
O(2)-N(1)-O(1A)	116.3(13)
O(2)-N(1)-O(1)	122.8(12)
O(1A)-N(1)-O(2A)	125.9(15)

Table S3.3.6.2.3. *Cont.*

O(1)-N(1)-O(2A)	124.5(14)
O(2)-N(1)-C(5)	120.3(8)
O(1A)-N(1)-C(5)	119.3(9)
O(1)-N(1)-C(5)	116.8(9)
O(2A)-N(1)-C(5)	114.6(12)
O(12)-C(12)-N(2P)	111.4(2)
O(12)-C(12)-C(1)	108.08(19)
N(2P)-C(12)-C(1)	109.42(17)
O(12)-C(12)-H(12)	109.3
N(2P)-C(12)-H(12)	109.3
C(1)-C(12)-H(12)	109.3
C(12)-O(12)-H(21)	109(2)
C(2P)-N(2P)-C(12)	125.02(19)
C(2P)-N(2P)-H(20)	113.0(19)
C(12)-N(2P)-H(20)	121.9(19)
C(6P)-N(1P)-C(2P)	116.4(2)
N(3P)-C(2P)-N(2P)	119.0(2)
N(3P)-C(2P)-N(1P)	125.1(2)
N(2P)-C(2P)-N(1P)	115.82(18)
C(4P)-N(3P)-C(2P)	115.2(2)
N(3P)-C(4P)-C(5P)	124.5(2)
N(3P)-C(4P)-H(4P)	117.7
C(5P)-C(4P)-H(4P)	117.7
C(4P)-C(5P)-C(6P)	115.4(2)
C(4P)-C(5P)-H(5P)	122.3
C(6P)-C(5P)-H(5P)	122.3
N(1P)-C(6P)-C(5P)	123.3(2)
N(1P)-C(6P)-H(6P)	118.3
C(5P)-C(6P)-H(6P)	118.3

Symmetry transformations used to generate equivalent atoms.

Table S3.3.6.2.4. Anisotropic displacement parameters ($Å^2 \times 10^3$)for 3b. The anisotropic displacement factor exponent takes the form: $-2\pi^2[\ h^2a^{*2}U^{11} + ... + 2\ h\ k\ a^*\ b^*\ U^{12}]$.

	U^{11}	U^{22}	U^{33}	U^{23}	U^{13}	U^{12}
Cl(1)	75(1)	38(1)	115(1)	−6(1)	4(1)	−8(1)
C(1)	47(1)	37(1)	35(1)	1(1)	11(1)	3(1)
C(2)	52(2)	35(1)	60(2)	−6(1)	11(1)	−1(1)
C(3)	58(2)	55(2)	71(2)	−12(1)	−13(1)	−1(2)
C(4)	55(2)	54(2)	64(2)	5(1)	−10(1)	9(1)
C(5)	50(2)	37(1)	55(1)	3(1)	9(1)	4(1)
C(6)	45(1)	41(1)	42(1)	−1(1)	4(1)	2(1)
N(1)	56(2)	45(1)	90(2)	15(1)	0(1)	4(1)
O(1)	80(8)	46(6)	204(12)	15(7)	−36(8)	−13(5)
O(2)	103(9)	42(4)	126(10)	2(5)	−30(7)	9(5)

Table S3.3.6.2.4. *Cont.*

	U^{11}	U^{22}	U^{33}	U^{23}	U^{13}	U^{12}
O(1A)	102(11)	41(4)	75(9)	−16(4)	−25(5)	13(6)
O(2A)	80(6)	59(8)	74(6)	38(7)	13(5)	26(5)
C(12)	56(2)	38(1)	35(1)	4(1)	12(1)	3(1)
O(12)	79(1)	54(1)	38(1)	7(1)	−9(1)	−4(1)
N(2P)	61(1)	49(1)	32(1)	9(1)	17(1)	13(1)
N(1P)	49(1)	52(1)	33(1)	0(1)	8(1)	10(1)
C(2P)	38(1)	43(1)	30(1)	1(1)	7(1)	0(1)
N(3P)	55(1)	48(1)	32(1)	7(1)	11(1)	9(1)
C(4P)	60(2)	60(2)	39(1)	13(1)	12(1)	12(1)
C(5P)	68(2)	54(2)	52(2)	11(1)	14(1)	24(2)
C(6P)	58(2)	57(2)	46(1)	−2(1)	12(1)	18(1)

Table S3.3.6.2.5. Hydrogen coordinates (×10^4) and isotropic displacement parameters (Å^2 × 10^3) for 3b.

	x	y	z	U(eq)
H(3)	4693	5833	6845	78
H(4)	4559	3389	6987	73
H(6)	2402	3508	3494	52
H(12)	2545	6773	2215	51
H(21)	1460(20)	5550(40)	1230(40)	90
H(20)	1688(18)	7180(30)	4550(30)	55
H(4P)	1116	10374	147	63
H(5P)	483	11939	1604	70
H(6P)	381	11188	4004	64

Table S3.3.6.2.6. Torsion angles [°] for 3b.

C(6)-C(1)-C(2)-C(3)	−3.4(4)
C(12)-C(1)-C(2)-C(3)	176.9(2)
C(6)-C(1)-C(2)-Cl(1)	176.05(19)
C(12)-C(1)-C(2)-Cl(1)	−3.6(3)
C(1)-C(2)-C(3)-C(4)	2.6(4)
Cl(1)-C(2)-C(3)-C(4)	−176.9(2)
C(2)-C(3)-C(4)-C(5)	−0.1(5)
C(3)-C(4)-C(5)-C(6)	−1.3(4)
C(3)-C(4)-C(5)-N(1)	176.6(3)
C(4)-C(5)-C(6)-C(1)	0.4(4)
N(1)-C(5)-C(6)-C(1)	−177.5(2)
C(2)-C(1)-C(6)-C(5)	1.9(4)
C(12)-C(1)-C(6)-C(5)	−178.4(2)
C(4)-C(5)-N(1)-O(2)	−3(2)
C(6)-C(5)-N(1)-O(2)	175(2)
C(4)-C(5)-N(1)-O(1A)	−159.7(14)

Table S3.3.6.2.6. *Cont.*

C(6)-C(5)-N(1)-O(1A)	18.4(14)
C(4)-C(5)-N(1)-O(1)	174(2)
C(6)-C(5)-N(1)-O(1)	−8(2)
C(4)-C(5)-N(1)-O(2A)	15.7(11)
C(6)-C(5)-N(1)-O(2A)	−166.2(11)
C(6)-C(1)-C(12)-O(12)	0.1(3)
C(2)-C(1)-C(12)-O(12)	179.8(2)
C(6)-C(1)-C(12)-N(2P)	121.6(2)
C(2)-C(1)-C(12)-N(2P)	−58.7(3)
O(12)-C(12)-N(2P)-C(2P)	−87.7(3)
C(1)-C(12)-N(2P)-C(2P)	152.9(2)
C(12)-N(2P)-C(2P)-N(3P)	1.0(4)
C(12)-N(2P)-C(2P)-N(1P)	−178.9(2)
C(6P)-N(1P)-C(2P)-N(3P)	−2.5(3)
C(6P)-N(1P)-C(2P)-N(2P)	177.3(2)
N(2P)-C(2P)-N(3P)-C(4P)	−176.6(2)
N(1P)-C(2P)-N(3P)-C(4P)	3.2(3)
C(2P)-N(3P)-C(4P)-C(5P)	−1.1(4)
N(3P)-C(4P)-C(5P)-C(6P)	−1.5(4)
C(2P)-N(1P)-C(6P)-C(5P)	−0.5(4)
C(4P)-C(5P)-C(6P)-N(1P)	2.3(4)

Symmetry transformations used to generate equivalent atoms.

Table S3.3.6.2.7. Hydrogen bonds for 3b [Å and °].

D-H...A	d(D-H)	d(H...A)	d(D...A)	<(DHA)
O(12)-H(21)...N(1P)#1	0.80(3)	2.01(3)	2.791(3)	164(3)
N(2P)-H(20)...N(3P)#2	0.83(3)	2.21(3)	3.004(3)	162(3)
C(3)-H(3)...O(2)#3	0.93	2.42	3.283(19)	155.0
C(3)-H(3)...O(2A)#3	0.93	2.40	3.28(2)	156.6

Symmetry transformations used to generate equivalent atoms: #1 x,-y+3/2,z-1/2 #2 x,-y+3/2,z+1/2 #3 -x+1,y+1/2,-z+3/2

3.4. (5-Chloro-2-nitrophenyl)(pyrimidin-2-ylamino)methanol (4)

3.4.1. Synthesis

Acetonitrilic solution (3 mL) of 5-chloro-2-nitrobenzaldehyde (41 mg) was added to an acetonitrilic solution (3 mL) of 2-aminopyrimidine (21 mg). The reaction mixture after complete dissolution was stirred for 2 hours at 50 °C. The title compound crystallised directly from the mother liquor. Upon standing 3 days at the room temperature, the solution deposited colourless crystal blocks. The crystals were filtered off, washed with a small amount of acetonitrile and diethyl ether then dried in the air to afford (5-chloro-2-nitrophenyl)(pyrimidin-2-ylamino)methanol—(53 mg, 85%), mp 114–115 °C.

3.4.2. Elemental Analysis

	% C	% H	% N
Calculated	47.07	3.23	19.96
Found	46.87	3.18	20.15

3.4.3. Mass Spectrometry

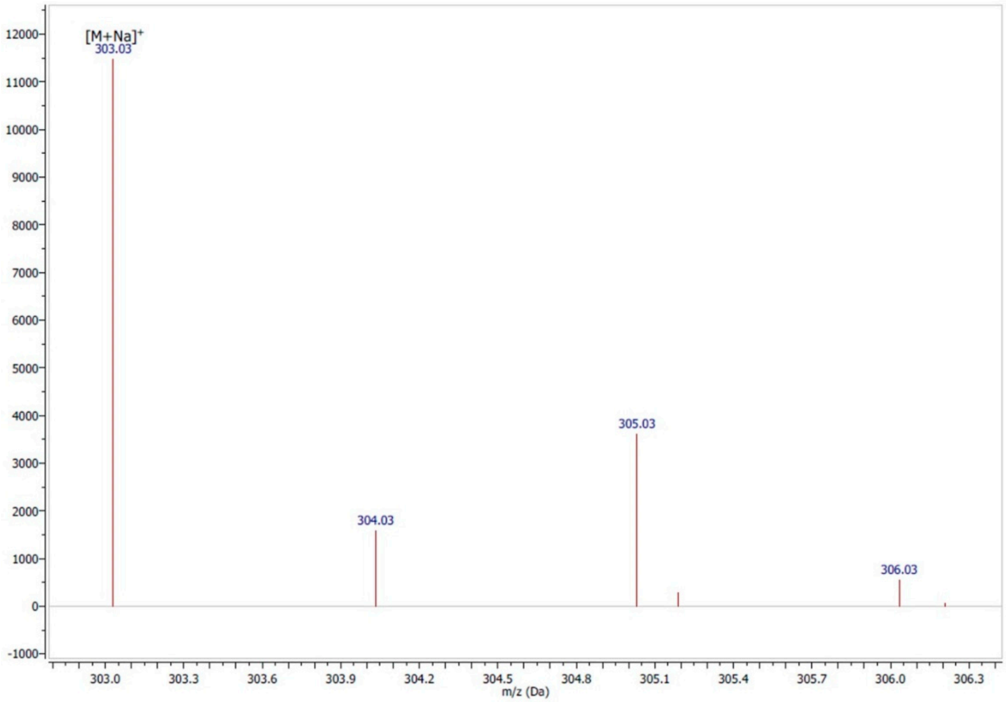

3.4.4. NMR Spectroscopy

^{1}H-NMR

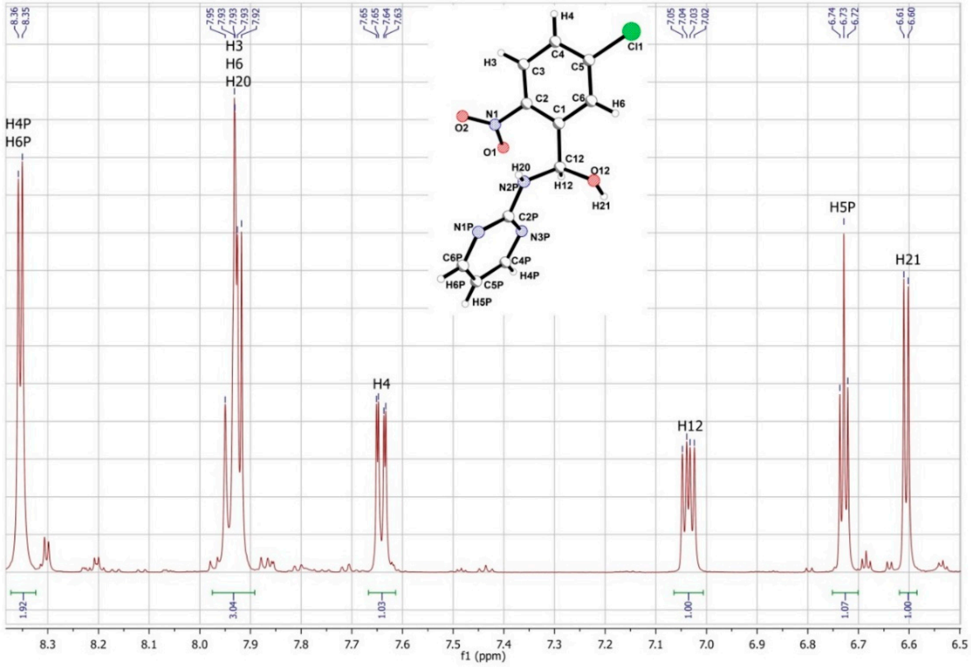

^{1}H-NMR (600 MHz, DMSO, RT) δ: 8.35 (d, $^3J_{H4P/H6P,H5P}$ = 4.8 Hz, 2H, H4P,H6P), 7.92–7.95 (m, 3H, H3,H6,H20), 7.64 (dd, $^3J_{H4,H3}$ = 8.4 Hz, $^4J_{H4,H6}$ = 2.4 Hz, 1H,H4), 7.02–7.05 (m, 1H, H12), 6.73 (t, $^3J_{H5P,H4P/H6P}$ = 4.8 Hz, 1H. H5P), 6.61(d, $^3J_{H21,H12}$ = 4.8 Hz, 1H, H21).

^{13}C-NMR

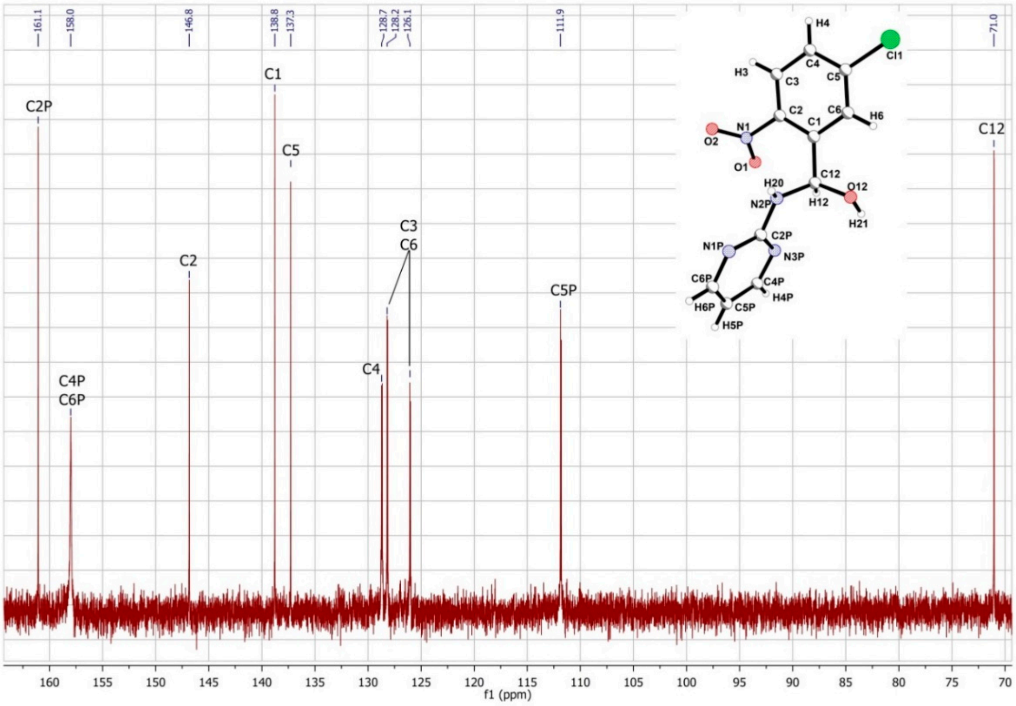

^{13}C-NMR (150.9 MHz, DMSO, RT) δ: 161.1 (C2P), 158.0 (C4P, C6P), 146.8 (C2), 138.8 (C1), 137.3 (C5), 128.7 (C4), 128.2 and 126.1 (C3, C6), 111.9(C5P), 71.0 (C12).

COSY

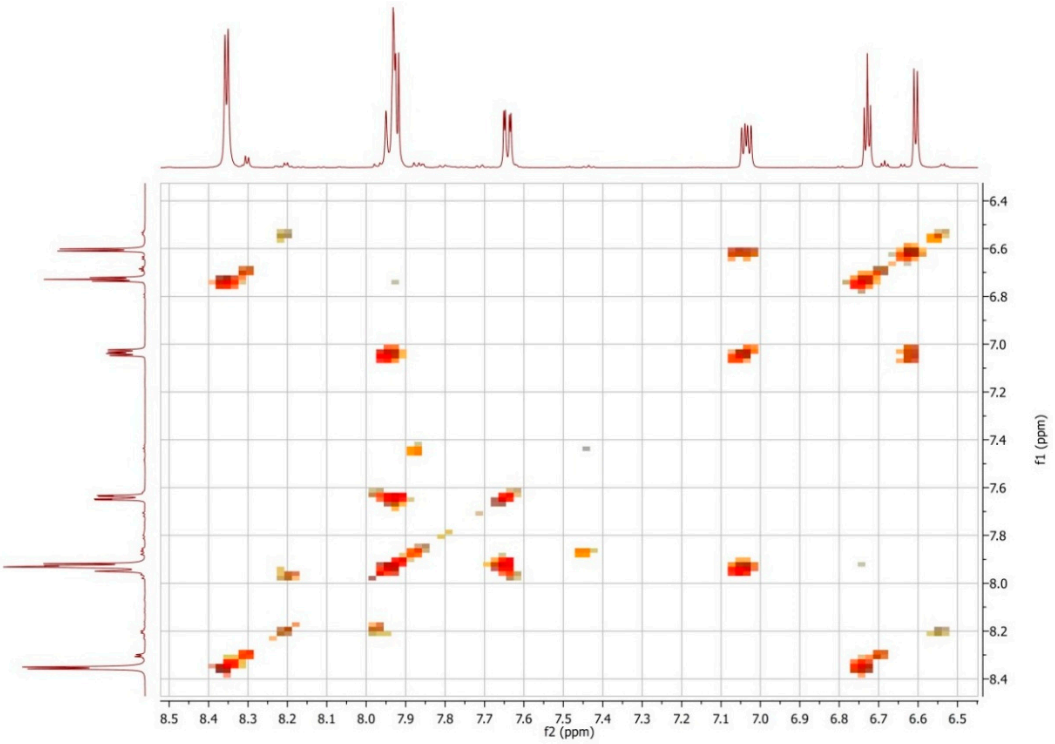

HMQC

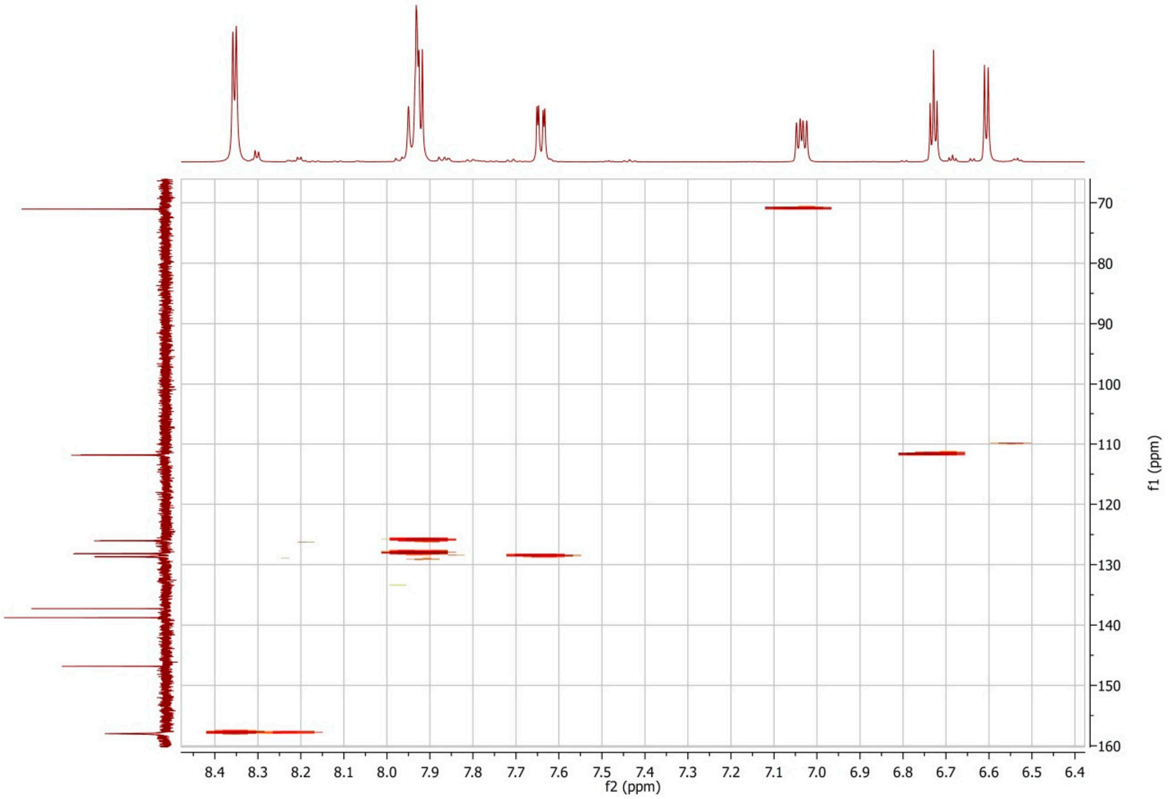

HMBC

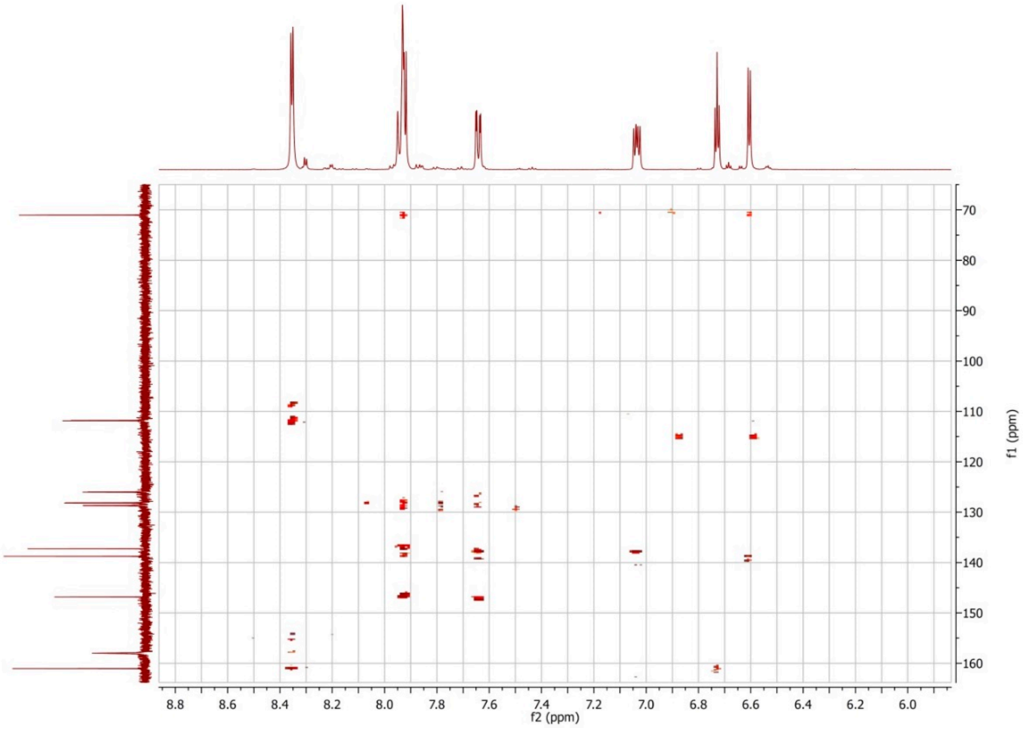

3.4.5. IR Spectroscopy

IR (KBr, cm^{-1}): 3319 s, 3101 m, 3061 m, 2959 m, 2833 m, 2703 m, 1653 w, 1589 vs, 1573 vs, 1520 vs, 1463 vs, 1423 s, 1398 m, 1367 vs, 1299 m, 1252 s, 1225 w, 1195 m, 1146 w, 1114 m, 1104 m, 1084 s,

1070 s, 1032 s, 1000 m, 953 vw, 935 w, 912 vw, 888 w, 845 m, 828 m, 803 m, 758 m, 693 w, 648 m, 601 w, 568 m, 518 w, 440 w.

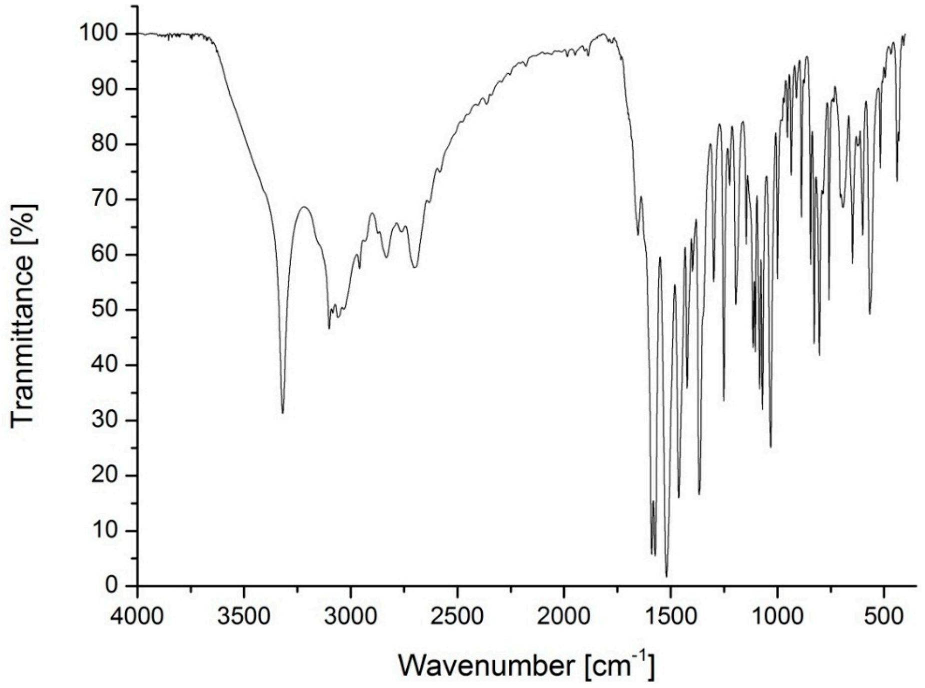

3.4.6. Crystallography

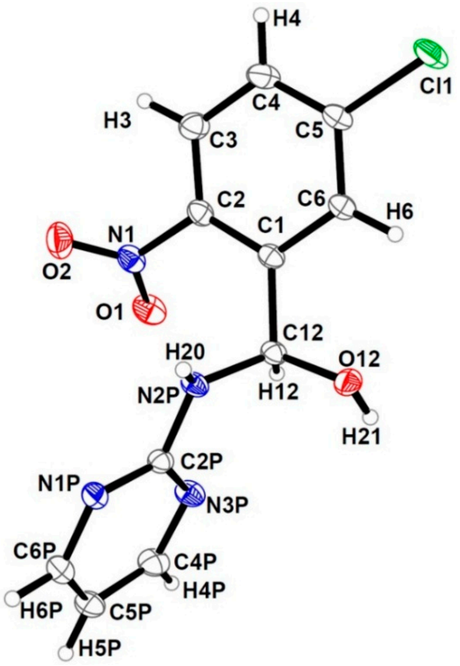

Figure 5. Molecular structure and labelling for (5-chloro-2-nitrophenyl)(pyrimidin-2-ylamino)methanol (4). Displacement ellipsoids are shown at the 50% probability level.

Table S3.4.6.1. Crystal data and structure refinement for 4.

**Identification Code**	**4**	
Empirical formula	C11 H9 Cl N4 O3	Empirical formula
Formula weight	280.67	
Temperature	100(2) K	
Wavelength	0.71073 Å	
Crystal system	Monoclinic	
Space group	P 1 21/n 1	
Unit cell dimensions	a = 11.353(4) Å	α = 90°
	b = 5.441(2) Å	β = 90.29(3)°.
	c = 19.095(6) Å	γ = 90°
Volume	1179.5(7) $Å^3$	
Z	4	
Density (calculated)	1.581 Mg/m^3	
Absorption coefficient	0.334 mm^{-1}	
F(000)	576	
Crystal size	0.36 × 0.26 × 0.24 mm^3	
Theta range for data collection	3.59 to 36.91°	
Index ranges	$-18 \le h \le 18$, $-7 \le k \le 9$, $-32 \le l \le 31$	
Reflections collected	20358	
Independent reflections	5665 [R(int) = 0.0358]	
Completeness to theta = 27.00°	99.9 %	
Absorption correction	Semi-empirical from equivalents	
Max. and min. transmission	1.00000 and 0.93380	
Refinement method	Full-matrix least-squares on F^2	
Data/restraints/parameters	5665/0/178	
Goodness-of-fit on F^2	0.950	
Final R indices [I > 2sigma(I)]	R1 = 0.0465, wR2 = 0.1116	
R indices (all data)	R1 = 0.0730, wR2 = 0.1201	
Largest diff. peak and hole	0.656 and −0.221 e.$Å^{-3}$	

Table S3.4.6.2. Atomic coordinates ($\times 10^4$) and equivalent isotropic displacement parameters ($Å^2 \times 10^3$) for 4. U(eq) is defined as one third of the trace of the orthogonalized U^{ij} tensor.

	**x**	**y**	**z**	**U(eq)**
Cl(1)	6450(1)	8777(1)	7697(1)	27(1)
C(1)	3708(1)	5357(2)	8569(1)	16(1)
C(2)	4305(1)	3741(2)	9013(1)	18(1)
C(3)	5516(1)	3532(2)	9040(1)	23(1)
C(4)	6192(1)	5083(2)	8622(1)	23(1)
C(5)	5618(1)	6765(2)	8199(1)	19(1)
C(6)	4395(1)	6912(2)	8162(1)	18(1)
N(1)	3642(1)	2234(2)	9517(1)	21(1)
O(1)	2903(1)	3284(2)	9876(1)	26(1)
O(2)	3888(1)	46(2)	9566(1)	30(1)

Table S3.4.6.2. *Cont.*

	x	y	z	U(eq)
C(12)	2369(1)	5409(2)	8515(1)	16(1)
O(12)	2020(1)	6809(2)	7925(1)	20(1)
N(2P)	1948(1)	2909(2)	8463(1)	16(1)
N(1P)	467(1)	19(2)	8492(1)	20(1)
C(2P)	865(1)	2231(2)	8708(1)	16(1)
N(3P)	310(1)	3744(2)	9149(1)	20(1)
C(4P)	−727(1)	2945(2)	9396(1)	23(1)
C(5P)	−1221(1)	730(2)	9203(1)	25(1)
C(6P)	−580(1)	-683(2)	8740(1)	25(1)

Table S3.4.6.3. Bond lengths [Å] and angles [°] for 4.

Cl(1)-C(5)	1.7376(11)
C(1)-C(6)	1.3918(15)
C(1)-C(2)	1.3942(15)
C(1)-C(12)	1.5234(15)
C(2)-C(3)	1.3809(16)
C(2)-N(1)	1.4741(14)
C(3)-C(4)	1.3951(16)
C(3)-H(3)	0.9500
C(4)-C(5)	1.3812(17)
C(4)-H(4)	0.9500
C(5)-C(6)	1.3920(15)
C(6)-H(6)	0.9500
N(1)-O(2)	1.2260(14)
N(1)-O(1)	1.2269(13)
C(12)-O(12)	1.4138(13)
C(12)-N(2P)	1.4454(15)
C(12)-H(12)	1.0000
O(12)-H(21)	0.803(17)
N(2P)-C(2P)	1.3677(13)
N(2P)-H(20)	0.812(15)
N(1P)-C(6P)	1.3381(15)
N(1P)-C(2P)	1.3496(15)
C(2P)-N(3P)	1.3374(14)
N(3P)-C(4P)	1.3421(14)
C(4P)-C(5P)	1.3787(18)
C(4P)-H(4P)	0.9500
C(5P)-C(6P)	1.3820(18)
C(5P)-H(5P)	0.9500
C(6P)-H(6P)	0.9500
C(6)-C(1)-C(2)	116.79(9)
C(6)-C(1)-C(12)	120.85(9)
C(2)-C(1)-C(12)	122.35(9)

Table S3.4.6.3. *Cont.*

C(3)-C(2)-C(1)	123.79(10)
C(3)-C(2)-N(1)	116.17(10)
C(1)-C(2)-N(1)	119.96(9)
C(2)-C(3)-C(4)	118.63(10)
C(2)-C(3)-H(3)	120.7
C(4)-C(3)-H(3)	120.7
C(5)-C(4)-C(3)	118.42(10)
C(5)-C(4)-H(4)	120.8
C(3)-C(4)-H(4)	120.8
C(4)-C(5)-C(6)	122.40(10)
C(4)-C(5)-Cl(1)	118.90(8)
C(6)-C(5)-Cl(1)	118.69(9)
C(1)-C(6)-C(5)	119.88(10)
C(1)-C(6)-H(6)	120.1
C(5)-C(6)-H(6)	120.1
O(2)-N(1)-O(1)	124.46(10)
O(2)-N(1)-C(2)	118.23(9)
O(1)-N(1)-C(2)	117.28(10)
O(12)-C(12)-N(2P)	111.19(9)
O(12)-C(12)-C(1)	109.87(8)
N(2P)-C(12)-C(1)	108.48(8)
O(12)-C(12)-H(12)	109.1
N(2P)-C(12)-H(12)	109.1
C(1)-C(12)-H(12)	109.1
C(12)-O(12)-H(21)	107.4(12)
C(2P)-N(2P)-C(12)	121.87(9)
C(2P)-N(2P)-H(20)	114.9(10)
C(12)-N(2P)-H(20)	116.4(11)
C(6P)-N(1P)-C(2P)	116.28(9)
N(3P)-C(2P)-N(1P)	125.70(9)
N(3P)-C(2P)-N(2P)	118.45(10)
N(1P)-C(2P)-N(2P)	115.82(9)
C(2P)-N(3P)-C(4P)	116.01(10)
N(3P)-C(4P)-C(5P)	123.11(10)
N(3P)-C(4P)-H(4P)	118.4
C(5P)-C(4P)-H(4P)	118.4
C(4P)-C(5P)-C(6P)	116.23(10)
C(4P)-C(5P)-H(5P)	121.9
C(6P)-C(5P)-H(5P)	121.9
N(1P)-C(6P)-C(5P)	122.66(11)
N(1P)-C(6P)-H(6P)	118.7
C(5P)-C(6P)-H(6P)	118.7

Symmetry transformations used to generate equivalent atoms.

Table S3.4.6.4. Anisotropic displacement parameters ($Å^2 \times 10^3$)for 4. The anisotropic displacement factor exponent takes the form: $-2\pi^2$ [$h^2a^{*2}U^{11}$ + ... + 2 h k a* b* U^{12}].

	U^{11}	U^{22}	U^{33}	U^{23}	U^{13}	U^{12}
Cl(1)	20(1)	34(1)	27(1)	5(1)	6(1)	−9(1)
C(1)	16(1)	17(1)	15(1)	−2(1)	3(1)	−2(1)
C(2)	18(1)	20(1)	14(1)	2(1)	1(1)	−3(1)
C(3)	19(1)	29(1)	20(1)	4(1)	−1(1)	0(1)
C(4)	15(1)	32(1)	22(1)	2(1)	0(1)	−3(1)
C(5)	17(1)	24(1)	17(1)	0(1)	3(1)	−5(1)
C(6)	16(1)	20(1)	16(1)	0(1)	3(1)	−3(1)
N(1)	21(1)	24(1)	16(1)	3(1)	−1(1)	−5(1)
O(1)	26(1)	35(1)	17(1)	1(1)	6(1)	−5(1)
O(2)	36(1)	23(1)	30(1)	9(1)	−2(1)	−3(1)
C(12)	15(1)	18(1)	17(1)	0(1)	4(1)	−1(1)
O(12)	17(1)	22(1)	20(1)	4(1)	4(1)	4(1)
N(2P)	15(1)	18(1)	17(1)	−2(1)	4(1)	−2(1)
N(1P)	17(1)	17(1)	27(1)	2(1)	3(1)	−1(1)
C(2P)	14(1)	19(1)	16(1)	2(1)	2(1)	0(1)
N(3P)	17(1)	24(1)	20(1)	−2(1)	7(1)	−1(1)
C(4P)	19(1)	30(1)	22(1)	1(1)	7(1)	0(1)
C(5P)	16(1)	29(1)	31(1)	7(1)	6(1)	−3(1)
C(6P)	19(1)	20(1)	36(1)	4(1)	2(1)	−3(1)

Table S3.4.6.5. Hydrogen coordinates ($\times 10^4$) and isotropic displacement parameters ($Å^2 \times 10^3$) for 4.

	x	y	z	U(eq)
H(3)	5882	2357	9338	27
H(4)	7028	4984	8627	28
H(6)	4031	8071	7858	21
H(12)	2041	6181	8947	20
H(21)	1522(15)	7750(30)	8053(8)	30
H(20)	2182(13)	2110(30)	8132(8)	20
H(4P)	−1139	3954	9718	28
H(5P)	−1961	205	9379	30
H(6P)	−896	−2216	8592	30

Table S3.4.6.6. Torsion angles [°] for 4.

C(6)-C(1)-C(2)-C(3)	−3.28(16)
C(12)-C(1)-C(2)-C(3)	175.49(10)
C(6)-C(1)-C(2)-N(1)	173.21(9)
C(12)-C(1)-C(2)-N(1)	−8.02(15)
C(1)-C(2)-C(3)-C(4)	2.53(17)
N(1)-C(2)-C(3)-C(4)	−174.08(10)
C(2)-C(3)-C(4)-C(5)	0.19(17)
C(3)-C(4)-C(5)-C(6)	−2.02(17)
C(3)-C(4)-C(5)-Cl(1)	177.87(9)
C(2)-C(1)-C(6)-C(5)	1.36(15)
C(12)-C(1)-C(6)-C(5)	−177.43(9)
C(4)-C(5)-C(6)-C(1)	1.22(16)
Cl(1)-C(5)-C(6)-C(1)	−178.67(8)
C(3)-C(2)-N(1)-O(2)	−49.09(14)
C(1)-C(2)-N(1)-O(2)	134.16(11)
C(3)-C(2)-N(1)-O(1)	128.68(11)
C(1)-C(2)-N(1)-O(1)	−48.07(14)
C(6)-C(1)-C(12)-O(12)	11.52(13)
C(2)-C(1)-C(12)-O(12)	−167.21(9)
C(6)-C(1)-C(12)-N(2P)	133.25(10)
C(2)-C(1)-C(12)-N(2P)	−45.47(13)
O(12)-C(12)-N(2P)-C(2P)	−88.38(11)
C(1)-C(12)-N(2P)-C(2P)	150.70(9)
C(6P)-N(1P)-C(2P)-N(3P)	0.62(16)
C(6P)-N(1P)-C(2P)-N(2P)	178.63(10)
C(12)-N(2P)-C(2P)-N(3P)	−16.22(14)
C(12)-N(2P)-C(2P)-N(1P)	165.61(9)
N(1P)-C(2P)-N(3P)-C(4P)	0.63(16)
N(2P)-C(2P)-N(3P)-C(4P)	−177.33(10)
C(2P)-N(3P)-C(4P)-C(5P)	−1.35(17)
N(3P)-C(4P)-C(5P)-C(6P)	0.80(18)
C(2P)-N(1P)-C(6P)-C(5P)	−1.22(17)
C(4P)-C(5P)-C(6P)-N(1P)	0.56(18)

Symmetry transformations used to generate equivalent atoms.

Table S3.4.6.7. Hydrogen bonds for 4 [Å and °].

D-H...A	d(D-H)	d(H...A)	d(D...A)	<(DHA)
O(12)-H(21)...N(1P)#1	0.803(17)	1.914(18)	2.7106(14)	171.1(17)
N(2P)-H(20)...O(12)#2	0.812(15)	2.223(15)	2.9653(15)	152.1(14)
C(3)-H(3)...O(2)#3	0.95	2.48	3.3641(16)	154.7
C(5P)-H(5P)...O(1)#4	0.95	2.61	3.3975(16)	141.1
C(4P)-H(4P)...O(1)#5	0.95	2.62	3.5053(17)	154.4
C(4P)-H(4P)...N(3P)#5	0.95	2.67	3.3441(17)	128.6

Symmetry transformations used to generate equivalent atoms: #1 x,y+1,z #2 -x+1/2,y-1/2,-z+3/2 #3 -x+1,-y,-z+2 #4 -x,-y,-z+2 #5 -x,-y+1,-z+2 .

3.5. (4-Chloro-3-nitrophenyl)(pyrimidin-2-ylamino)methanol (5)

Synthesis

Acetonitrilic solution (3 mL) of 4-chloro-3-nitrobenzaldehyde (41 mg) was added to an acetonitrilic solution (3 mL) of 2-aminopyrimidine (21 mg). The reaction mixture after complete dissolution was stirred for 2 hours at 50 °C. The title compound was obtained directly from the mother liquor in non-crystalline state. The yellow product was filtered off, washed with a small amount of acetonitrile and diethyl ether then dried in the air to afford (4-chloro-3-nitrophenyl)(pyrimidin-2-ylamino)methanol—(54 mg, 87%), mp 98–99 °C.

3.5.2. Elemental Analysis

	% C	% H	% N
Calculated	47.07	3.23	19.96
Found	46.79	3.34	20.23

3.5.3. Mass Spectrometry

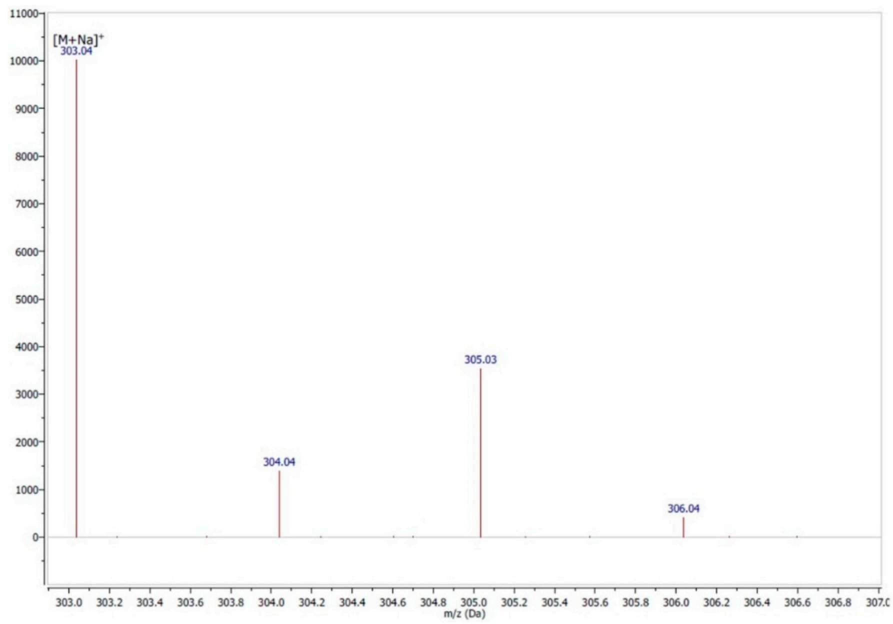

3.5.4. NMR Spectroscopy

^{1}H-NMR

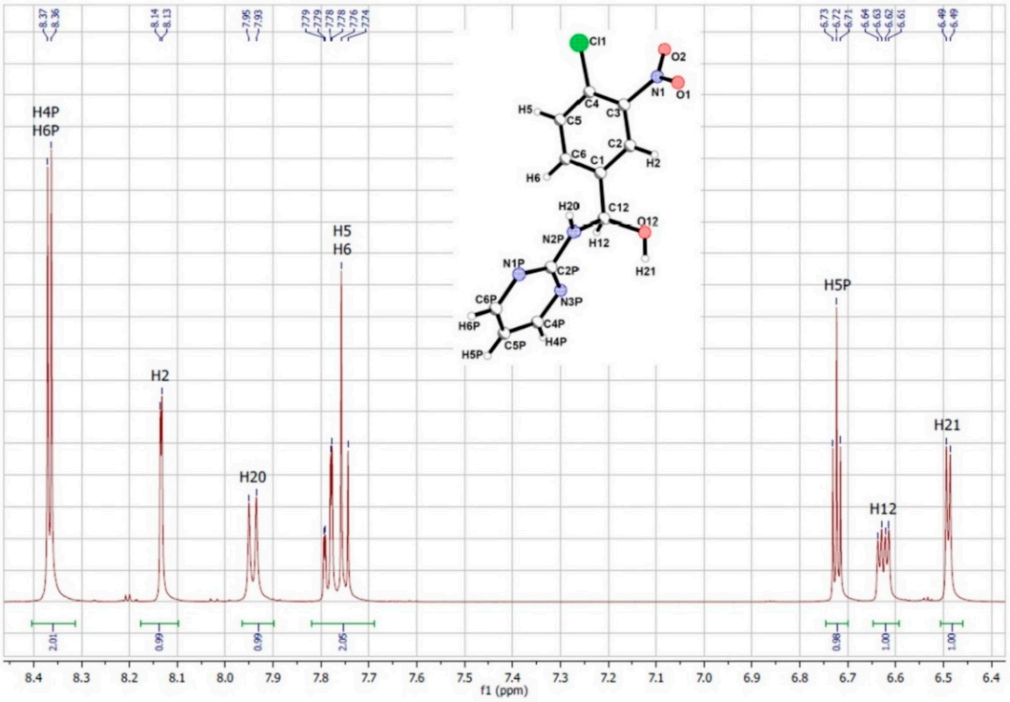

^{1}H-NMR (600 MHz, DMSO, RT) δ: 8.37 (d, $^{3}J_{H4P/H6P,H5P}$ = 4.8 Hz, 2H, H4P,H6P), 8.13 (d, $^{4}J_{H2,H6}$ = 1.8 Hz, 1H,H2), 7.94 (d, $^{3}J_{H20,H12}$ = 9.0 Hz, 1H, H20), 7.77 (m, 2H, H5,H6), 6.72 (t, $^{3}J_{H5P,H4P/H6P}$ = 4.8 Hz, 1H. H5P), 6.62 (dd, $^{3}J_{H12,H20}$ = 9.0 Hz, $^{3}J_{H12,H21}$ = 4.8 Hz, 1H, H12), 6.49 (d, $^{3}J_{H21,H12}$ = 4.7 Hz, 1H, H21).

^{13}C-NMR

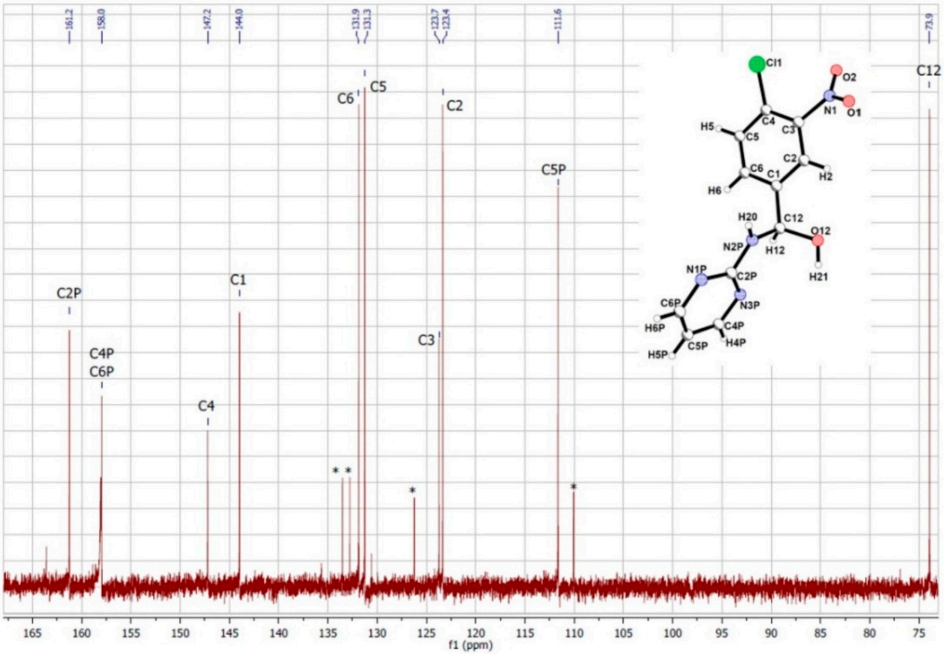

^{13}C-NMR (150.9 MHz, DMSO, RT) δ: 161.2 (C2P), 158.0 (C4P, C6P), 147.2 (C4), 144.0 (C1), 131.9 (C6), 131.3 (C5), 123.7 (C3), 123.4 (C2), 111.6 (C5P), 73.9 (C12).

*—Impurities, results of the fact that in the solution hemiaminals are in a dynamic equilibrium primarily with the initial reagents (aldehyde + amine), in some cases traces of an imine could also be observed.

COSY

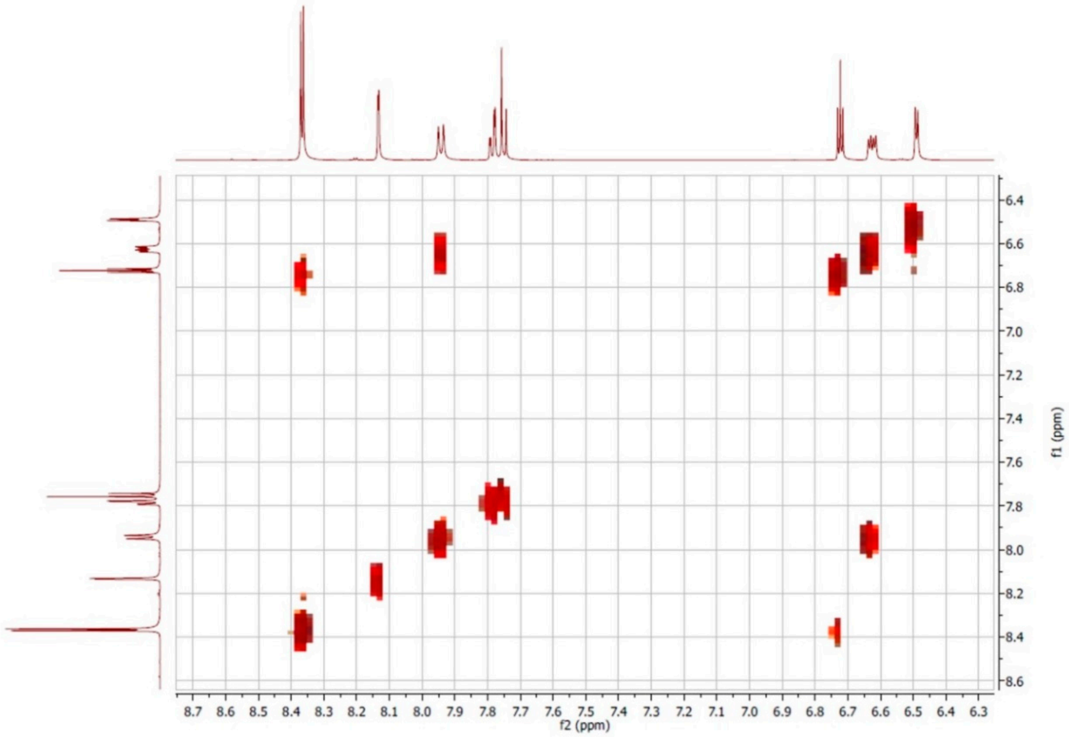

HMQC

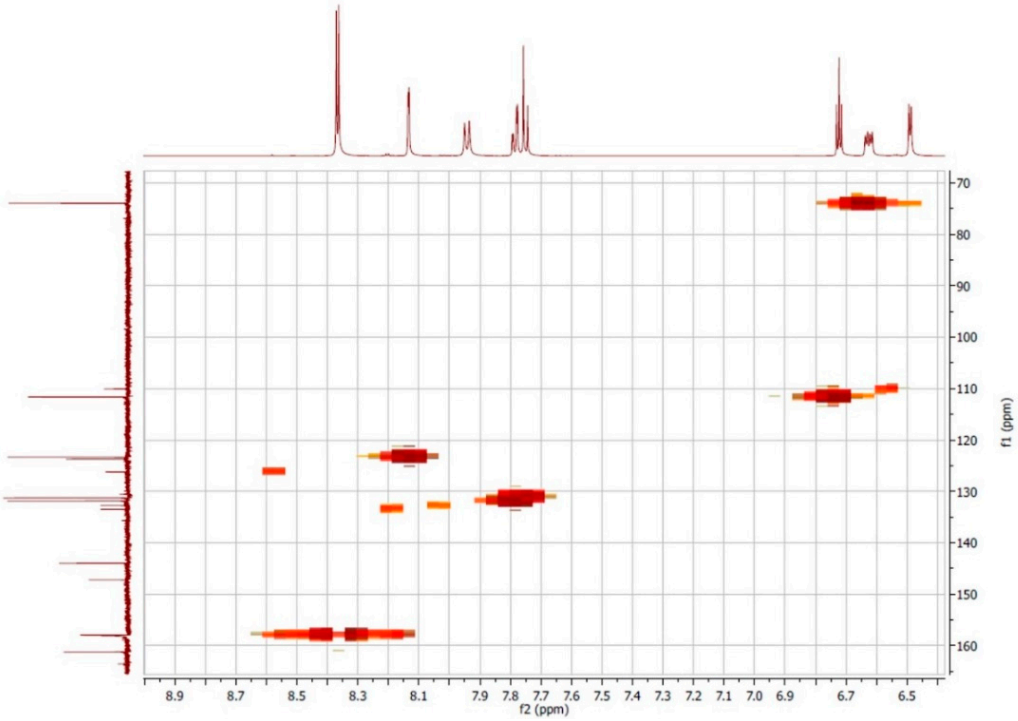

HMBC

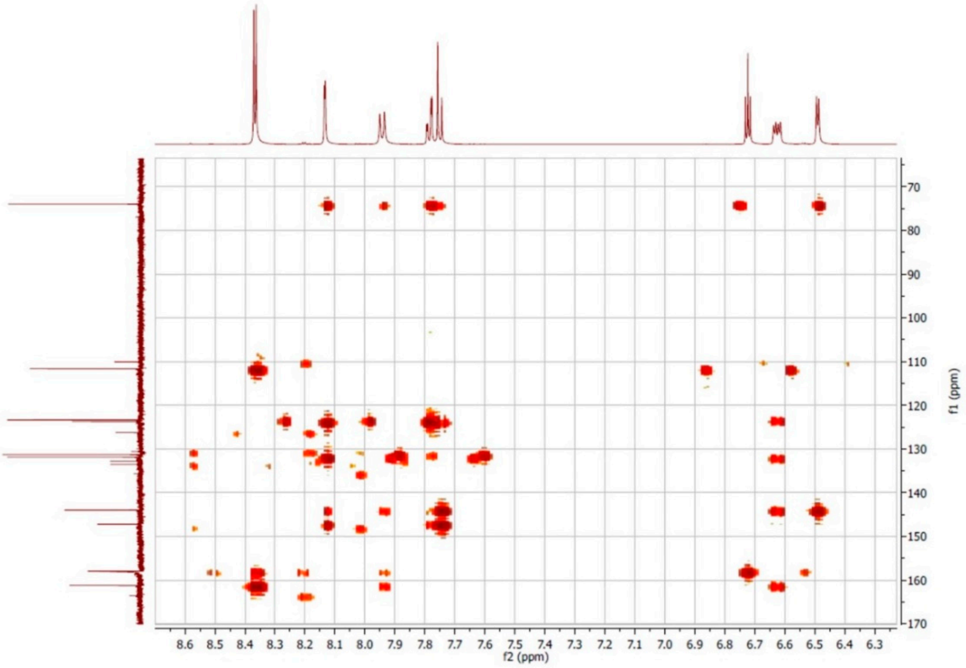

3.5.5. IR Spectroscopy

IR (KBr, cm^{-1}): 3343 s, 3100m, 3026 m, 1611 m, 1589 s, 1574 vs, 1531 vs, 1511 vs, 1457 vs, 1421 m, 1343 s, 1298 m, 1246 s, 1197 w, 1116 m, 1090 w, 1073 m, 1049 s, 1032 s, 998 w, 953 vw, 926 vw, 848 m, 821 w, 807 m, 759 w, 736 w, 669 w, 645 w, 628 w, 585 m, 515 m.

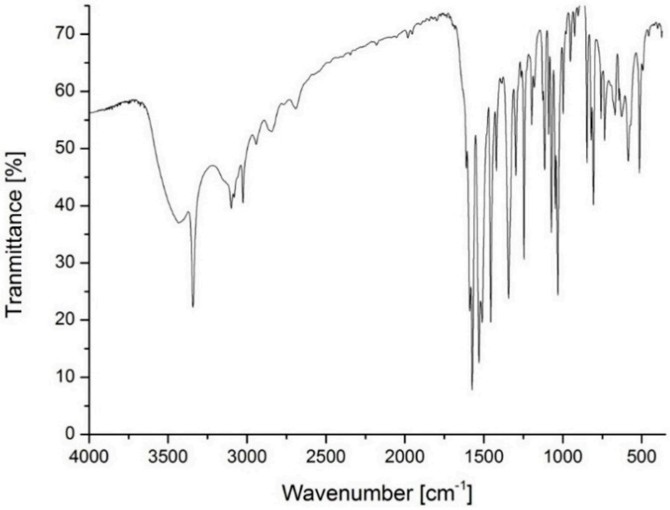

3.6. (5-Fluoro-2-nitrophenyl)(pyrimidin-2-ylamino)methanol (6)

3.6.1. Synthesis

Acetonitrilic solution (3 mL) of 5-fluoro-2-nitrobenzaldehyde (41 mg) was added to an acetonitrilic solution (3 mL) of 2-aminopyrimidine (24 mg). The reaction mixture after complete dissolution was stirred for 2 h at 50 °C. The title compound crystallised directly from the mother liquor. Upon standing 3 days at the room temperature, the solution deposited colourless crystal needles. The crystals were

filtered off, washed with a small amount of acetonitrile and diethyl ether then dried in the air to afford (5-fluoro-2-nitrophenyl)(pyrimidin-2-ylamino)methanol—(60 mg, 93%), mp 116–117 °C.

3.6.2. Elemental Analysis

	% C	% H	% N
Calculated	50.00	3.43	21.20
Found	50.15	3.53	21.34

3.6.3. Mass Spectrometry

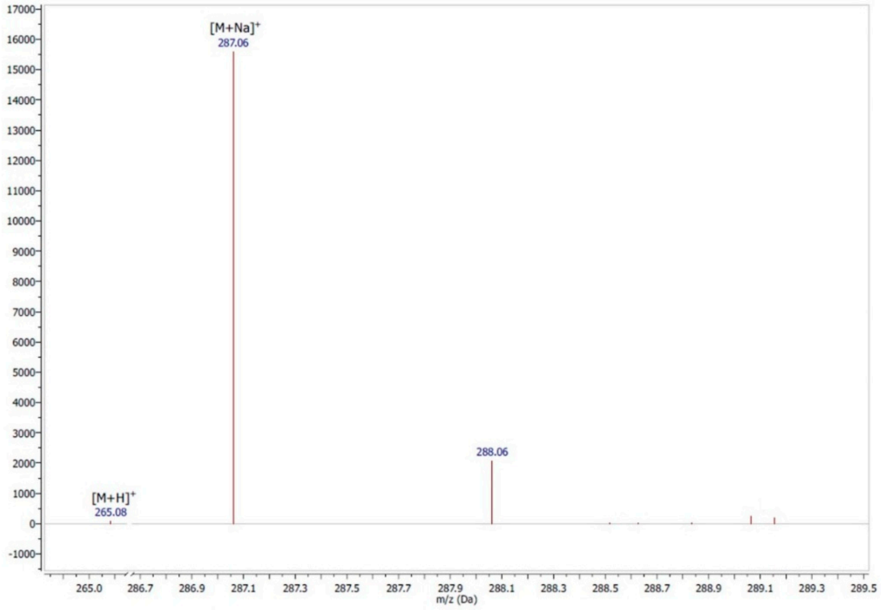

3.6.4. NMR Spectroscopy

^{1}H-NMR

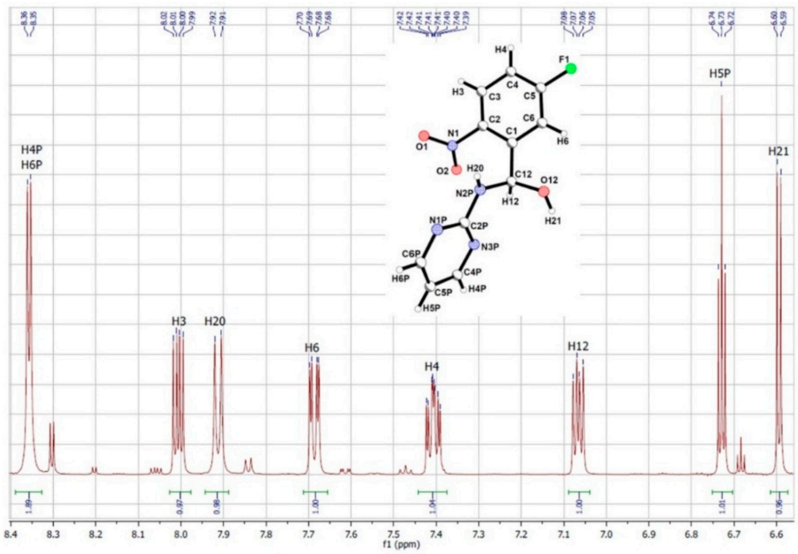

^{1}H-NMR (600 MHz, DMSO, 298 K) δ: 8.36 (d, $^3J_{H4P/H6P,H5P}$ = 4.8, 2H, H4P, H6P), 7.99–8.02 (m, 1H, H3), 7.91 (d, $^3J_{H20,H12}$ = 9.0 Hz, 1H, H20), 7.68–7.70 (m, 1H, H6), 7.39–7.42 (m, 1H, H4), 7.05–7.08 (dd, $^3J_{H12,H20}$ = 9.0 Hz, $^3J_{H12,H21}$ = 5.4 Hz, 1H, H12), 6.73 (t, $^3J_{H5P,H4P/H6P}$ = 4.8 Hz, 1H, H5P), 6.59 (d, $^3J_{H21,H12}$ = 5.4 Hz, 1H, H21).

^{13}C-NMR

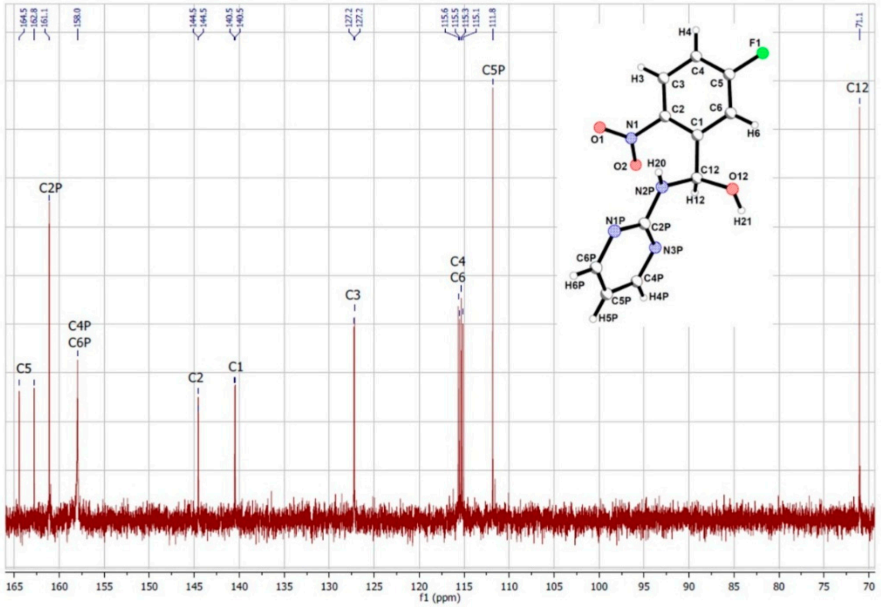

^{13}C-NMR (150.9 MHz, DMSO, 298 K) δ: 163.6 (d, $^1J_{C5,F}$ = 250.3 Hz, C5), 161.1 (C2P), 158.0 (C4P, C6P), 144.5 (d, $^4J_{C2,F}$ = 3.0 Hz, C2), 140.5 (d, $^3J_{C1,F}$ = 7.5 Hz, C1), 127.2 (d, $^3J_{C3,F}$ = 9.0 Hz, C3), 115.1–115.6 (m, C4, C6), 111.8 (C5P), 71.1 (C12).

COSY

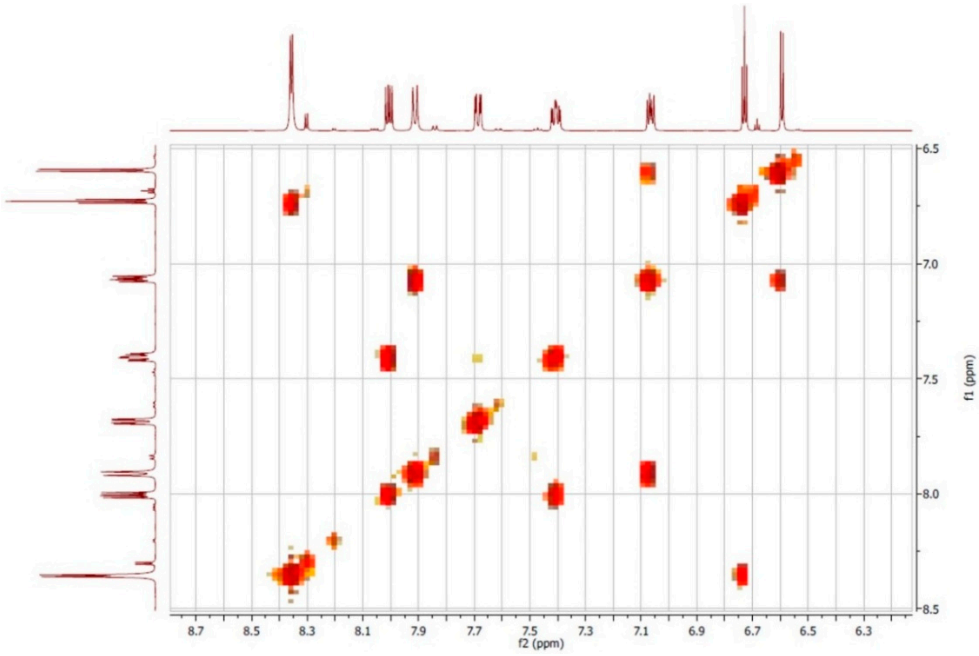

HMQC

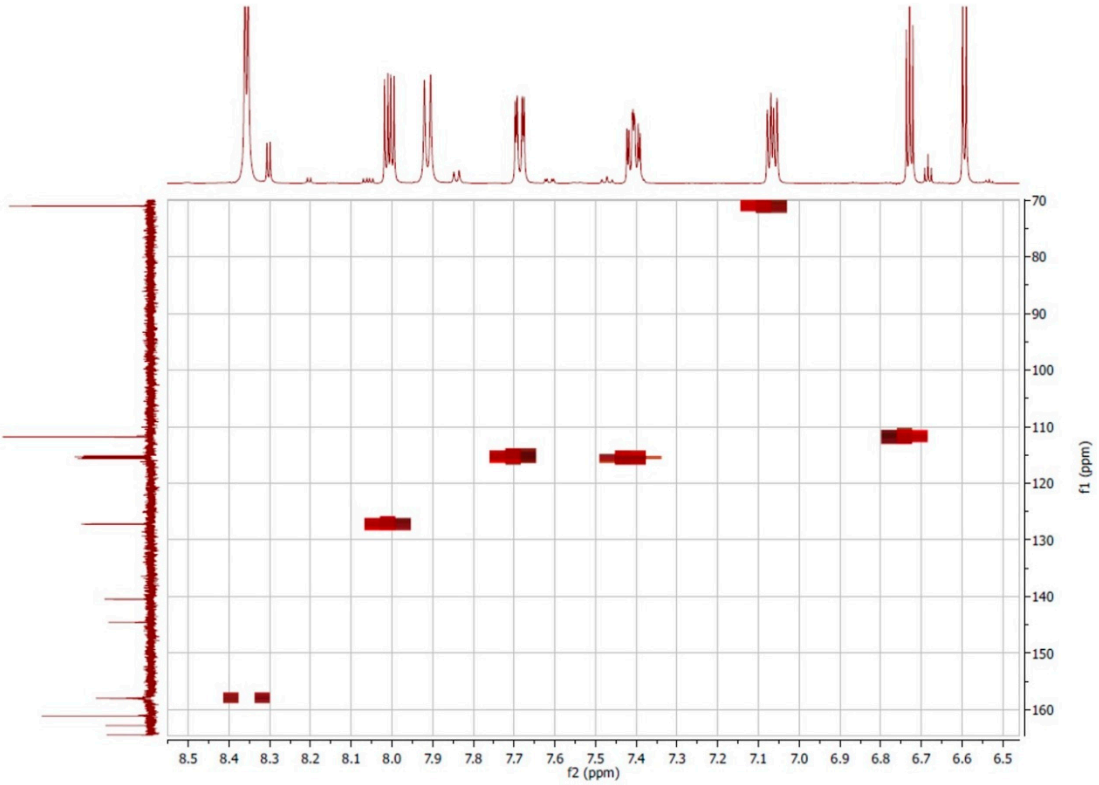

HMBC

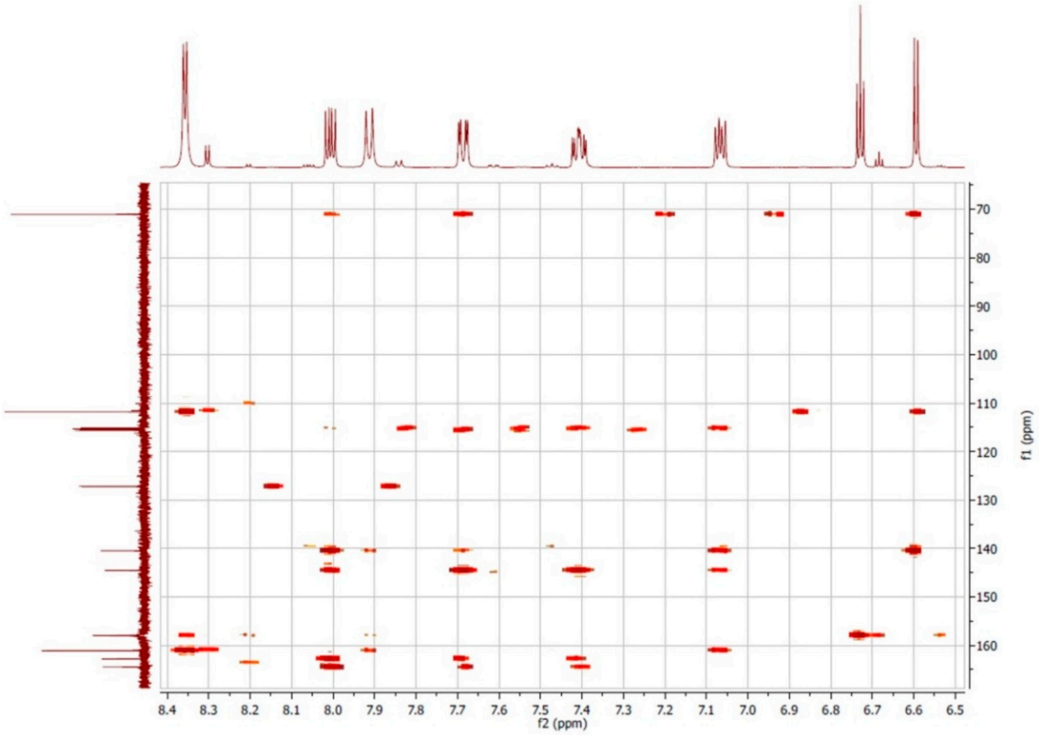

3.6.5. IR Spectroscopy

IR (KBr, cm^{-1}): 3322 m, 3243 w, 3064 m, 2834 w, 2697 w, 2364 vw, 1885 vw, 1695 vw, 1622 m, 1591 vs, 1577 vs, 1521 vs, 1465 s, 1416 s, 1368 vs, 1307 w, 1295 w, 1265 s, 1252 s, 1227 m, 1187 vw,

1151 m, 1131 w, 1115 w, 1086 m, 1071 s, 1033 m, 1001 w, 965 w, 944 vw, 916 vw, 895 w, 847 w, 827 w, 811 m, 781 w, 750 vw, 713 vw, 697 w, 649 w, 630 m, 613 w, 584 w, 572 w, 544 vw, 512 vw, 496 vw, 444 w, 426 vw, 410 vw.

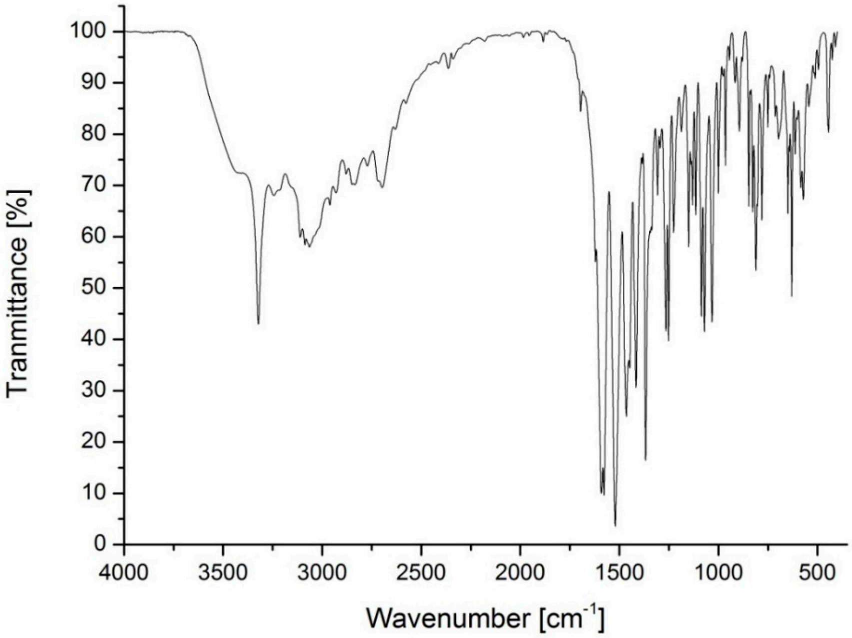

3.6.6. Crystallography

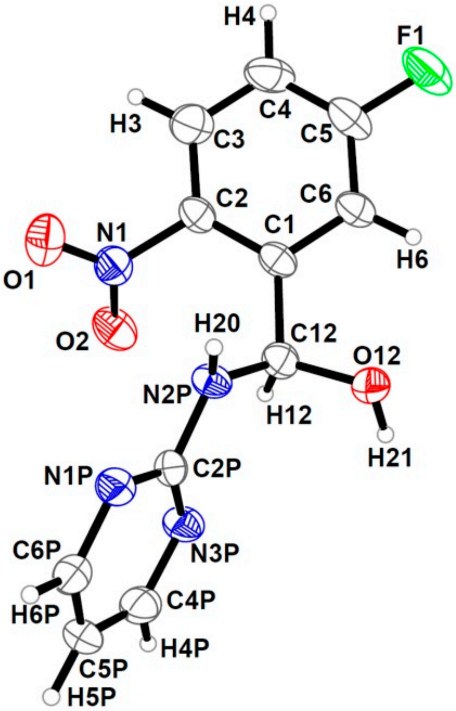

Figure 6. Molecular structure and labelling for (5-fluoro-2-nitrophenyl)(pyrimidin-2-ylamino)methanol (6). Displacement ellipsoids are shown at the 50% probability level.

Table S3.6.6.1. Crystal data and structure refinement for 6.

Identification Code	6	
Empirical formula	C11 H9 F N4 O3	
Formula weight	264.22	
Temperature	100(2) K	
Wavelength	0.71073 Å	
Crystal system	Monoclinic	
Space group	P 1 21/n 1	
Unit cell dimensions	a = 11.182(3) Å	α = 90°.
	b = 5.503(2) Å	β = 90.50(3)°.
	c = 18.942(5) Å	γ = 90°.
Volume	1165.5(6) $Å^3$	
Z	4	
Density (calculated)	1.506 Mg/m^3	
Absorption coefficient	0.123 mm^{-1}	
F(000)	544	
Crystal size	0.58 × 0.29 × 0.19 mm^3	
Theta range for data collection	3.64 to 29.99°.	
Index ranges	$-14 \leq h \leq 15, -5 \leq k \leq 7, -25 \leq l \leq 24$	
Reflections collected	8271	
Independent reflections	2855 [R(int) = 0.0890]	
Completeness to theta = 27.00°	99.6%	
Absorption correction	Semi-empirical from equivalents	
Max. and min. transmission	1.00000 and 0.89691	
Refinement method	Full-matrix least-squares on F^2	
Data/restraints/parameters	2855/0/178	
Goodness-of-fit on F^2	0.998	
Final R indices [I > 2sigma(I)]	R1 = 0.0568, wR2 = 0.0885	
R indices (all data)	R1 = 0.1538, wR2 = 0.0994	
Largest diff. peak and hole	0.268 and −0.227 e.$Å^{-3}$	

Table S3.6.6.2. Atomic coordinates (×10^4) and equivalent isotropic displacement parameters (Å^2 × 10^3) for 6. U(eq) is defined as one third of the trace of the orthogonalized U^{ij} tensor.

	x	y	z	U(eq)
F(1)	6347(1)	−2812(3)	2384(1)	60(1)
C(1)	3825(2)	−84(5)	1470(1)	29(1)
C(2)	4461(2)	1539(5)	1051(1)	31(1)
C(3)	5682(2)	1829(5)	1088(1)	42(1)
C(4)	6331(2)	365(6)	1547(1)	46(1)
C(5)	5719(3)	−1325(5)	1938(1)	42(1)
C(6)	4492(2)	−1573(5)	1912(1)	34(1)
N(1)	3820(2)	2979(4)	508(1)	35(1)
O(1)	4004(2)	5185(4)	487(1)	49(1)
O(2)	3151(2)	1892(3)	99(1)	44(1)
C(12)	2462(2)	−159(5)	1465(1)	32(1)
O(12)	2062(2)	−1579(3)	2046(1)	35(1)
N(2P)	2021(2)	2296(4)	1503(1)	27(1)
N(1P)	533(2)	5183(4)	1432(1)	34(1)
C(2P)	913(2)	2956(5)	1247(1)	29(1)
N(3P)	339(2)	1403(4)	823(1)	35(1)
C(4P)	−717(2)	2170(5)	569(1)	40(1)
C(5P)	−1198(2)	4408(5)	727(2)	42(1)
C(6P)	−532(2)	5848(5)	1173(2)	41(1)

Table S3.6.6.3. Bond lengths [Å] and angles [°] for 6.

F(1)-C(5)	1.367(3)
C(1)-C(6)	1.384(3)
C(1)-C(2)	1.394(3)
C(1)-C(12)	1.524(3)
C(2)-C(3)	1.375(3)
C(2)-N(1)	1.480(3)
C(3)-C(4)	1.386(4)
C(3)-H(3)	0.9500
C(4)-C(5)	1.375(4)
C(4)-H(4)	0.9500
C(5)-C(6)	1.379(4)
C(6)-H(6A)	0.9500
N(1)-O(2)	1.227(2)
N(1)-O(1)	1.232(3)
C(12)-O(12)	1.426(3)
C(12)-N(2P)	1.440(3)
C(12)-H(12)	1.0000

Table S3.6.6.3. *Cont.*

O(12)-H(21)	0.90(3)
N(2P)-C(2P)	1.375(3)
N(2P)-H(20)	0.85(2)
N(1P)-C(6P)	1.335(3)
N(1P)-C(2P)	1.344(3)
C(2P)-N(3P)	1.334(3)
N(3P)-C(4P)	1.339(3)
C(4P)-C(5P)	1.378(4)
C(4P)-H(4P)	0.9500
C(5P)-C(6P)	1.373(3)
C(5P)-H(5P)	0.9500
C(6P)-H(6P)	0.9500
C(6)-C(1)-C(2)	116.7(2)
C(6)-C(1)-C(12)	121.4(2)
C(2)-C(1)-C(12)	121.9(2)
C(3)-C(2)-C(1)	123.8(2)
C(3)-C(2)-N(1)	116.6(2)
C(1)-C(2)-N(1)	119.5(2)
C(2)-C(3)-C(4)	118.5(3)
C(2)-C(3)-H(3)	120.7
C(4)-C(3)-H(3)	120.7
C(5)-C(4)-C(3)	118.1(3)
C(5)-C(4)-H(4)	121.0
C(3)-C(4)-H(4)	121.0
F(1)-C(5)-C(4)	118.9(3)
F(1)-C(5)-C(6)	117.9(3)
C(4)-C(5)-C(6)	123.2(3)
C(5)-C(6)-C(1)	119.5(3)
C(5)-C(6)-H(6A)	120.2
C(1)-C(6)-H(6A)	120.2
O(2)-N(1)-O(1)	124.1(2)
O(2)-N(1)-C(2)	117.8(2)
O(1)-N(1)-C(2)	118.0(2)
O(12)-C(12)-N(2P)	111.4(2)
O(12)-C(12)-C(1)	109.3(2)
N(2P)-C(12)-C(1)	108.5(2)
O(12)-C(12)-H(12)	109.2
N(2P)-C(12)-H(12)	109.2
C(1)-C(12)-H(12)	109.2
C(12)-O(12)-H(21)	106.9(17)
C(2P)-N(2P)-C(12)	122.6(2)
C(2P)-N(2P)-H(20)	116.7(16)
C(12)-N(2P)-H(20)	115.3(16)
C(6P)-N(1P)-C(2P)	116.0(2)

Table S3.6.6.3. *Cont.*

N(3P)-C(2P)-N(1P)	126.1(2)
N(3P)-C(2P)-N(2P)	118.1(2)
N(1P)-C(2P)-N(2P)	115.8(2)
C(2P)-N(3P)-C(4P)	115.7(2)
N(3P)-C(4P)-C(5P)	123.2(3)
N(3P)-C(4P)-H(4P)	118.4
C(5P)-C(4P)-H(4P)	118.4
C(6P)-C(5P)-C(4P)	116.1(3)
C(6P)-C(5P)-H(5P)	122.0
C(4P)-C(5P)-H(5P)	122.0
N(1P)-C(6P)-C(5P)	123.0(3)
N(1P)-C(6P)-H(6P)	118.5
C(5P)-C(6P)-H(6P)	118.5

Symmetry transformations used to generate equivalent atoms.

Table S3.6.6.4. Anisotropic displacement parameters (Å^2 × 10^3)for 6. The anisotropic displacement factor exponent takes the form: $-2\pi^2$ [$h^2a^{*2}U^{11}$ + ... + 2 h k a* b* U^{12}].

	U^{11}	U^{22}	U^{33}	U^{23}	U^{13}	U^{12}
F(1)	43(1)	89(1)	48(1)	14(1)	−8(1)	23(1)
C(1)	30(2)	32(2)	24(1)	−5(1)	−5(1)	5(1)
C(2)	31(2)	35(2)	25(1)	0(1)	−3(1)	4(1)
C(3)	36(2)	57(2)	33(2)	2(2)	4(1)	0(2)
C(4)	25(2)	74(2)	38(2)	−1(2)	1(1)	2(2)
C(5)	37(2)	57(2)	32(2)	1(2)	−6(1)	17(2)
C(6)	31(2)	42(2)	28(2)	−1(1)	−6(1)	3(1)
N(1)	34(1)	42(2)	31(1)	4(1)	6(1)	4(1)
O(1)	58(1)	37(1)	51(1)	8(1)	8(1)	2(1)
O(2)	49(1)	55(1)	29(1)	−2(1)	−9(1)	5(1)
C(12)	33(2)	34(2)	29(2)	1(1)	−4(1)	−2(1)
O(12)	31(1)	39(1)	35(1)	5(1)	−5(1)	−7(1)
N(2P)	27(1)	28(1)	27(1)	−6(1)	−6(1)	0(1)
N(1P)	28(1)	30(1)	44(1)	3(1)	−2(1)	2(1)
C(2P)	28(2)	35(2)	25(2)	8(1)	−1(1)	−1(1)
N(3P)	31(1)	42(2)	31(1)	−1(1)	−9(1)	−4(1)
C(4P)	34(2)	52(2)	34(2)	7(2)	−7(1)	−9(2)
C(5P)	26(2)	50(2)	49(2)	18(2)	−9(2)	0(2)
C(6P)	31(2)	35(2)	58(2)	13(2)	5(2)	−1(1)

Table S3.6.6.5. Hydrogen coordinates (×10[4]) and isotropic displacement parameters (Å[2] × 10[3]) for 6.

	x	y	z	U(eq)
H(3)	6071	3008	804	50
H(4)	7174	525	1590	55
H(6A)	4107	−2758	2196	41
H(12)	2177	−921	1015	38
H(21)	1510(20)	−2630(50)	1875(14)	52
H(20)	2310(20)	3140(40)	1838(12)	33
H(4P)	−1155	1120	264	48
H(5P)	−1946	4923	539	50
H(6P)	−843	7389	1303	49

Table S3.6.6. Torsion angles [°] for 6.

C(6)-C(1)-C(2)-C(3)	−4.2(4)
C(12)-C(1)-C(2)-C(3)	173.6(2)
C(6)-C(1)-C(2)-N(1)	172.9(2)
C(12)-C(1)-C(2)-N(1)	−9.3(3)
C(1)-C(2)-C(3)-C(4)	2.6(4)
N(1)-C(2)-C(3)-C(4)	−174.5(2)
C(2)-C(3)-C(4)-C(5)	0.7(4)
C(3)-C(4)-C(5)-F(1)	178.9(2)
C(3)-C(4)-C(5)-C(6)	−2.3(4)
F(1)-C(5)-C(6)-C(1)	179.5(2)
C(4)-C(5)-C(6)-C(1)	0.6(4)
C(2)-C(1)-C(6)-C(5)	2.5(4)
C(12)-C(1)-C(6)-C(5)	−175.3(2)
C(3)-C(2)-N(1)-O(2)	127.2(3)
C(1)-C(2)-N(1)-O(2)	−50.1(3)
C(3)-C(2)-N(1)-O(1)	−51.6(3)
C(1)-C(2)-N(1)-O(1)	131.1(2)
C(6)-C(1)-C(12)-O(12)	10.4(3)
C(2)-C(1)-C(12)-O(12)	−167.3(2)
C(6)-C(1)-C(12)-N(2P)	132.1(2)
C(2)-C(1)-C(12)-N(2P)	−45.6(3)
O(12)-C(12)-N(2P)-C(2P)	−85.0(3)
C(1)-C(12)-N(2P)-C(2P)	154.6(2)
C(6P)-N(1P)-C(2P)-N(3P)	1.5(4)
C(6P)-N(1P)-C(2P)-N(2P)	178.7(2)
C(12)-N(2P)-C(2P)-N(3P)	−14.2(3)
C(12)-N(2P)-C(2P)-N(1P)	168.3(2)
N(1P)-C(2P)-N(3P)-C(4P)	−0.6(4)
N(2P)-C(2P)-N(3P)-C(4P)	−177.7(2)
C(2P)-N(3P)-C(4P)-C(5P)	0.0(4)
N(3P)-C(4P)-C(5P)-C(6P)	−0.4(4)
C(2P)-N(1P)-C(6P)-C(5P)	−1.9(4)
C(4P)-C(5P)-C(6P)-N(1P)	1.4(4)

Symmetry transformations used to generate equivalent atoms.

Table S3.6.6.7. Hydrogen bonds for 6 [Å and °].

D-H...A	d(D-H)	d(H...A)	d(D...A)	<(DHA)
O(12)-H(21)...N(1P)#1	0.90(3)	1.83(3)	2.724(3)	172(3)
N(2P)-H(20)...O(12)#2	0.85(2)	2.23(2)	2.989(3)	149(2)
C(5P)-H(5P)...O(2)#3	0.95	2.51	3.362(3)	148.8

Symmetry transformations used to generate equivalent atoms: #1 x,y-1,z #2 -x+1/2,y+1/2,-z+1/2 #3 -x,-y+1,-z .

3.7. (2-Fluoro-4-nitrophenyl)(pyrimidin-2-ylamino)methanol (7)

3.7.1. Synthesis

Acetonitrilic solution (3 mL) of 2-fluoro-4-nitrobenzaldehyde (40 mg) was added to an acetonitrilic solution (3 mL) of 2-aminopyrimidine (22 mg). The reaction mixture after complete dissolution was stirred for 2 h at 50 °C. The title compound crystallised directly from the mother liquor. Upon standing 3 days at the room temperature, the solution deposited colourless crystal blocks. The crystals were filtered off, washed with a small amount of acetonitrile and diethyl ether then dried in the air to afford (2-fluoro-4-nitrophenyl)(pyrimidin-2-ylamino)methanol—(57 mg, 92%), mp 105 °C.

3.7.2. Elemental Analysis

	% C	% H	% N
Calculated	50.00	3.43	21.20
Found	49.97	3.47	21.35

3.7.3. Mass Spectrometry

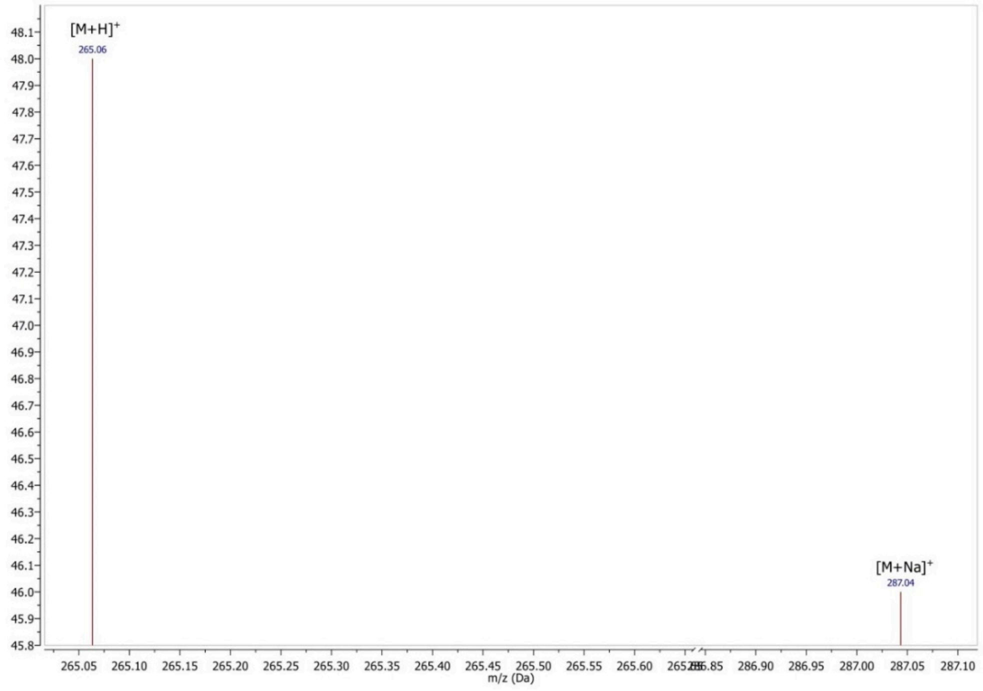

3.7.4. NMR Spectroscopy

^{1}H-NMR

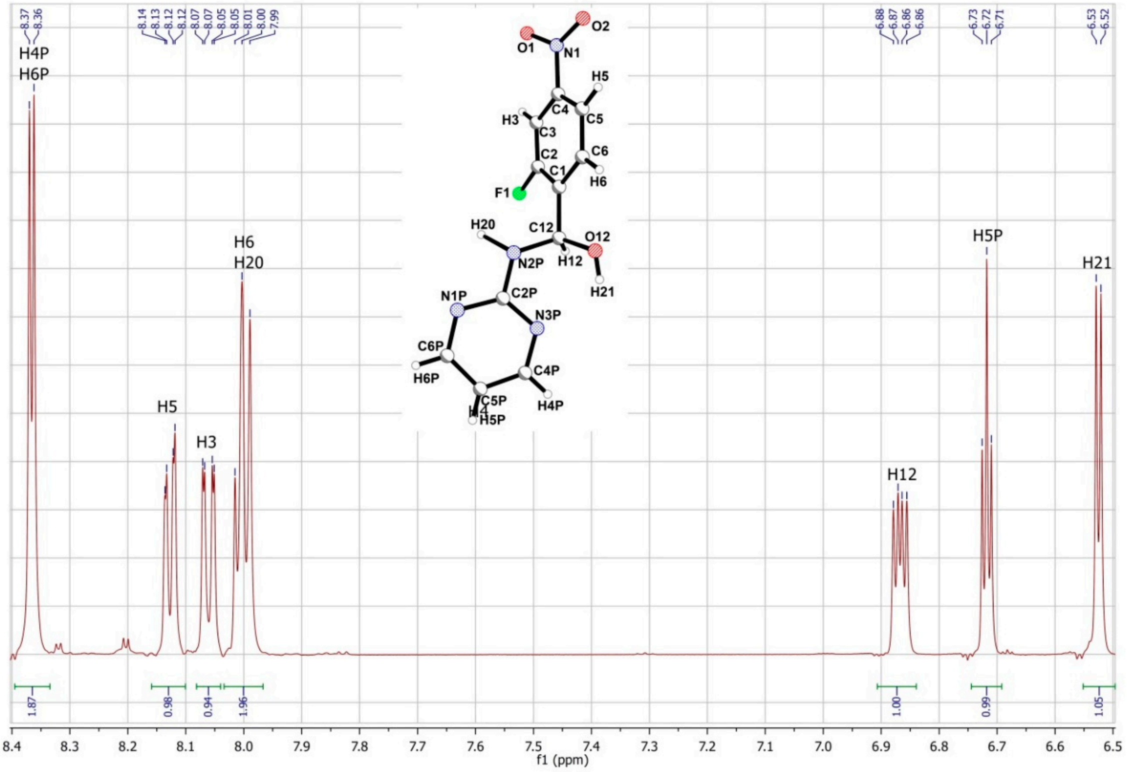

^{1}H-NMR (600 MHz, DMSO, RT) δ: 8.36 (d, $^{3}J_{H4P/H6P,H5P}$ = 4.8, 2H, H4P, H6P), 8.12–8.14 (m, 1H, H5), 8.05–8.07 (m, 1H, H3), 7.99–8.01 (m, 2H, H6, H20), 6.86–6.88 (m, 1H, H12), 6.72 (t, $^{3}J_{H5P,H4P/H6P}$ = 4.8 Hz, 1H, H5P), 6.52 (d, $^{3}J_{H21,H12}$ = 4.8 Hz, 1H, H21).

^{13}C-NMR

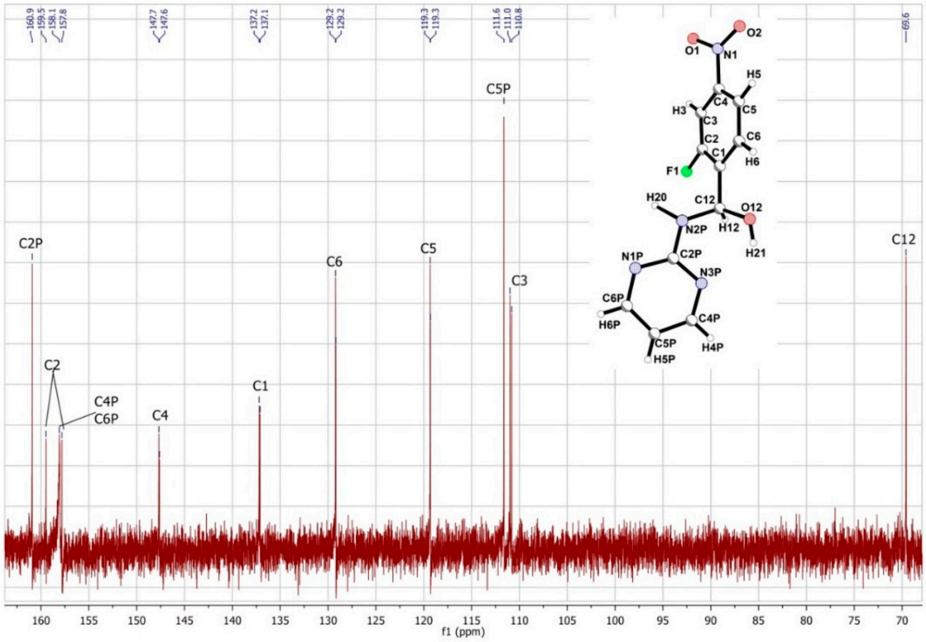

^{13}C-NMR (150.9 MHz, DMSO, RT) δ: 160.9 (C2P), 158.6 (d, $^{1}J_{C2,F}$ = 250.5 Hz, C2), 158.1 (C4P, C6P), 147.7 (d, $^{3}J_{C4,F}$ = 9.0 Hz, C4), 137.2 (d, $^{2}J_{C1,F}$ = 13.6 Hz, C1), 129.2 (d, $^{3}J_{C6,F}$ = 4.5 Hz, C6), 119.3 (d, $^{4}J_{C5,F}$ = 3.0 Hz, C5), 111.6 (C5P), 110.9 (d, $^{2}J_{C3,F}$ = 27.2 Hz, C3), 69.6 (C12).

COSY

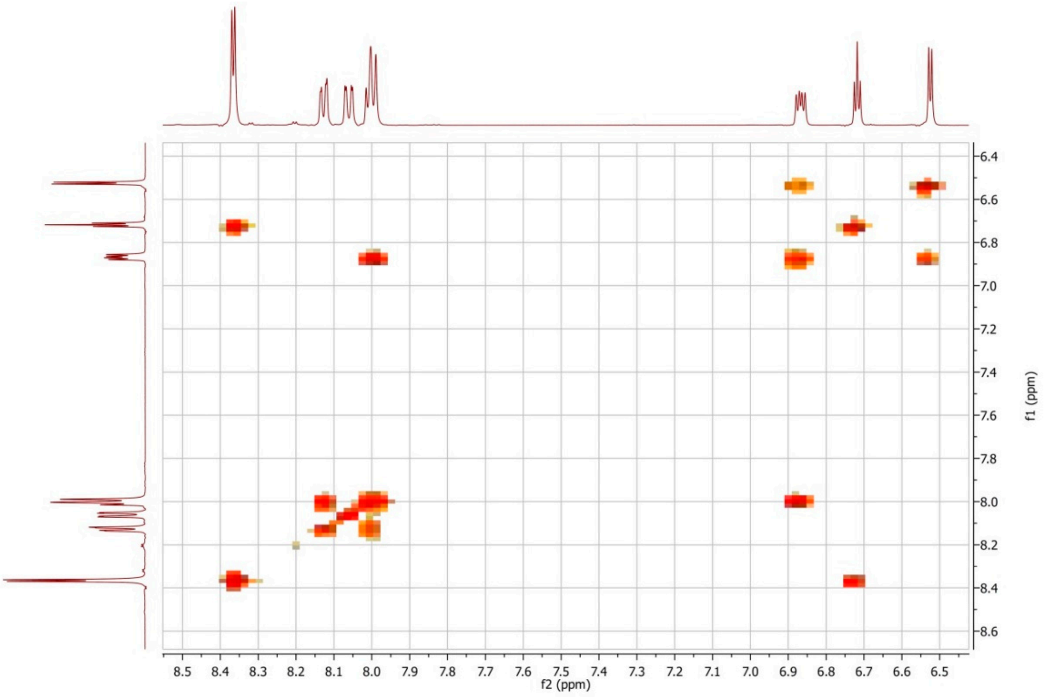

HMQC

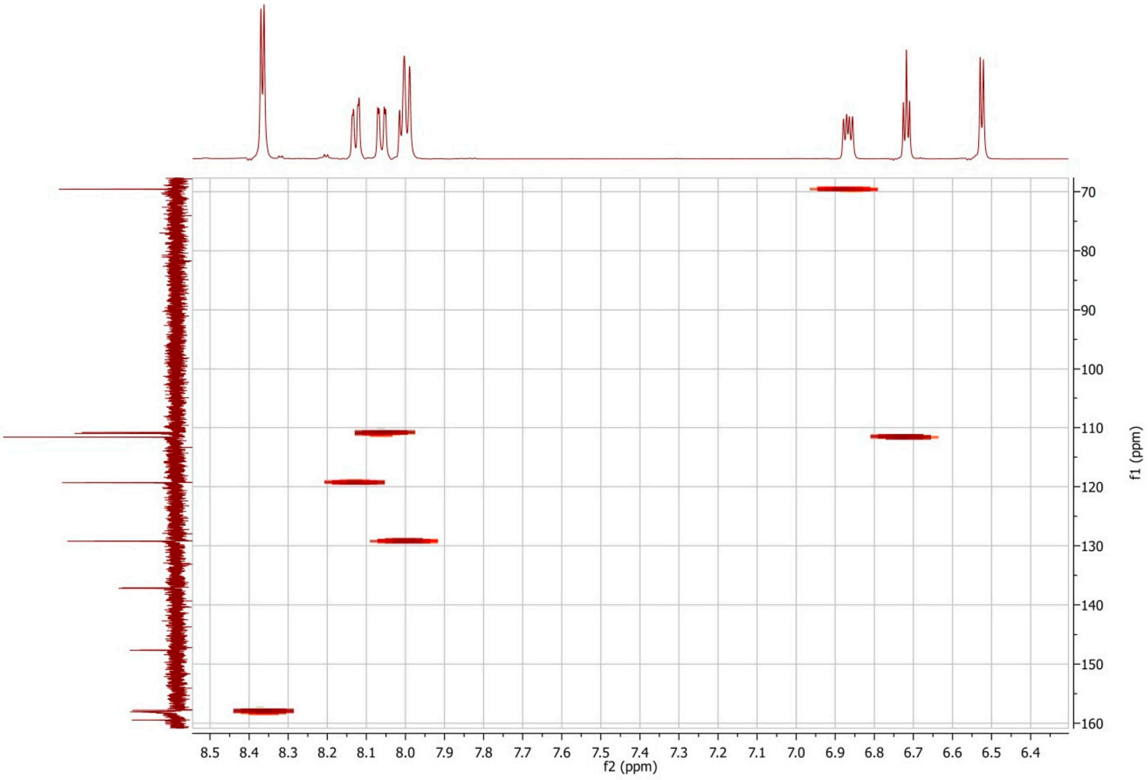

HMBC

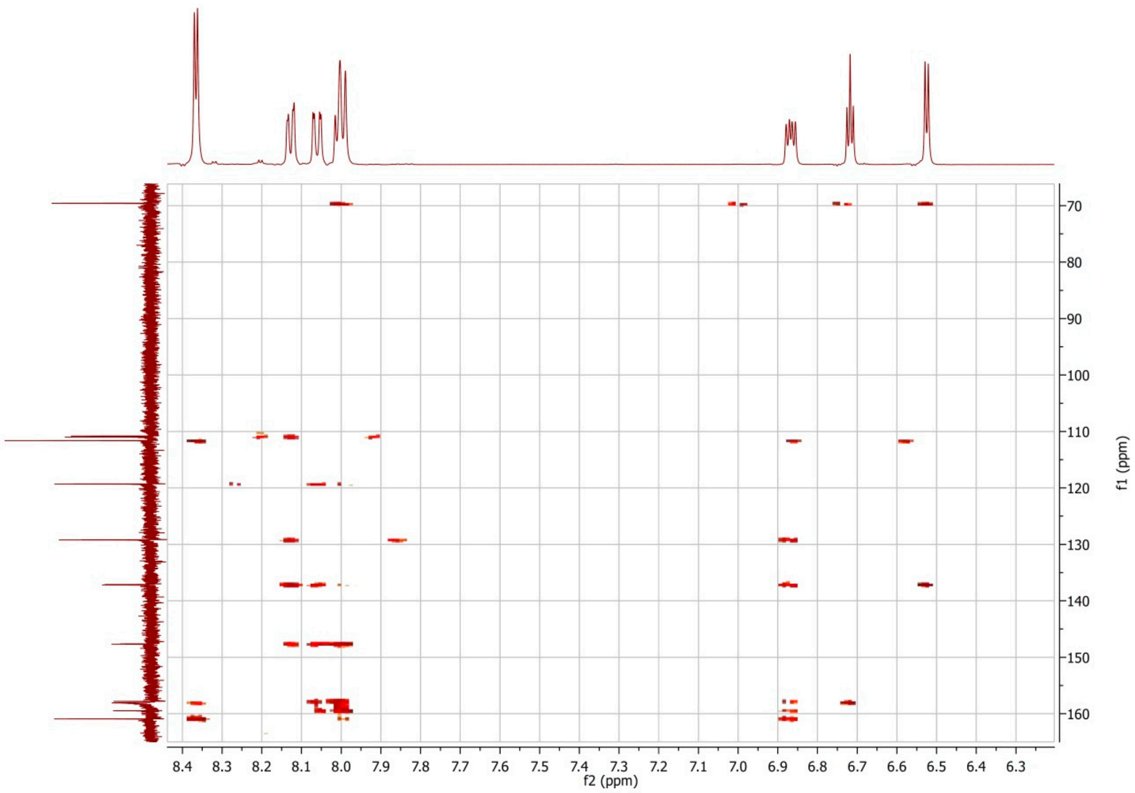

IR Spectroscopy

IR (KBr, cm^{-1}): 3387 m, 3265 w, 3116 w, 3069 w, 3045 w, 2909 vw, 2360 vw, 1947 vw, 1588 vs, 1573 vs, 1522 vs, 1490 m, 1452 vs, 1432 m, 1416 s, 1383 w, 1356 vs, 1335 s, 1250 m, 1231 m, 1196 w, 1178 m, 1113 m, 1090 w, 1070 m, 1042 s, 992 vw, 976 vw, 943 w, 884 m, 846 vw, 819 m, 809 s, 804 s, 743 s, 722 vw, 677 vw, 633 w, 585 vw, 538 vw, 511 w, 493 vw, 464 vw, 408 vw.

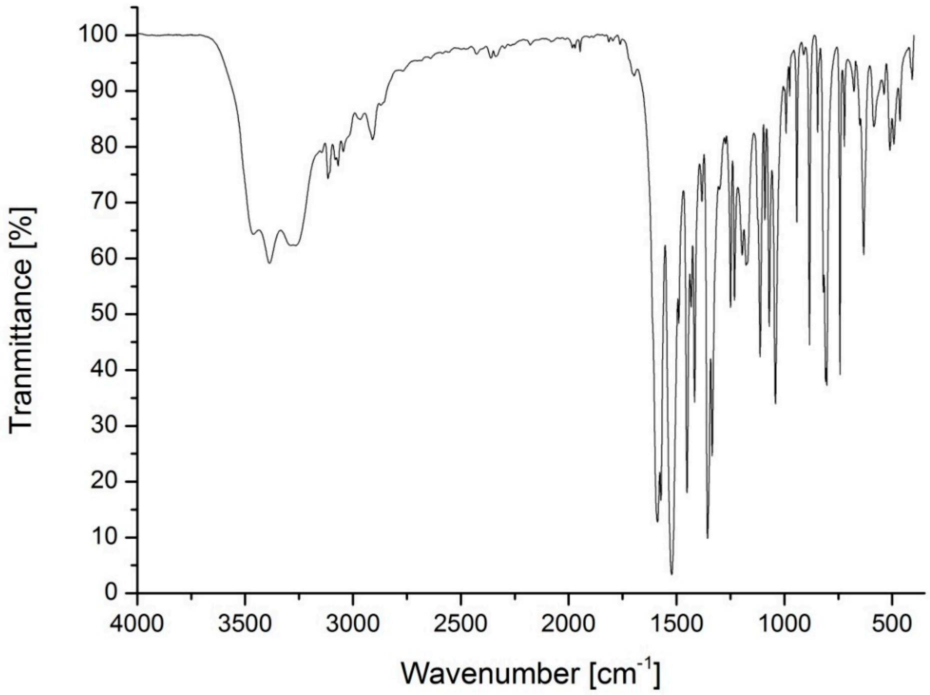

3.8. (4-Fluoro-2-nitrophenyl)(pyrimidin-2-ylamino)methanol (8)

3.8.1. Synthesis

Acetonitrilic solution (3 mL) of 4-fluoro-2-nitrobenzaldehyde (45 mg) was added to an acetonitrilic solution (3 mL) of 2-aminopyrimidine (25 mg). The reaction mixture after complete dissolution was stirred for 2 hours at 50 °C. The title compound was deposited directly from the mother liquor in a non-crystalline state. Obtained product was filtered off, washed with a small amount of acetonitrile and diethyl ether then dried in the air to afford (4-fluoro-2-nitrophenyl)(pyrimidin-2-ylamino)methanol—(62 mg, 89%), mp 90 °C.

3.8.2. Elemental Analysis

	% C	% H	% N
Calculated	50.00	3.43	21.20
Found	49.81	3.20	21.30

3.8.3. Mass Spectrometry

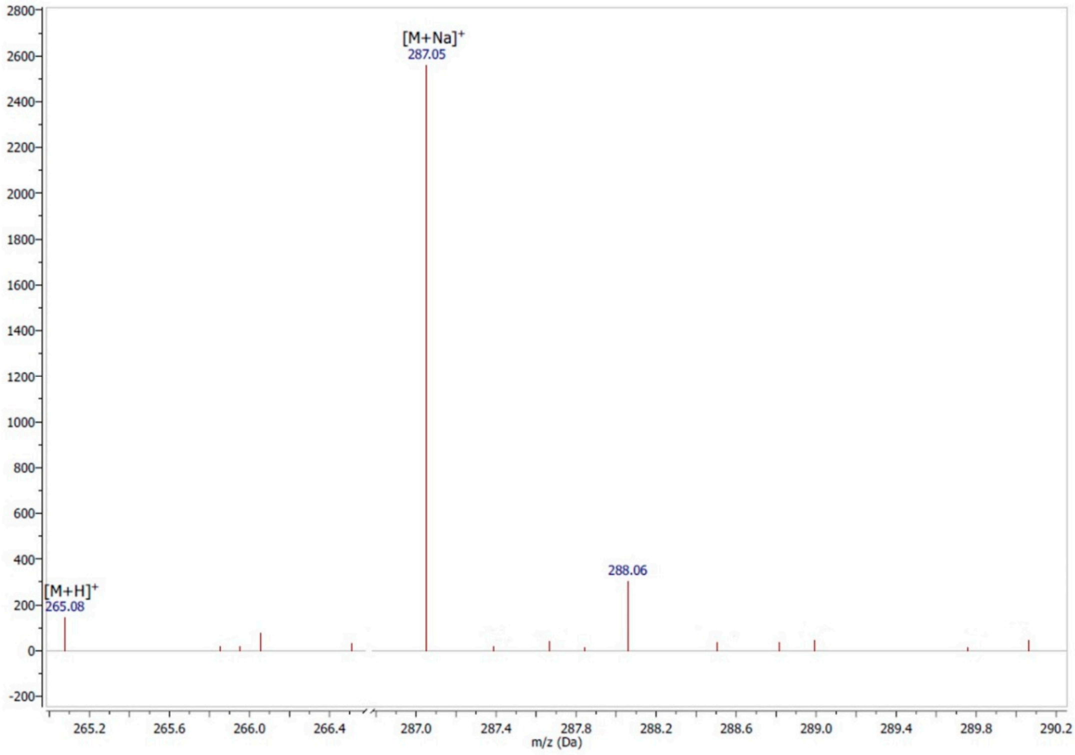

3.8.4. NMR Spectroscopy

^{1}H-NMR

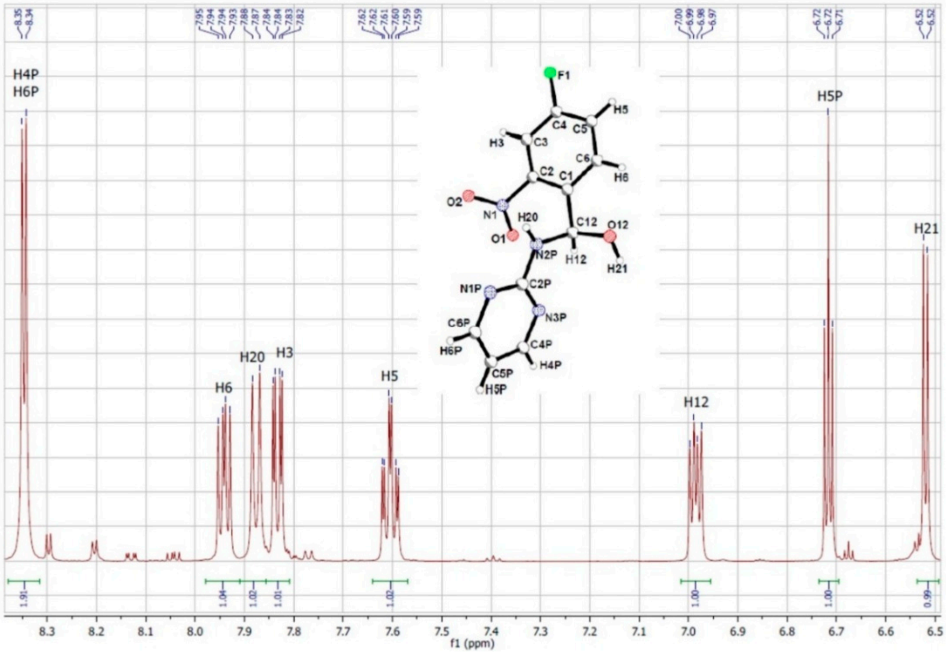

^{1}H-NMR (600 MHz, DMSO, 298 K) δ: 8.35 (d, $^{3}J_{H4P/H6P,H5P}$ = 4.8, 2H, H4P, H6P), 7.93–7.95 (m, 1H, H6), 7.88 (d, $^{3}J_{H20,H12}$ = 9.0 Hz, 1H, H20), 7.82–7.84 (m, 1H, H3), 7.59–7.62 (m, 1H, H5), 6.97–7.00 (dd, $^{3}J_{H12,H20}$ = 9.0 Hz, $^{3}J_{H12,H21}$ = 5.4 Hz, 1H, H12), 6.72 (t, $^{3}J_{H5P,H4P/H6P}$ = 4.8 Hz, 1H, H5P), 6.52 (d, $^{3}J_{H21,H12}$ = 5.4 Hz, 1H, H21).

^{13}C-NMR

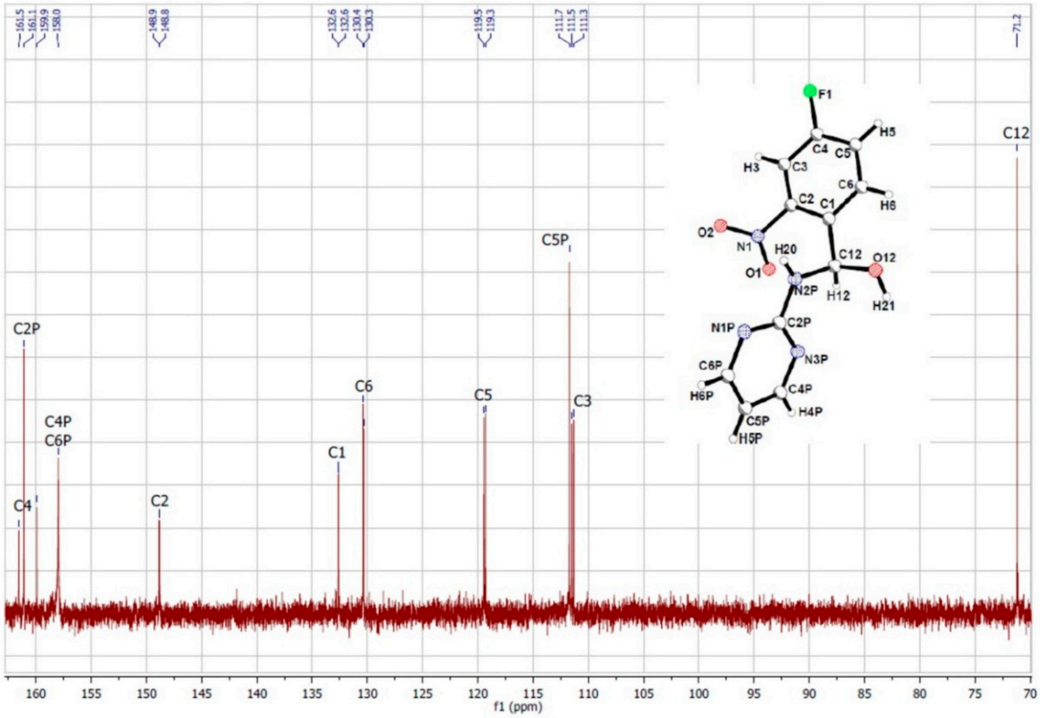

^{13}C-NMR (150.9 MHz, DMSO, 298 K) δ: 160.7 (d, $^{1}J_{C4,F}$ = 247.3 Hz, C4), 161.1 (C2P), 158.0 (C4P, C6P), 148.8 (d, $^{3}J_{C2,F}$ = 9.0 Hz, C2), 132.6 (d, $^{4}J_{C1,F}$ = 4.5 Hz, C1), 130.4 (d, $^{3}J_{C6,F}$ = 7.5 Hz, C6), 119.4 (d, $^{2}J_{C5,F}$ = 21.1 Hz, C5), 111.7 (C5P), 111.4 (d, $^{2}J_{C3,F}$ = 27.1 Hz, C3), 71.2 (C12).

COSY

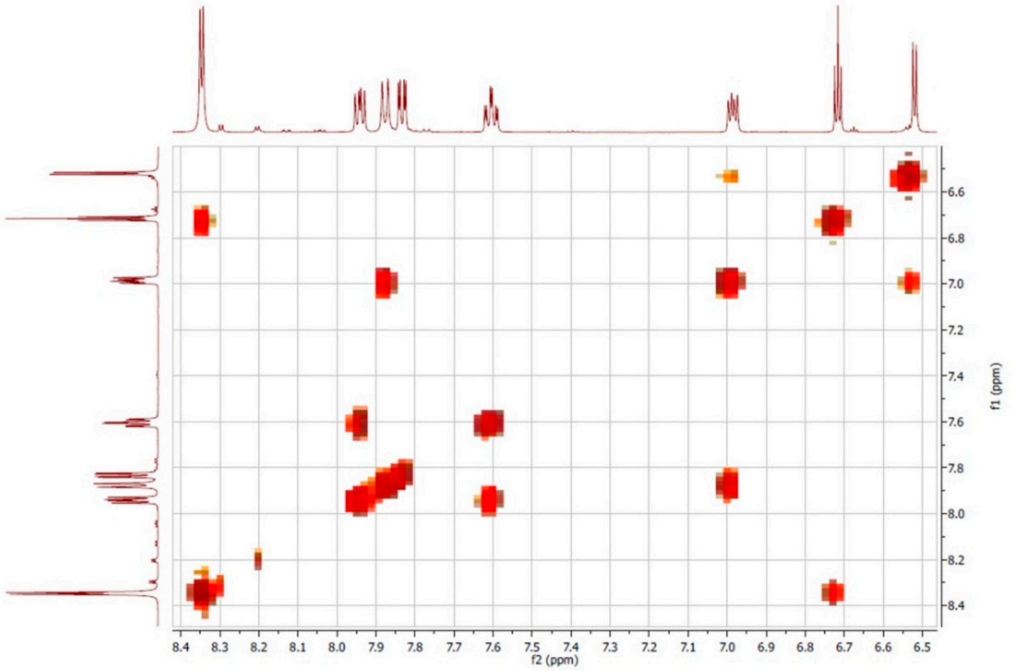

HMQC

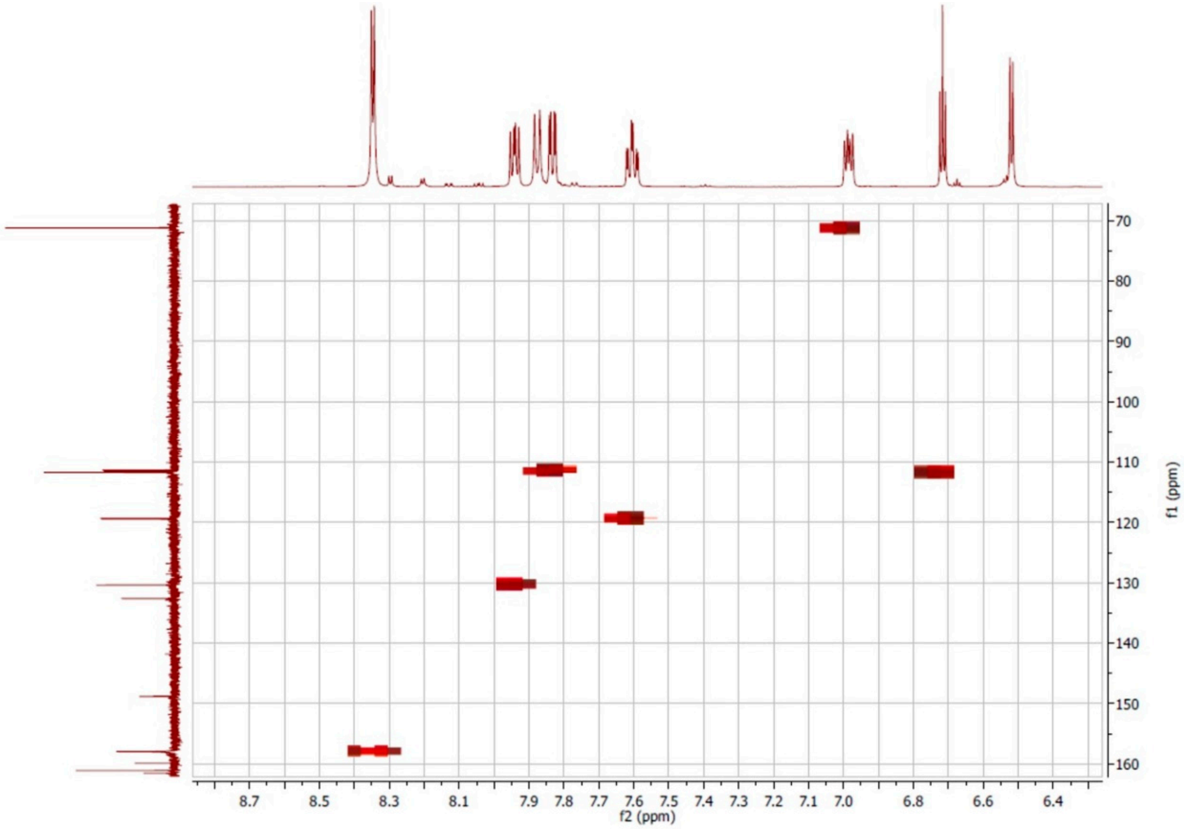

HMBC

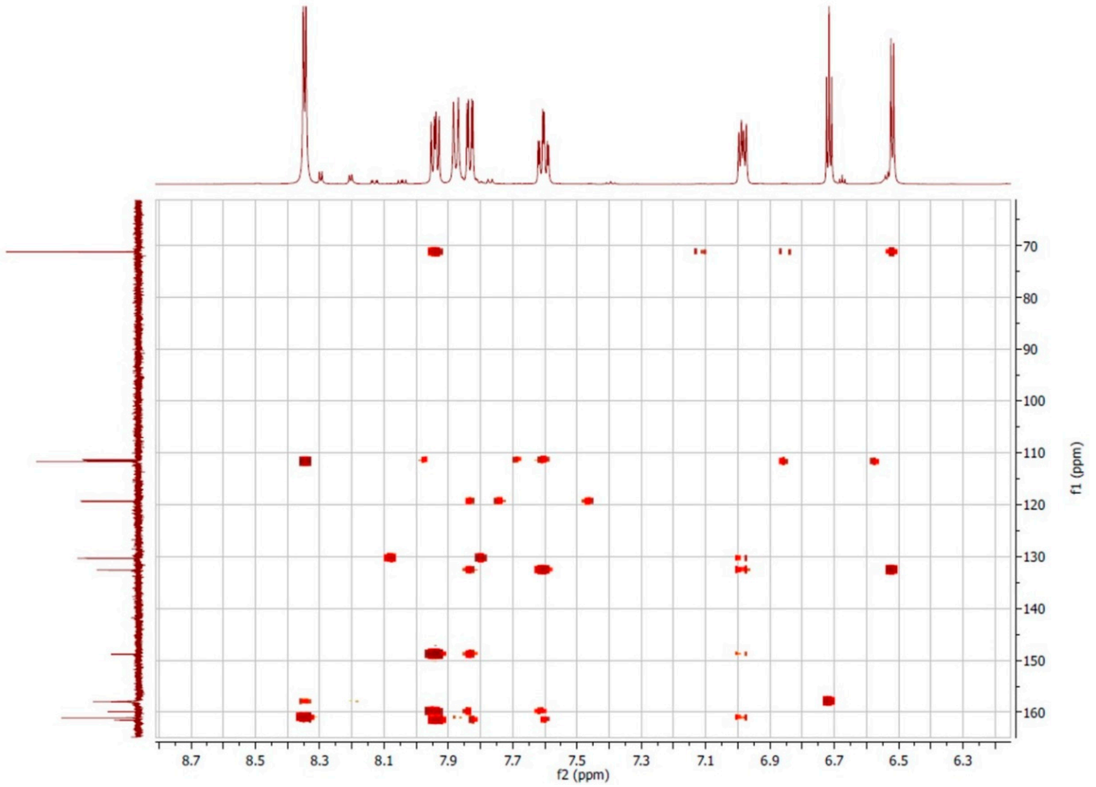

3.8.5. IR Spectroscopy

IR (KBr, cm^{-1}): 3314 s, 3087 s, 2849 m, 3693 w, 2361 vw, 1971 vw, 1936 vw, 1615 s, 1578 vs, 1529 vs, 1491 vs, 1465 vs, 1425 vs, 1369 vs, 1298 m, 1277 m, 1260 s, 1253 s, 1228 vs, 1185 w, 1130 w, 1114 s, 1090 s, 1066 vs, 1037 vs, 998 m, 970 w, 942 s, 913 vw, 877 m, 845 vs, 823 s, 811 vs, 800 s, 746 m, 675 m, 648 w, 628 w, 584 vs, 551 m, 530 m, 454 vw, 428 w.

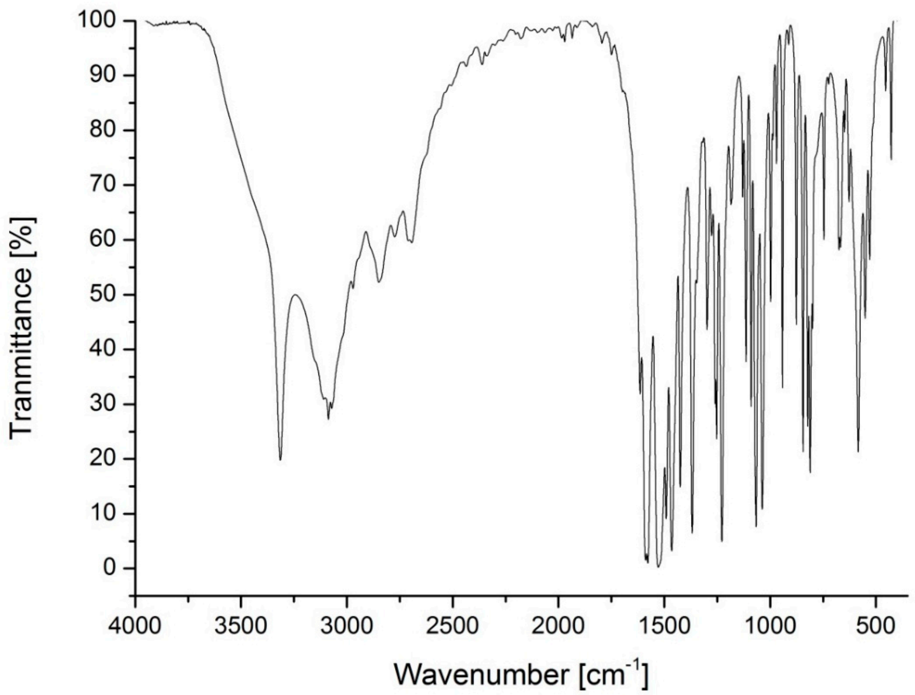

3.9. (4-Fluoro-3-nitrophenyl)(pyrimidin-2-ylamino)methanol (9)

3.9.1. Synthesis

Acetonitrilic solution (3 mL) of 4-fluoro-3-nitrobenzaldehyde (39 mg) was added to an acetonitrilic solution (3 mL) of 2-aminopyrimidine (22 mg). The reaction mixture after complete dissolution was stirred for 2 hours at 50 ˚C. The title compound was deposited directly from the mother liquor in a non-crystalline state. Obtained product was filtered off, washed with a small amount of acetonitrile and diethyl ether then dried in the air to afford (4-fluoro-3-nitrophenyl)(pyrimidin-2-ylamino)methanol—(55 mg, 90%), mp 116–117 °C.

3.9.2. Elemental Analysis

	% C	% H	% N
Calculated	50.00	3.43	21.20
Found	49.84	3.53	21.08

3.9.3. Mass Spectrometry

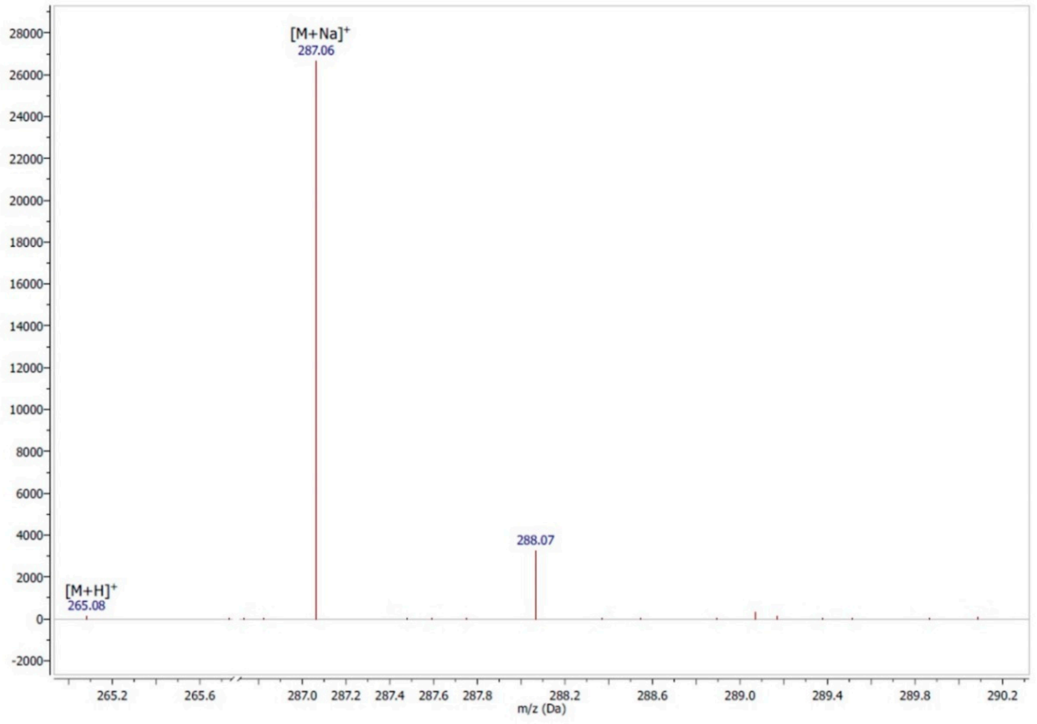

3.9.4. NMR Spectroscopy

^{1}H-NMR

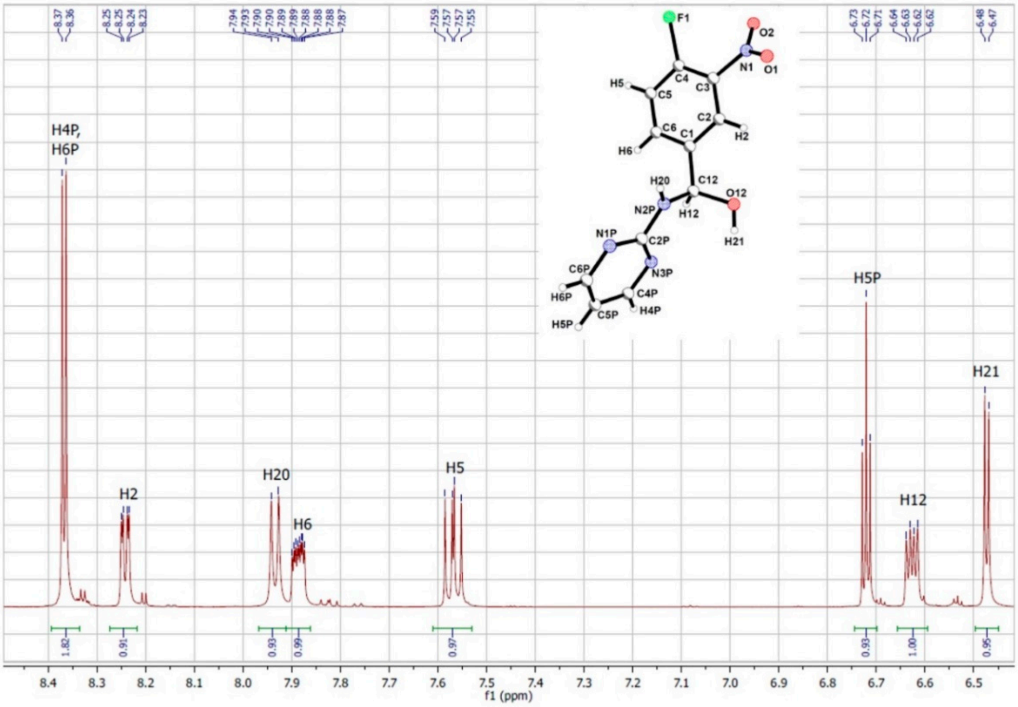

^{1}H-NMR (600 MHz, DMSO, 298 K) δ: 8.37 (d, $^{3}J_{\mathrm{H4P/H6P,H5P}}$ = 4.8, 2H, H4P, H6P), 8.24 (dd, $^{4}J_{\mathrm{H2,F}}$ = 7.5 Hz, $^{4}J_{\mathrm{H2,H6}}$ = 2.1 Hz, 1H, H2), 7.94 (d, $^{3}J_{\mathrm{H20,H12}}$ = 9.0 Hz, 1H, H20), 7.87–7.90 (m, 1H, H6), 7.55–7.59 (m, 1H, H5), 6.72 (t, $^{3}J_{\mathrm{H5P,H4P/H6P}}$ = 4.8 Hz, 1H, H5P), 6.63 (dd, $^{3}J_{\mathrm{H12,H20}}$ = 9.0 Hz, $^{3}J_{\mathrm{H12,H21}}$ = 4.8 Hz, 1H, H12), 6.47 (d, $^{3}J_{\mathrm{H21,H12}}$ = 4.8 Hz, 1H, H21).

^{13}C-NMR

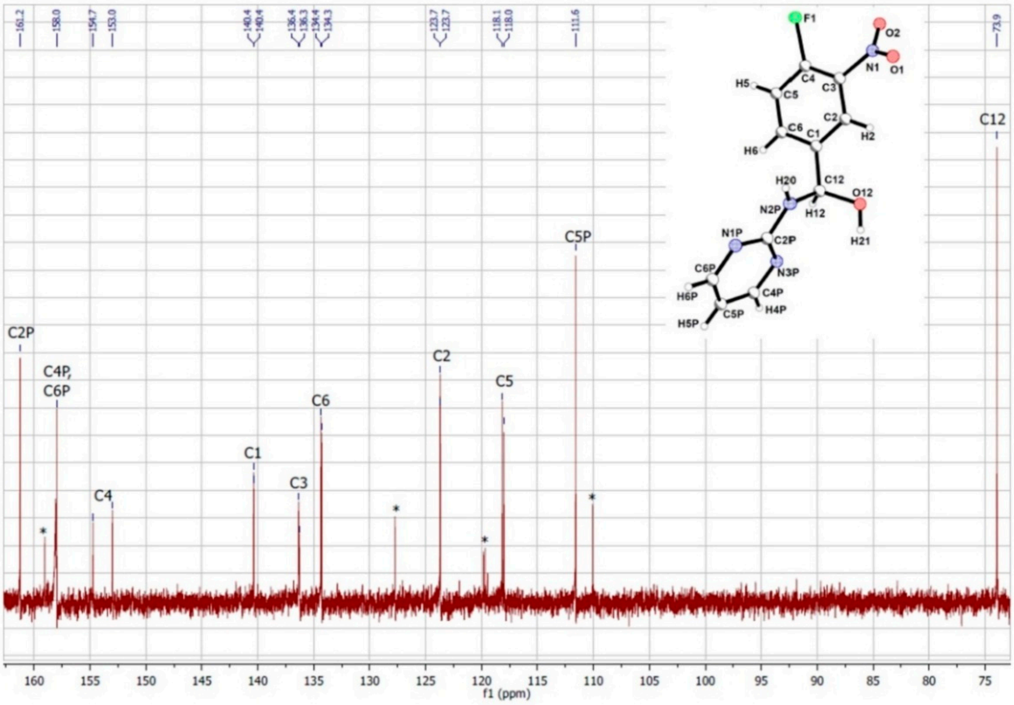

^{13}C-NMR (150.9 MHz, DMSO, 298 K) δ: 161.2 (C2P), 158.0 (C4P, C6P), 153.9 (d, $^{1}J_{C4,F}$ = 259.4 Hz, C4), 140.4 (d, $^{4}J_{C1,F}$ = 4.5 Hz, C1), 136.3 (d, $^{2}J_{C3,F}$ = 6.0 Hz, C3), 134.3 (d, $^{3}J_{C6,F}$ = 9.0 Hz, C6), 123.7 (d, $^{3}J_{C2,F}$ = 3.0 Hz, C2), 118.1 (d, $^{2}J_{C5,F}$ = 19.6 Hz, C5), 111.6 (C5P), 73.9 (C12).
*—Impurities, results of the fact that in the solution hemiaminals are in a dynamic equilibrium primarily with the initial reagents (aldehyde + amine), in some cases traces of an imine could also be observed.

COSY

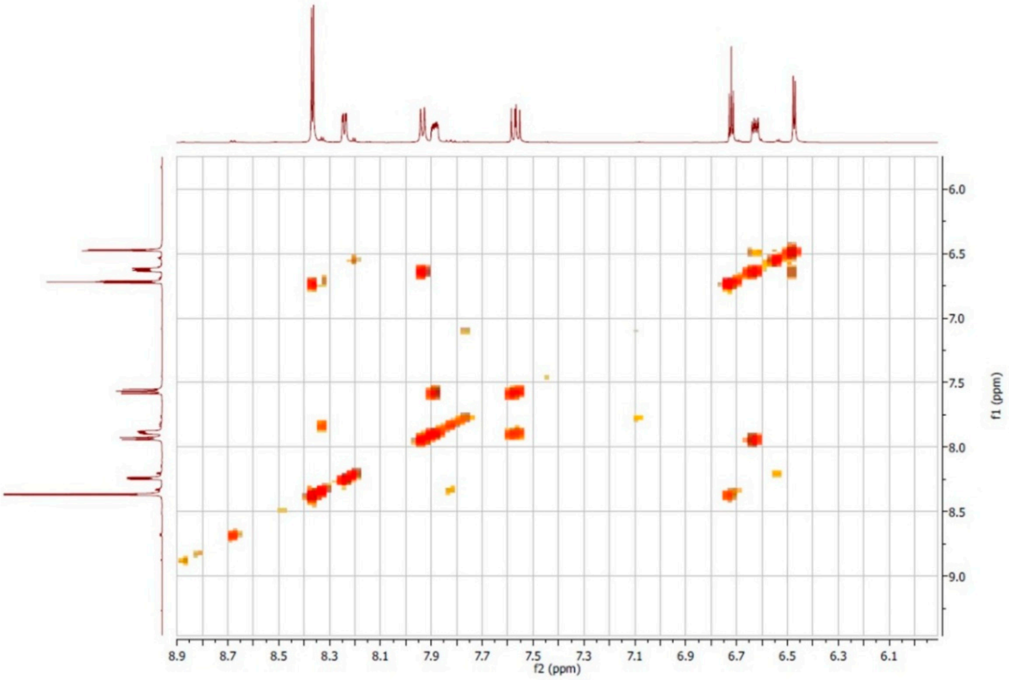

HMQC

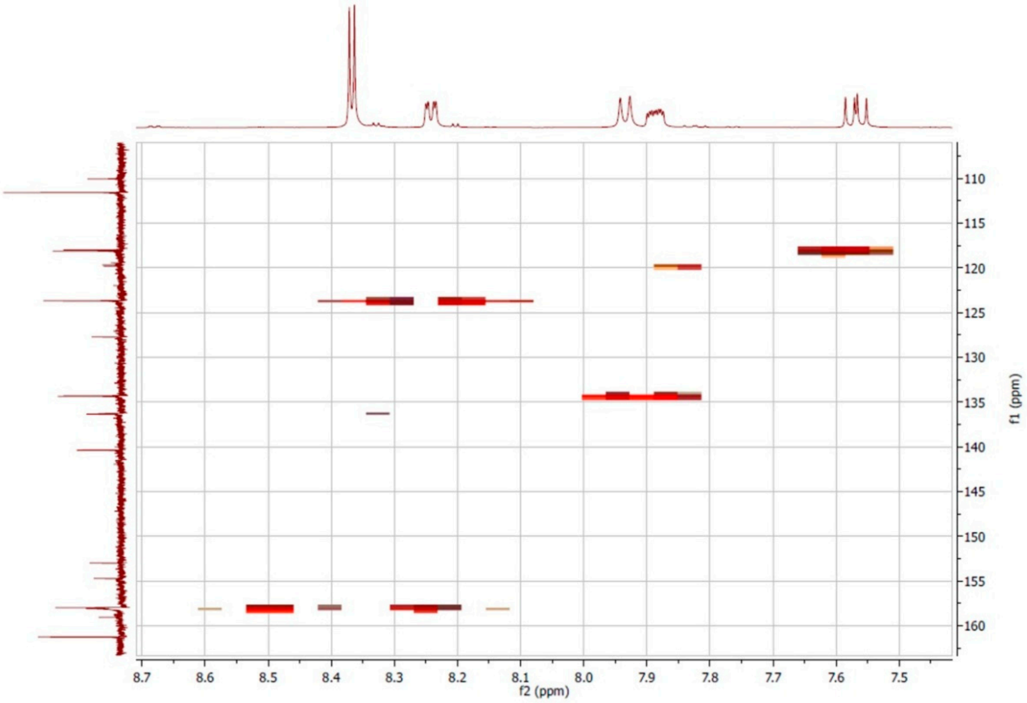

3.9.4.5. HMBC

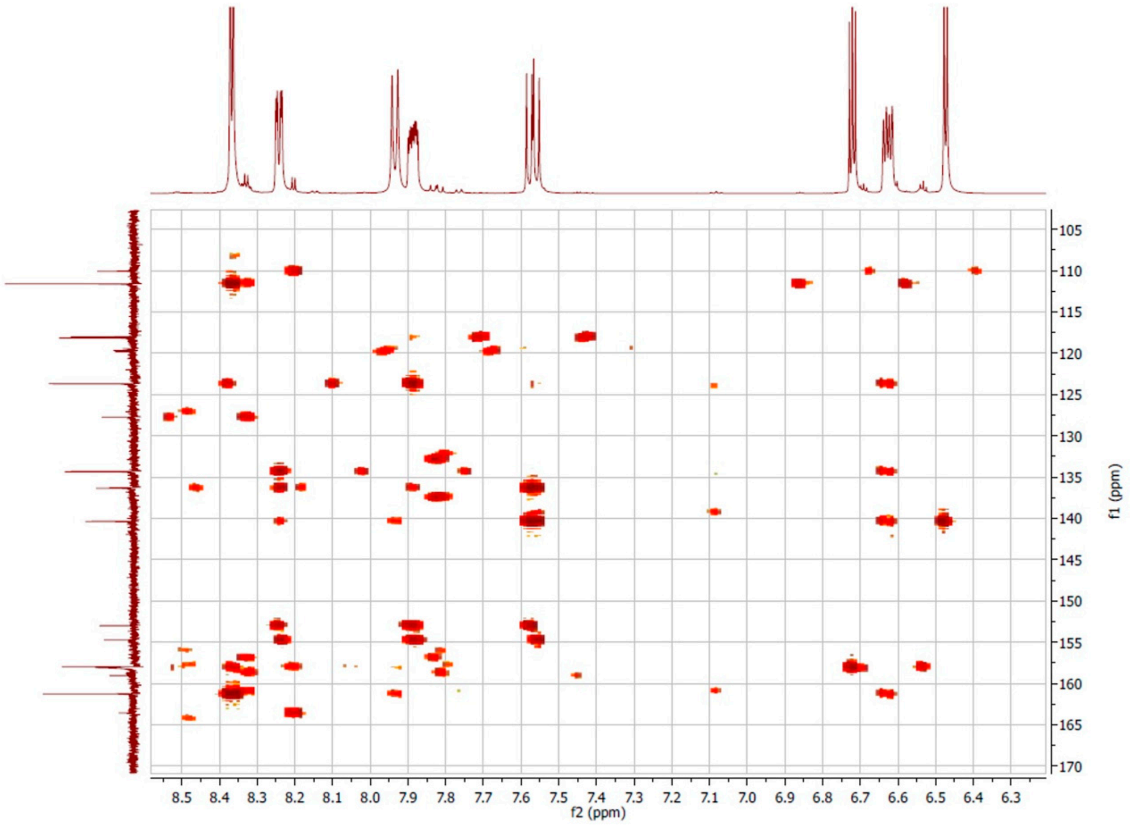

3.9.5. IR Spectroscopy

IR (KBr, cm^{-1}): 3329 vs, 3079 s, 3041 s, 2830 m, 2686 m, 2349 vw, 1965 vw, 1621 s, 1590 vs, 1572 vs, 1537 vs, 1519 vs, 1461 vs, 1424 s, 1349 vs, 1309 m, 1286 m, 1269 m, 1252 vs, 1238 s, 1199 w, 1181 w, 1150 w, 1117 s, 1092 s, 1086 s, 1071 s, 1027 vs, 997 m, 954 w, 926 w, 900 vw, 856 m, 824 m, 801 m, 761 w, 710 w, 673 m, 635 w, 597 m, 575 s, 530 w, 508 w, 473 vw, 393 vw.

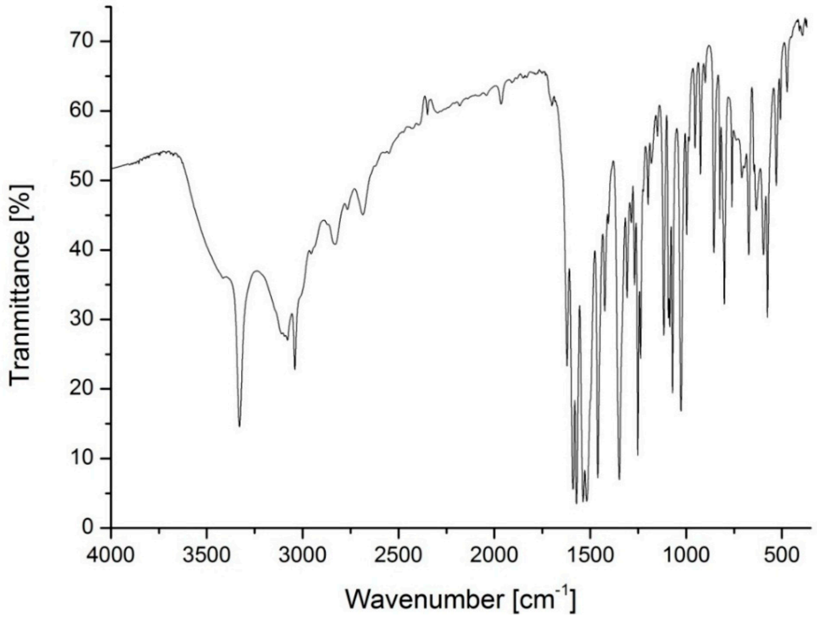

3.10. [2-Nitro-4-(trifluoromethyl)phenyl](pyrimidin-2-ylamino)methanol (10)

3.10.1. Synthesis

Acetonitrilic solution (3 mL) of 2-nitro-4-(trifluoromethyl)benzaldehyde (36 mg) was added to an acetonitrilic solution (3 mL) of 2-aminopyrimidine (16 mg). The reaction mixture after complete dissolution was stirred for 2 hours at 50 ˚C. The title compound crystallised directly from the mother liquor. Upon standing 3 days at the room temperature, the solution deposited pale yellow crystal needles. The crystals were filtered off, washed with a small amount of acetonitrile and diethyl ether then dried in the air to afford [2-nitro-4-(trifluoromethyl)phenyl](pyrimidin-2-ylamino)methanol—(45 mg, 87%), mp 105 °C.

3.10.2. Elemental Analysis

	% C	% H	% N
Calculated	45.87	2.89	17.83
Found	46.07	2.90	18.03

3.10.3. Mass Spectrometry

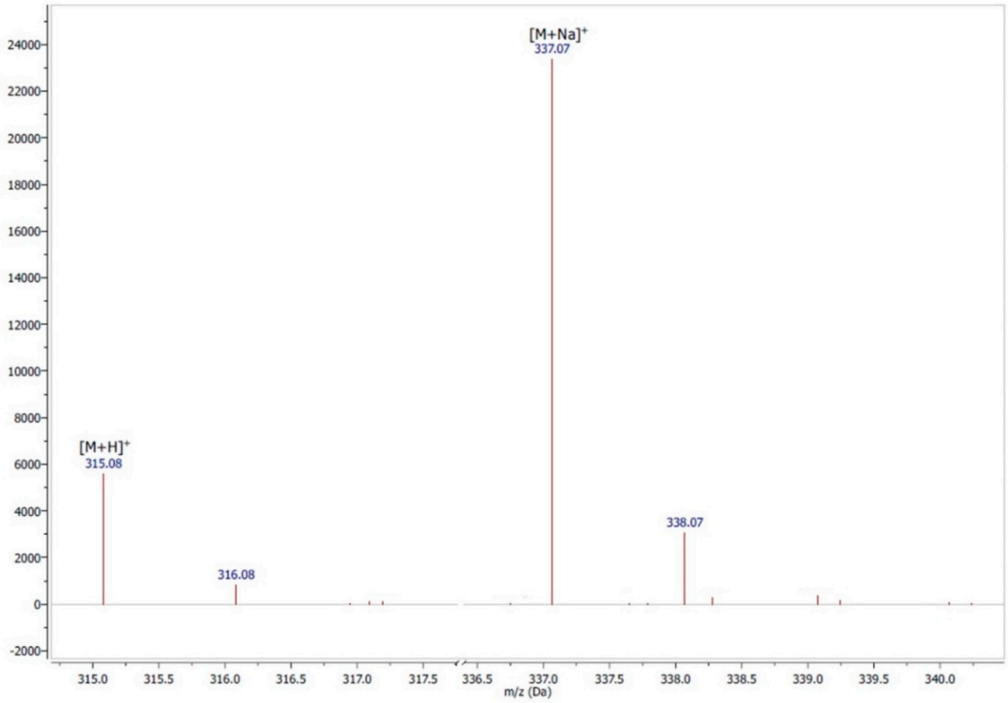

3.10.4. NMR Spectroscopy

^{1}H-NMR

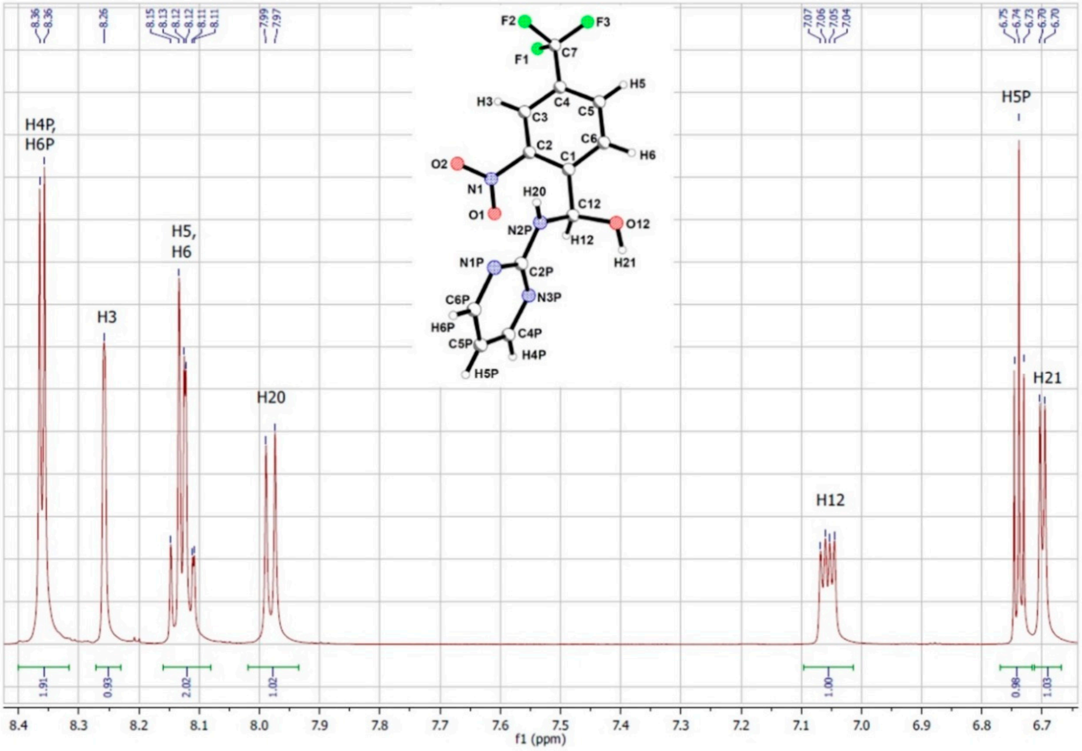

^{1}H-NMR (600 MHz, DMSO, 298 K) δ: 8.36 (d, $^{3}J_{H4P/H6P,H5P}$ = 4.8, 2H, H4, H6), 8.26 (s, 1H, H3), 8.11–8.15 (m, 2H, H5, H6), 7.98 (d, $^{3}J_{H20,H12}$ = 9.0 Hz, 1H, H20), 7.06 (dd, $^{3}J_{H12,H20}$ = 9.0 Hz, $^{3}J_{H12,H21}$ = 4.8 Hz, 1H, H12), 6.74 (t, $^{3}J_{H5P,H4P/H6P}$ = 4.8 Hz, 1H, H5P), 6.70 (d, $^{3}J_{H21,H12}$ = 4.8 Hz, 1H, H21).

^{13}C-NMR

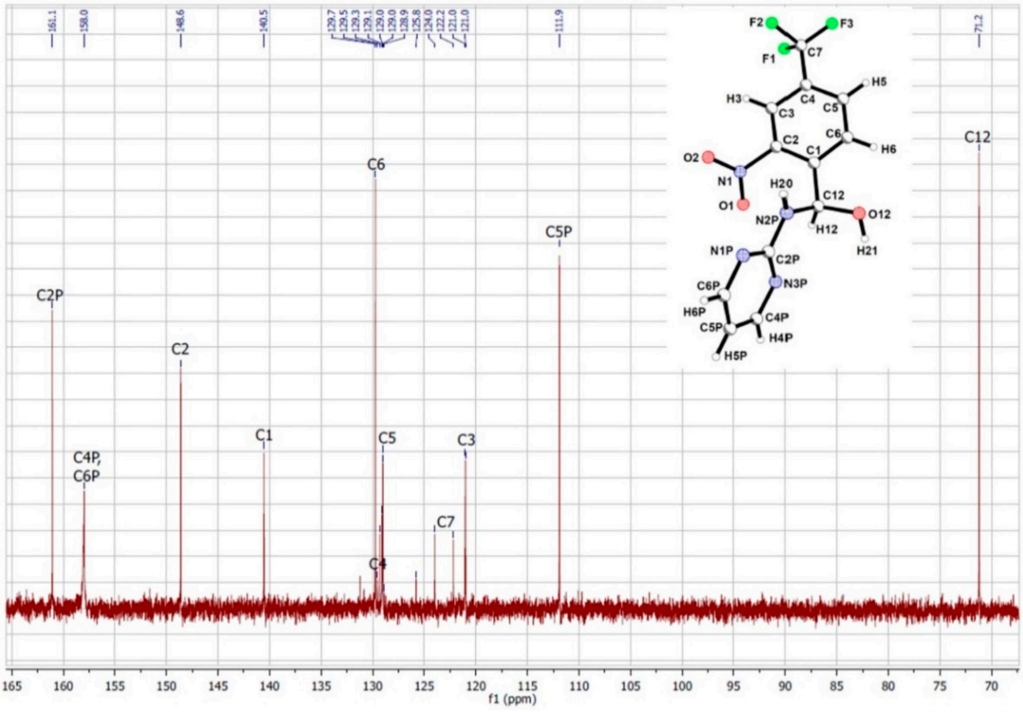

^{13}C-NMR (150.9 MHz, DMSO, 298 K) δ: 161.1 (C2P), 158.0 (C4P, C6P), 148.6 (C2), 140.5 (C1), 129.7 (C6), 129.2 (q, $^{2}J_{C4,F}$ = 33.2 Hz, C4), 129.0 (q, $^{3}J_{C5,F}$ = 4.5 Hz, C5), 123.1 (q, $^{1}J_{C7,F}$ = 272.9 Hz, C7), 121.0 (q, $^{3}J_{C3,F}$ = 3.0 Hz, C3), 111.9 (C5P), 71.2 (C12).

COSY

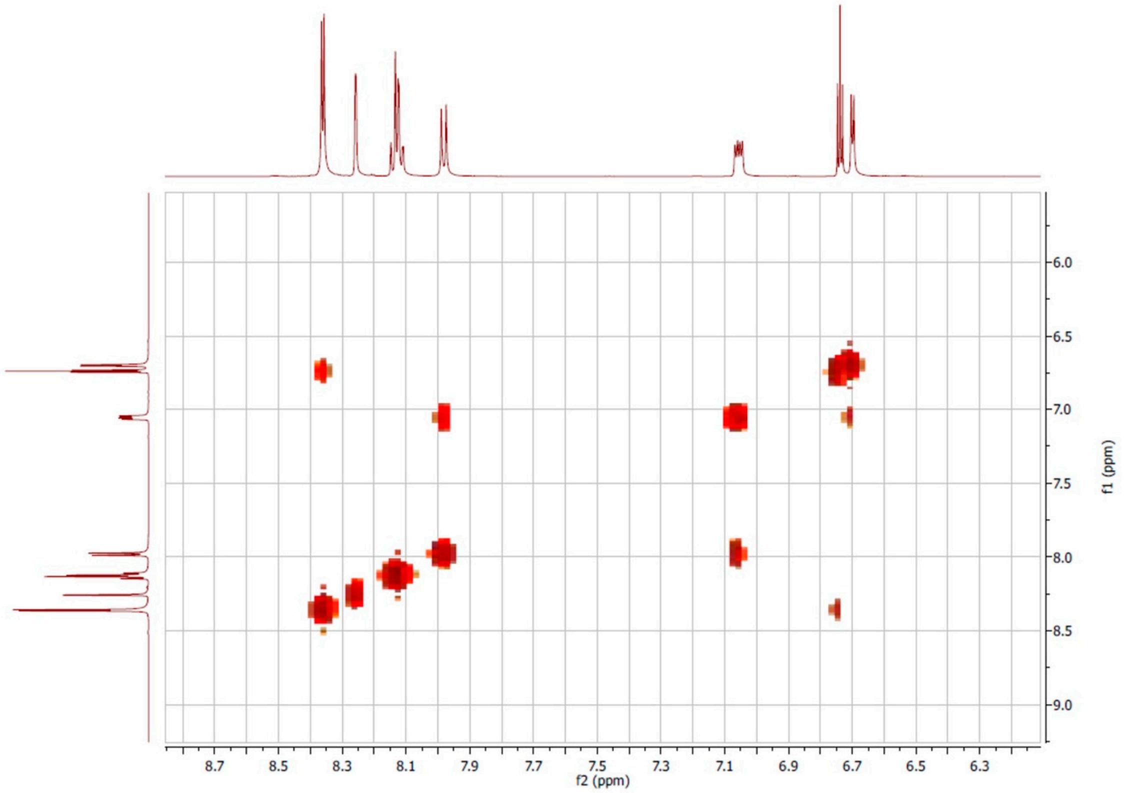

HMQC

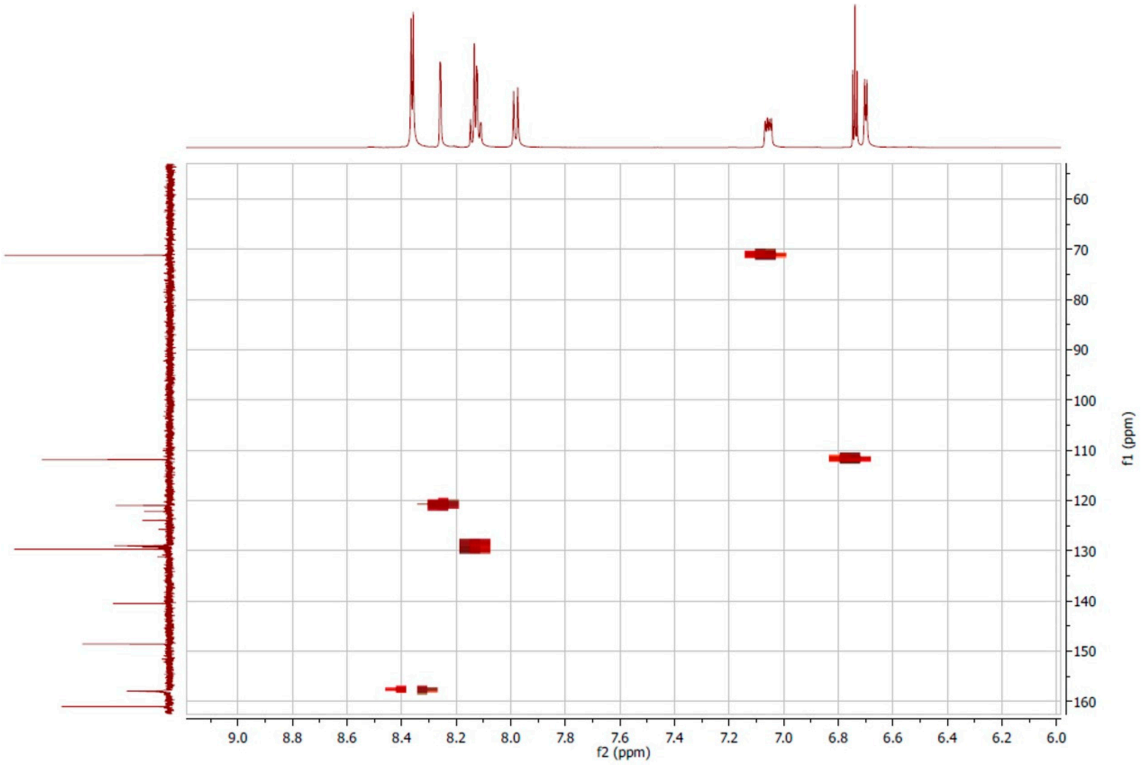

HMBC

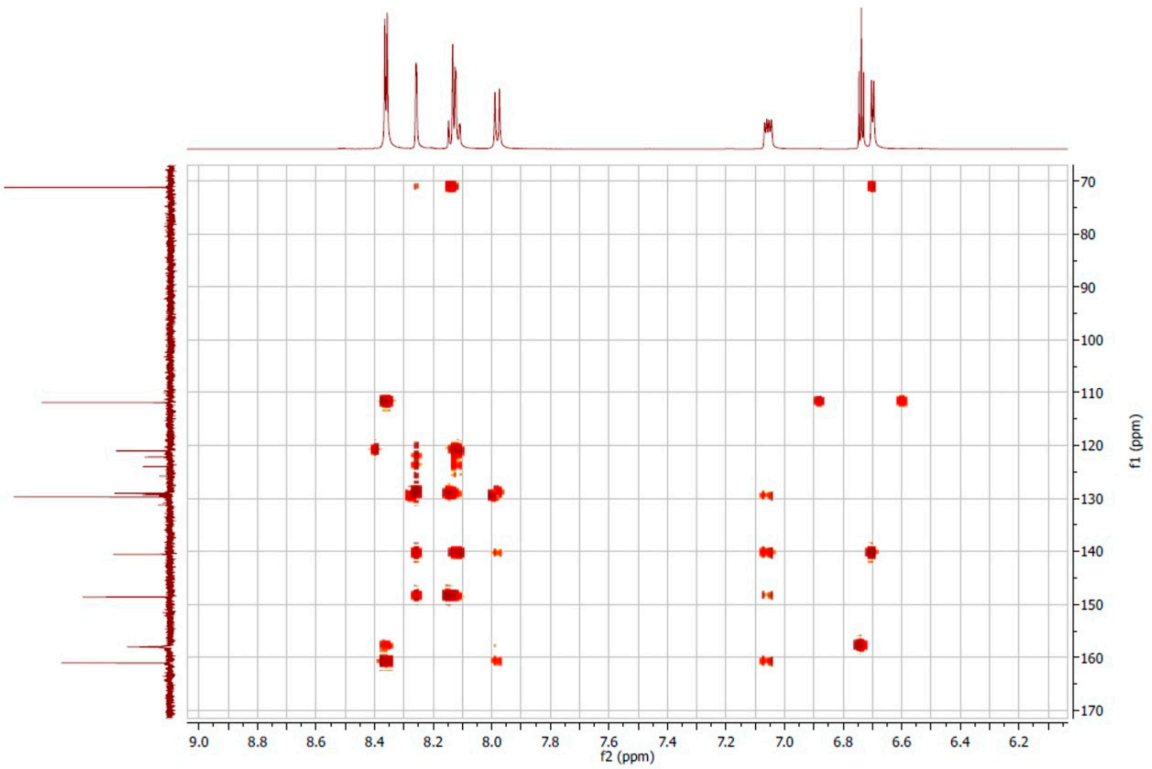

3.10.5. IR Spectroscopy

IR (KBr, cm^{-1}): 3413 s, 3106 m, 3057 m, 1632 w, 1591 vs, 1578 vs, 1536 vs, 1504 s, 1461 s, 1410 m, 1367 m, 1325 vs, 1308 m, 1286 w, 1244 m, 1201 w, 1182 s, 1151 m, 1127 s, 1091 m, 1077 s, 1066 m, 1043 s, 998 vw, 932 vw, 915 vw, 894 vw, 852 m, 830 w, 807 w, 784 w, 731 vw, 702 w, 675 vw, 649 w, 629 vw, 578 w, 517 w, 495 w, 458 w, 439 vw, 415 vw.

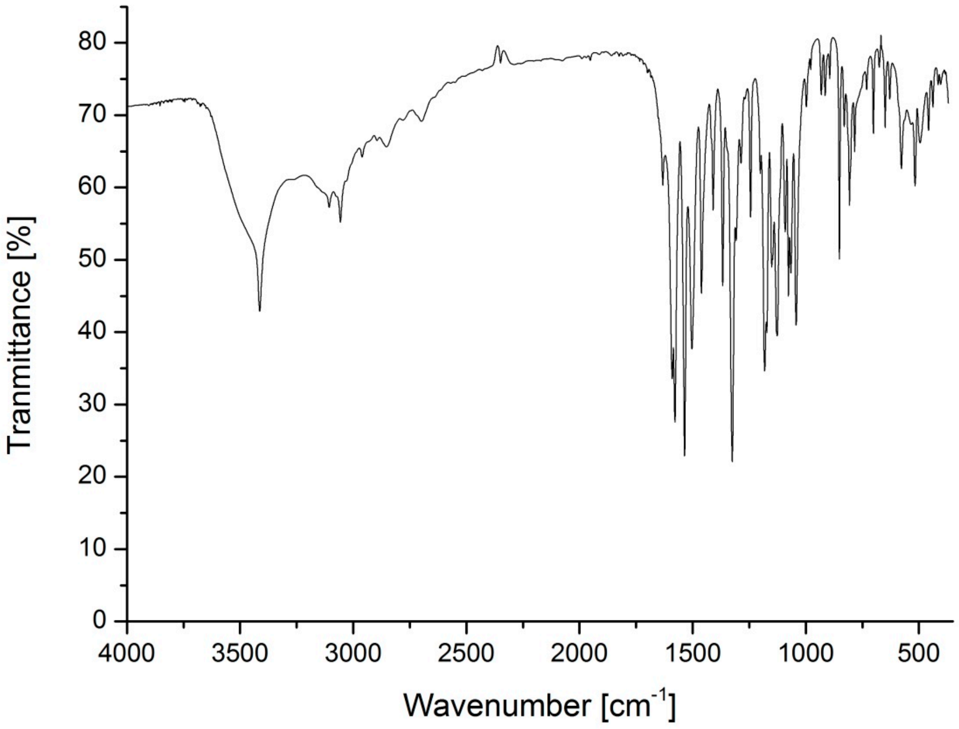

3.10.6. Crystallography

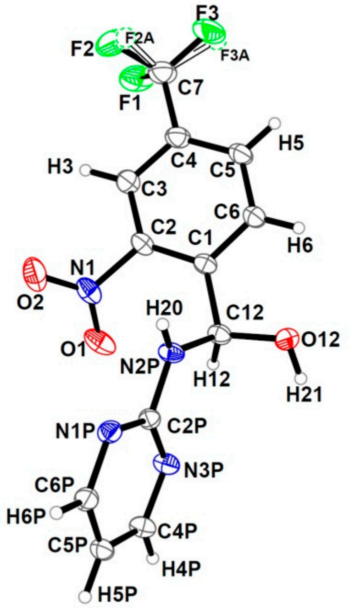

Figure 7. Molecular structure and labelling for [2-nitro-4-(trifluoromethyl)phenyl](pyrimidin-2-ylamino)methanol (10). Displacement ellipsoids are shown at the 50% probability level.

Table S3.10.6.1. Crystal data and structure refinement for 10.

**Identification Code**	**10**	
Empirical formula	C12 H9 F3 N4 O3	
Formula weight	314.23	
Temperature	100(2) K	
Wavelength	0.71073 Å	
Crystal system	Monoclinic	
Space group	C 1 2/c 1	
Unit cell dimensions	a = 38.572(9) Å	α = 90°
	b = 5.345(3) Å	β = 110.14(3)°
	c = 13.358(4) Å	γ = 90°
Volume	2585.6(18) Å^3	
Z	8	
Density (calculated)	1.614 Mg/m^3	
Absorption coefficient	0.147 mm^{-1}	
F(000)	1280	
Crystal size	0.70 × 0.20 × 0.12 mm^3	
Theta range for data collection	3.05 to 36.97°.	
Index ranges	$-64 \leq h \leq 64$, $-8 \leq k \leq 6$, $-21 \leq l \leq 21$	
Reflections collected	23,733	
Independent reflections	5929 [R(int) = 0.0305]	

Table S3.10.6.1. *Cont.*

Identification Code	10
Completeness to theta = 27.00	99.9%
Absorption correction	Semi-empirical from equivalents
Max. and min. transmission	1.00000 and 0.89433
Refinement method	Full-matrix least-squares on F^2
Data/restraints/parameters	5929/0/233
Goodness-of-fit on F^2	0.999
Final R indices [I > 2sigma(I)]	R1 = 0.0464, wR2 = 0.1176
R indices (all data)	R1 = 0.0727, wR2 = 0.1262
Largest diff. peak and hole	0.482 and −0.174 e.Å^{-3}

Table S3.10.6.2. Atomic coordinates (×10^4) and equivalent isotropic displacement parameters (Å^2 × 10^3) for 10. U(eq) is defined as one third of the trace of the orthogonalized U^{ij} tensor.

	x	y	z	U(eq)
C(1)	8887(1)	2954(2)	5115(1)	20(1)
C(2)	8821(1)	4658(2)	4277(1)	21(1)
C(3)	9063(1)	5077(2)	3737(1)	24(1)
C(4)	9382(1)	3638(2)	4014(1)	26(1)
C(5)	9457(1)	1901(2)	4829(1)	28(1)
C(6)	9214(1)	1596(2)	5387(1)	25(1)
N(1)	8473(1)	6077(2)	3894(1)	26(1)
O(1)	8185(1)	4922(2)	3744(1)	33(1)
O(2)	8490(1)	8311(2)	3718(1)	40(1)
C(7)	9636(1)	4017(2)	3394(1)	34(1)
F(1)	9465(2)	3627(14)	2340(4)	45(1)
F(2)	9729(1)	6461(7)	3375(4)	48(1)
F(3)	9952(1)	2879(13)	3805(5)	55(1)
F(1A)	9490(4)	3210(40)	2422(11)	59(3)
F(2A)	9787(5)	6120(30)	3523(15)	86(3)
F(3A)	9897(6)	2120(50)	3594(17)	79(4)
C(12)	8623(1)	2655(2)	5729(1)	20(1)
O(12)	8783(1)	1064(1)	6609(1)	24(1)
N(2P)	8546(1)	5121(1)	6047(1)	20(1)
N(1P)	8217(1)	7960(1)	6654(1)	21(1)
C(2P)	8222(1)	5722(2)	6200(1)	18(1)
N(3P)	7943(1)	4089(2)	5879(1)	22(1)
C(4P)	7630(1)	4802(2)	6020(1)	23(1)
C(5P)	7594(1)	7049(2)	6484(1)	23(1)
C(6P)	7902(1)	8576(2)	6796(1)	23(1)

Table S3.10.6.3. Bond lengths [Å] and angles [°] for 10.

C(1)-C(6)	1.3909(13)
C(1)-C(2)	1.3972(14)
C(1)-C(12)	1.5202(13)
C(2)-C(3)	1.3799(13)
C(2)-N(1)	1.4722(12)
C(3)-C(4)	1.3887(14)
C(3)-H(3)	0.9500
C(4)-C(5)	1.3839(15)
C(4)-C(7)	1.4987(14)
C(5)-C(6)	1.3917(13)
C(5)-H(5)	0.9500
C(6)-H(6)	0.9500
N(1)-O(2)	1.2237(14)
N(1)-O(1)	1.2241(12)
C(7)-F(2A)	1.253(12)
C(7)-F(1A)	1.298(11)
C(7)-F(3)	1.303(6)
C(7)-F(1)	1.349(6)
C(7)-F(2)	1.358(4)
C(7)-F(3A)	1.386(13)
C(12)-O(12)	1.4091(12)
C(12)-N(2P)	1.4475(13)
C(12)-H(12)	1.0000
O(12)-H(21)	0.871(15)
N(2P)-C(2P)	1.3695(11)
N(2P)-H(20)	0.895(13)
N(1P)-C(6P)	1.3340(12)
N(1P)-C(2P)	1.3447(13)
C(2P)-N(3P)	1.3373(12)
N(3P)-C(4P)	1.3391(12)
C(4P)-C(5P)	1.3802(14)
C(4P)-H(4P)	0.9500
C(5P)-C(6P)	1.3818(14)
C(5P)-H(5P)	0.9500
C(6P)-H(6P)	0.9500
C(6)-C(1)-C(2)	116.67(9)
C(6)-C(1)-C(12)	121.33(8)
C(2)-C(1)-C(12)	121.97(8)
C(3)-C(2)-C(1)	123.68(8)
C(3)-C(2)-N(1)	115.81(8)
C(1)-C(2)-N(1)	120.48(8)
C(2)-C(3)-C(4)	117.88(9)
C(2)-C(3)-H(3)	121.1
C(4)-C(3)-H(3)	121.1
C(5)-C(4)-C(3)	120.45(9)

Table S3.10.6.3. *Cont.*

C(5)-C(4)-C(7)	121.75(9)
C(3)-C(4)-C(7)	117.79(9)
C(4)-C(5)-C(6)	120.23(9)
C(4)-C(5)-H(5)	119.9
C(6)-C(5)-H(5)	119.9
C(1)-C(6)-C(5)	121.01(9)
C(1)-C(6)-H(6)	119.5
C(5)-C(6)-H(6)	119.5
O(2)-N(1)-O(1)	124.63(9)
O(2)-N(1)-C(2)	117.69(9)
O(1)-N(1)-C(2)	117.65(9)
F(2A)-C(7)-F(1A)	117.3(15)
F(2A)-C(7)-F(3)	92.1(10)
F(1A)-C(7)-F(3)	106.7(10)
F(2A)-C(7)-F(1)	108.6(10)
F(3)-C(7)-F(1)	114.7(4)
F(1A)-C(7)-F(2)	109.1(10)
F(3)-C(7)-F(2)	104.0(3)
F(1)-C(7)-F(2)	99.6(4)
F(2A)-C(7)-F(3A)	110.9(7)
F(1A)-C(7)-F(3A)	89.3(19)
F(1)-C(7)-F(3A)	98.6(12)
F(2)-C(7)-F(3A)	121.8(12)
F(2A)-C(7)-C(4)	114.0(5)
F(1A)-C(7)-C(4)	111.4(7)
F(3)-C(7)-C(4)	113.5(3)
F(1)-C(7)-C(4)	112.2(3)
F(2)-C(7)-C(4)	111.6(2)
F(3A)-C(7)-C(4)	111.4(5)
O(12)-C(12)-N(2P)	112.32(8)
O(12)-C(12)-C(1)	109.37(7)
N(2P)-C(12)-C(1)	107.85(7)
O(12)-C(12)-H(12)	109.1
N(2P)-C(12)-H(12)	109.1
C(1)-C(12)-H(12)	109.1
C(12)-O(12)-H(21)	106.4(9)
C(2P)-N(2P)-C(12)	123.19(7)
C(2P)-N(2P)-H(20)	113.3(8)
C(12)-N(2P)-H(20)	121.0(8)
C(6P)-N(1P)-C(2P)	116.07(8)
N(3P)-C(2P)-N(1P)	126.17(8)
N(3P)-C(2P)-N(2P)	118.28(8)

Table S3.10.6.3. *Cont.*

N(1P)-C(2P)-N(2P)	115.55(8)
C(2P)-N(3P)-C(4P)	115.73(8)
N(3P)-C(4P)-C(5P)	123.08(9)
N(3P)-C(4P)-H(4P)	118.5
C(5P)-C(4P)-H(4P)	118.5
C(4P)-C(5P)-C(6P)	116.15(8)
C(4P)-C(5P)-H(5P)	121.9
C(6P)-C(5P)-H(5P)	121.9
N(1P)-C(6P)-C(5P)	122.78(9)
N(1P)-C(6P)-H(6P)	118.6
C(5P)-C(6P)-H(6P)	118.6

Symmetry transformations used to generate equivalent atoms.

Table S3.10.6.4. Anisotropic displacement parameters ($Å^2 \times 10^3$)for 10. The anisotropic displacement factor exponent takes the form: $-2\pi^2[h^2a^{*2}U^{11} + ... + 2 h k a^* b^* U^{12}]$.

	U^{11}	U^{22}	U^{33}	U^{23}	U^{13}	U^{12}
C(1)	17(1)	22(1)	20(1)	−3(1)	7(1)	1(1)
C(2)	19(1)	25(1)	18(1)	−3(1)	5(1)	5(1)
C(3)	24(1)	30(1)	18(1)	1(1)	7(1)	3(1)
C(4)	21(1)	35(1)	23(1)	2(1)	10(1)	2(1)
C(5)	20(1)	36(1)	30(1)	5(1)	12(1)	8(1)
C(6)	21(1)	28(1)	26(1)	5(1)	10(1)	6(1)
N(1)	26(1)	33(1)	18(1)	−2(1)	6(1)	11(1)
O(1)	20(1)	47(1)	29(1)	−9(1)	3(1)	7(1)
O(2)	46(1)	33(1)	39(1)	8(1)	14(1)	17(1)
C(7)	26(1)	49(1)	31(1)	6(1)	15(1)	2(1)
F(1)	40(1)	71(2)	30(1)	2(1)	21(1)	−2(1)
F(2)	46(1)	46(2)	62(1)	6(1)	31(1)	−8(2)
F(3)	34(1)	80(2)	63(1)	26(2)	33(1)	21(1)
F(1A)	66(6)	86(6)	40(4)	−33(4)	38(4)	−34(4)
F(2A)	81(5)	128(8)	75(5)	−48(5)	57(4)	−74(5)
F(3A)	77(6)	102(8)	89(7)	64(6)	70(6)	65(5)
C(12)	19(1)	20(1)	23(1)	−4(1)	8(1)	0(1)
O(12)	24(1)	24(1)	29(1)	3(1)	13(1)	0(1)
N(2P)	17(1)	21(1)	24(1)	−6(1)	9(1)	−2(1)
N(1P)	26(1)	18(1)	23(1)	−2(1)	14(1)	0(1)
C(2P)	19(1)	19(1)	16(1)	0(1)	7(1)	1(1)
N(3P)	18(1)	23(1)	28(1)	−6(1)	9(1)	−1(1)
C(4P)	17(1)	28(1)	25(1)	−2(1)	8(1)	0(1)
C(5P)	21(1)	26(1)	23(1)	3(1)	11(1)	6(1)
C(6P)	29(1)	20(1)	26(1)	0(1)	16(1)	3(1)

Table S3.10.6.5. Hydrogen coordinates (×10^4^) and isotropic displacement parameters (Å^2 × 10^3) for 10.

	x	y	z	U(eq)
H(3)	9013	6310	3193	29
H(5)	9674	914	5009	33
H(6)	9273	442	5962	29
H(12)	8388	1890	5250	24
H(21)	8603(4)	190(30)	6686(11)	36
H(20)	8728(4)	6210(20)	6351(10)	24
H(4P)	7423	3711	5790	28
H(5P)	7371	7518	6584	27
H(6P)	7889	10131	7125	28

Table S3.10.6.6. Torsion angles [°] for 10.

C(6)-C(1)-C(2)-C(3)	−1.37(14)
C(12)-C(1)-C(2)-C(3)	176.52(8)
C(6)-C(1)-C(2)-N(1)	176.61(8)
C(12)-C(1)-C(2)-N(1)	−5.49(13)
C(1)-C(2)-C(3)-C(4)	3.03(14)
N(1)-C(2)-C(3)-C(4)	−175.05(8)
C(2)-C(3)-C(4)-C(5)	−1.99(15)
C(2)-C(3)-C(4)-C(7)	177.37(9)
C(3)-C(4)-C(5)-C(6)	−0.57(16)
C(7)-C(4)-C(5)-C(6)	−179.90(10)
C(2)-C(1)-C(6)-C(5)	−1.33(14)
C(12)-C(1)-C(6)-C(5)	−179.24(9)
C(4)-C(5)-C(6)-C(1)	2.30(16)
C(3)-C(2)-N(1)-O(2)	−45.98(12)
C(1)-C(2)-N(1)-O(2)	135.88(10)
C(3)-C(2)-N(1)-O(1)	131.81(9)
C(1)-C(2)-N(1)-O(1)	−46.33(12)
C(5)-C(4)-C(7)-F(2A)	−114.2(13)
C(3)-C(4)-C(7)-F(2A)	66.4(13)
C(5)-C(4)-C(7)-F(1A)	110.2(11)
C(3)-C(4)-C(7)-F(1A)	−69.2(11)
C(5)-C(4)-C(7)-F(3)	−10.4(3)
C(3)-C(4)-C(7)-F(3)	170.2(3)
C(5)-C(4)-C(7)-F(1)	121.7(4)
C(3)-C(4)-C(7)-F(1)	−57.7(4)
C(5)-C(4)-C(7)-F(2)	−127.5(2)
C(3)-C(4)-C(7)-F(2)	53.1(3)
C(5)-C(4)-C(7)-F(3A)	12.2(15)
C(3)-C(4)-C(7)-F(3A)	−167.1(15)
C(6)-C(1)-C(12)-O(12)	5.46(11)

Table S3.10.6.6. *Cont.*

C(2)-C(1)-C(12)-O(12)	−172.34(8)
C(6)-C(1)-C(12)-N(2P)	127.89(9)
C(2)-C(1)-C(12)-N(2P)	−49.91(11)
O(12)-C(12)-N(2P)-C(2P)	−87.68(10)
C(1)-C(12)-N(2P)-C(2P)	151.72(8)
C(6P)-N(1P)-C(2P)-N(3P)	0.27(13)
C(6P)-N(1P)-C(2P)-N(2P)	179.30(8)
C(12)-N(2P)-C(2P)-N(3P)	−10.99(13)
C(12)-N(2P)-C(2P)-N(1P)	169.89(8)
N(1P)-C(2P)-N(3P)-C(4P)	0.82(13)
N(2P)-C(2P)-N(3P)-C(4P)	−178.19(8)
C(2P)-N(3P)-C(4P)-C(5P)	−1.22(14)
N(3P)-C(4P)-C(5P)-C(6P)	0.55(14)
C(2P)-N(1P)-C(6P)-C(5P)	−1.02(13)
C(4P)-C(5P)-C(6P)-N(1P)	0.64(14)

Symmetry transformations used to generate equivalent atoms.

Table S3.10.6.7. Hydrogen bonds for 10 [Å and °].

D-H...A	d(D-H)	d(H...A)	d(D...A)	<(DHA)
O(12)-H(21)...N(1P)#1	0.871(15)	1.896(15)	2.7581(13)	170.0(14)
N(2P)-H(20)...O(12)#2	0.895(13)	2.617(13)	3.319(2)	135.9(10)
N(2P)-H(20)...F(1)#3	0.895(13)	2.692(14)	3.434(6)	140.9(10)
C(3)-H(3)...O(12)#4	0.95	2.44	3.3740(16)	168.7
C(6P)-H(6P)...O(2)#5	0.95	2.68	3.2392(17)	118.0
C(5P)-H(5P)...O(1)#6	0.95	2.45	3.3347(14)	154.4
C(4P)-H(4P)...N(3P)#7	0.95	2.65	3.4331(17)	139.8

Symmetry transformations used to generate equivalent atoms: #1 x,y-1,z #2 x,y+1,z #3 x,-y+1,z+1/2 #4 x,-y+1,z-1/2 #5 x,-y+2,z+1/2 #6 -x+3/2,-y+3/2,-z+1 #7 -x+3/2,-y+1/2,-z+1 .

4. References

1. Cosier, J.; Glazer, A.M. A nitrogen-gas-stream cryostat for general X-ray diffraction studies. *J. Appl. Cryst.* **1986**, *19*, 105–107.
2. CrysAlis CCD, CrysAlis RED, CrysAlisPRO, Oxford Diffraction/Agilent Technologies UK Ltd.: Yarnton, England, UK.
3. Sheldrick, G.M. A short history of *SHELX*. *Acta Crystallogr. Sect. A: Found. Crystallogr.* **2008**, *64*, 112–122.
4. XP—INTERACTIVE MOLECULAR GRAPHICS, v. 5.1; Bruker Analytical X-ray System, 1998.
5. Spek, A.L. Single-crystal structure validation with the program *PLATON*. *J. Appl. Cryst.* **2003**, *36*, 7–13.
